# Palladium mediated domino reaction: synthesis of isochromenes under aqueous medium†

**DOI:** 10.1039/c9ra08792c

**Published:** 2020-01-02

**Authors:** Lavisha Punia, Karu Ramesh, Gedu Satyanarayana

**Affiliations:** Department of Chemistry, Indian Institute of Technology (IIT) Hyderabad Kandi – 502 285, Sangareddy District Telangana India gvsatya@iith.ac.in https://sites.google.com/site/gsresearchgrouphomepage/home +91 40 2301 6032

## Abstract

Isochromenes have been synthesized using palladium-catalyzed C–C and C–O bond forming reactions starting from *ortho*-bromo tertiary benzylic alcohols and internal acetylenes. Notably, this domino process is feasible by using the green solvent, water. The protocol exhibited a broad substrate scope and afforded various isochromenes.

## Abstract 2

Isochromenes have been synthesized using palladium-catalyzed C–C and C–O bond forming reactions starting from *ortho*-bromo tertiary benzylic alcohols and internal acetylenes.

## Graphical abstract

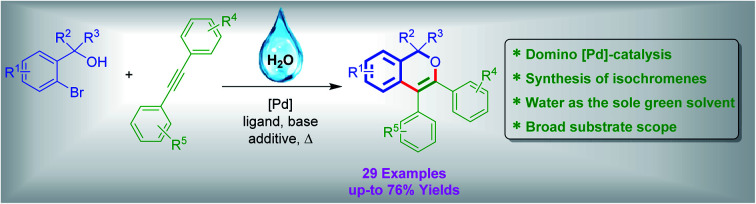

## Introduction

**Fig. 1:**
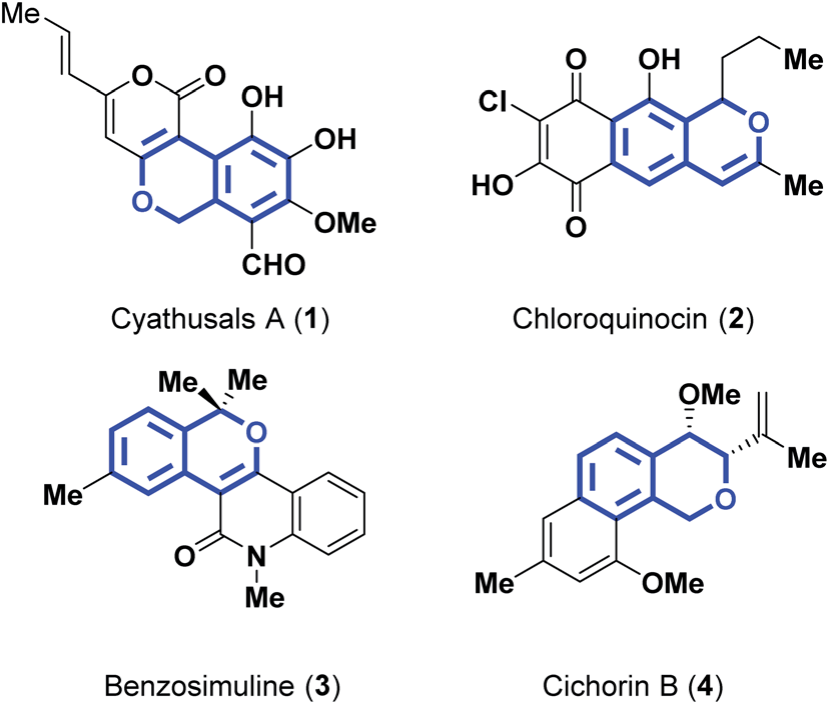
Isochromene based natural products.

Domino reactions are effective in the construction of two or more bonds and thereby facilitate obtaining products with reasonable structural complexity, under a particular set of reaction conditions, which may be devoid of additional reagents/catalysts.^1^ Moreover, in such a reaction, there is no need to isolate the intermediate product(s). Mainly, domino reactions play an important role in achieving complex hetero- and carbo-cyclic products, which are of great importance in the realm of biologically active molecules. Amongst them, fused bicyclic ethers (isochromenes) consisting of an aromatic ring and a pyran moiety are widely available heterocyclic motifs comprising natural products of biological significance.^2^ They are often identified in bacteria, fungi, plants, *etc.*^3^ and exhibit useful biological properties, such as antitumor,^4^ anticancer,^5^ antifungal,^6^ antibacterial, antibiotic,^7^ and antimicrobial activities.^8^ Some of the representative examples of natural products are as depicted in Fig. 1.^9–12^ Due to their interesting structural features and biological properties, isochromenes have captured the attention of chemists.^13^ As a consequence, a good number of synthetic routes were developed towards the synthesis of isochromenes.^14^ Especially, palladium-catalyzed annulation using relatively more reactive *ortho*-iodo tertiary benzylic alcohols with internal alkynes was also reported.^15^ These annulations were also driven by means of a stable palladacycle catalyst, as reported in ref. 16. Nevertheless, very few examples have been synthesized in these above two reports. In this regard, to the best of our knowledge, there are no reports of, particularly, using relatively less sensitive *ortho*-bromo tertiary benzylic alcohols catalyzed by a simple palladium-based catalyst and by employing water as the whole solvent medium, for the synthesis of isochromenes. In our efforts to establish metal-catalyzed coupling reactions,^17^ herein, we describe the synthesis of isochromenes by utilizing the solvent water as the reaction medium.

## Results and discussion

The optimization was begun with *ortho*-bromo tertiary benzylic alcohol 1a and diphenylacetylene 2a. In general, quaternary ammonium salts (QX; X = Cl, Br, I and HSO_4_) can act as a phase transfer catalyst mostly when the solvent is water or also can act as an additive to accelerate the catalytic activity of metal-catalyst.^18^ With this viewpoint in our mind, firstly, the alcohol 1a and diphenylacetylene 2a were treated at different temperatures using Pd(OAc)_2_ catalyst (5 mol%), tetrabutylammonium iodide (TBAI) (1 equiv.), base K_2_CO_3_ (4 equiv.) and with different ligands (entries 1 to 3, Table 1). Conversely, no progress of the reaction was noted. While treating 1a and 2a by using Xantphos ligand at 140 °C for 36 h, resulted the expected chromene 3aa, although in poor yield (entry 4, Table 1). Gratifyingly, the combination of Pd(OAc)_2_ and l-proline, TBAI, and base K_2_CO_3_ resulted in 76% yield of the desired chromene 3aa (entry 5, Table 1). Whereas other attempts with the combination of tetrabutylammonium iodide (TBAI/Na_2_CO_3_, TBAI/Li_2_CO_3_) and benzyltriethylammonium chloride (BTEAC)/K_2_CO_3_ in the presence of l-proline as the ligand were not that much efficient (entries 6 to 8, Table 1). Further, decreasing the quantity of solvent (0.2 mL of water), gave 3aa in 50% yield (entry 9, Table 1). While with 0.1 mL solvent water (entry 10, Table 1) or under neat reaction conditions (entry 11, Table 1), lead to the decomposition. On the other hand, when Na_2_CO_3_ (2 equiv.) was used as the base, there was no initiation in the reaction (entry 12, Table 1). While 30% yield of 3aa was formed with 2 equiv. of K_2_CO_3_ (entry 13, Table 1). Treatment of 1a and 2a with 4 equiv. of Et_3_N, lead to the decomposition (entry 14, Table 1).

**Table 1:** Optimizations of Pd-catalyzed domino reaction between substituted *ortho*-bromo tertiary benzylic alcohol 1a and internal alkyne 2a^a^^,^^b^^,^^c^^,^^d^^,^^e^^,^^f^^,^^g^

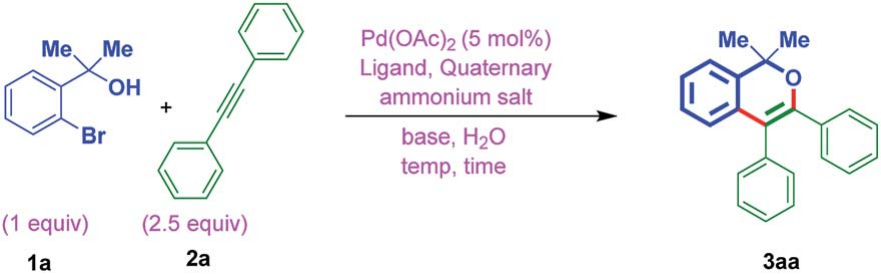					
Entry	Ligand (20 mol%)	Quaternary ammonium salt (equiv.) & base (equiv.)	Temp. (°C)	Time (h)	Yield of 3aa^a^ (%)
1	BINAP	TBAI (1) + K_2_CO_3_ (4)	140	36	—b
2	2,2′-Bipy	TBAI (1) + K_2_CO_3_(4)	120	38	—b
3	P(Cy)_3_	TBAI (1) + K_2_CO_3_ (4)	100	42	—b
4	Xantphos	TBAI (1) + K_2_CO_3_ (4)	140	36	30
**5**	**l** **-Proline**	**TBAI (1) + K_2_CO_3_ (4)**	**140**	**36**	**76**
6	l-Proline	TBAI (1) + Na_2_CO_3_ (4)	140	38	20
7	l-Proline	TBAI (1) + Li_2_CO_3_ (4)	140	36	15
8	l-Proline	BTEAC (1) + K_2_CO_3_ (4)	140	36	15
9^c^	l-Proline	TBAI (1) + K_2_CO_3_ (4)	140	36	50
10^d^	l-Proline	TBAI (1) + K_2_CO_3_ (4)	140	36	—b
11^e^	l-Proline	TBAI (1) + K_2_CO_3_ (4)	140	36	—b
12	l-Proline	TBAI (1) + Na_2_CO_3_ (2)	140	46	—f
13	l-Proline	TBAI (1) + K_2_CO_3_ (2)	140	42	30
14	l-Proline	TBAI (1) + NEt_3_ (4)	140	36	—b

a Conditions: 1a (54 mg, 0.25 mmol), 2a (111.2 mg, 0.625 mmol), Pd(OAc)_2_ (2.8 mg, 0.012 mmol), base (1 mmol), ligand (0.05 mmol), quaternary ammonium salt (0.25 mmol), water (0.5 mL), 140 °C, 36 h.

b Isolated yields of the product 3aa.

c Lead to the decomposition.

d Used 0.2 mL of water.

e The reaction was performed using 0.1 mL of water.

f Used neat reaction conditions.

g Starting materials were recovered. TBAI: tetrabutylammonium iodide. BTEAC: benzyltriethylammonium chloride.

With the above best-optimized conditions (entry 5, Table 1), to test the feasibility of the process, it was decided to test the protocol with other substrates. Thus, *ortho*-bromo tertiary benzylic alcohols 1a–1c were reacted with different internal alkynes 2a–2k, under standard conditions. Interestingly, this protocol was quite successful and afforded corresponding isochromenes 3aa–3cg (Table 2). In addition, the protocol exhibited good compatibility with various substituents bearing aromatic rings of acetylenes. For example, the reaction was also smooth on internal alkyne flanked to a heteroaromatic ring (3ad, Table 2). Also, furnished the products 3ah and 3ai bearing electron deactivating F and CF_3_ moieties. Besides, when *ortho*-bromo tertiary benzylic alcohol 1a was subjected to the reaction using unsymmetrical acetylenes 2j and 2k, as anticipated, furnished regioisomeric mixtures [(3aj : 3aj′ in 4 : 3 ratio) and (3ak : 3ak′ in 1 : 1 ratio) (Table 2)].

**Table 2:** Scope to generate isochromenes 3aa–3cg from *ortho*-bromo tertiary benzylic alcohols 1a–1c and internal alkynes 2a–2k^a^^,^^b^^,^^c^

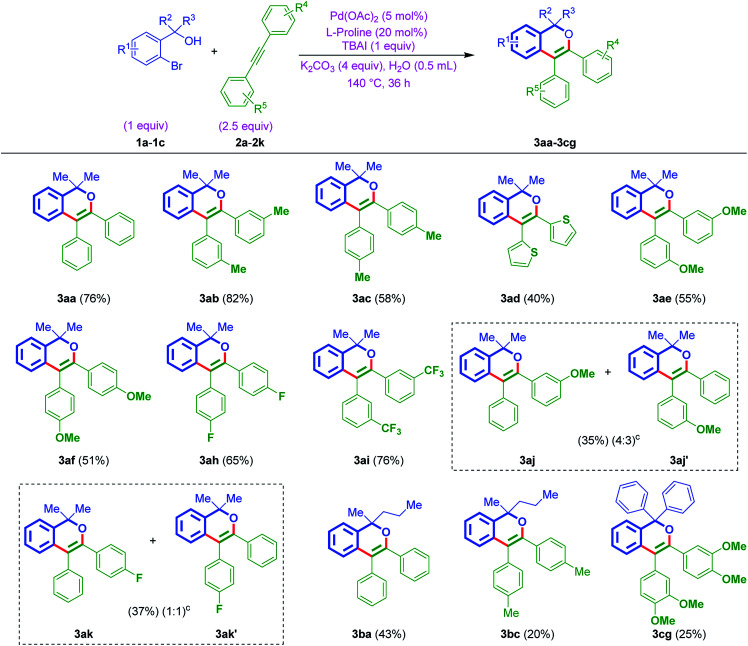

a Conditions: 1a–1c (0.25 mmol, 1 equiv.), 2a–2k (0.625 mmol, 2.5 equiv.), Pd(OAc)_2_ (2.8 mg, 0.012 mmol, 5 mol%), K_2_CO_3_ (138.6 mg, 1 mmol, 4 equiv.), l-proline (5.6 mg, 0.05 mol, 20 mol%), TBAI (tetrabutylammonium iodide) (92.3 mg, 0.25 mmol, 1 equiv.), 140 °C, 36 h.

b Yields are isolated pure products 3aa–3cg.

c Determined by NMR analysis.

Furthermore, to establish the usefulness of the strategy, this time, the reaction was tested with the aromatic variant of *ortho*-bromo tertiary benzylic alcohols 1d–1g with internal alkynes 2a–2f. In general, the reaction exhibited a broad substrate scope and delivered the products 3da–3ga (Table 3). The protocol was tolerable to different functionalities of either aromatic rings. Notably, the strategy was found to be smooth with electron-donating OMe groups of *ortho*-bromo tertiary benzylic alcohols 1d–1f and also electron-withdrawing Cl group of *ortho*-bromo tertiary benzylic alcohol 1g. On the other hand, amenable to simple phenyl, tolyl, anisyl, and thiophenyl rings of internal alkynes as well.

**Table 3:** Scope to yield isochromenes 3da–3ga from *ortho*-bromo tertiary benzylic alcohols 1d–1g and acetylenes 2a–2f^a^^,^^b^

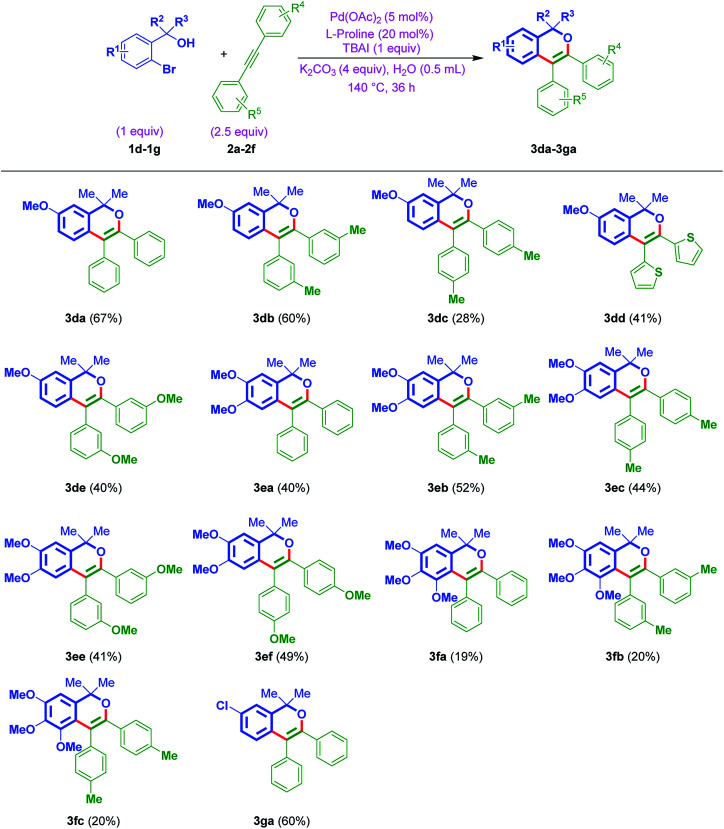

a Conditions: 1d–1g (0.25 mmol, 1 equiv.), 2a–2f (0.625 mmol, 2.5 equiv.), Pd(OAc)_2_ (2.8 mg, 0.012 mmol, 5 mol%), K_2_CO_3_ (138.6 mg, 1 mmol, 4 equiv.), l-proline (5.6 mg, 0.05 mol, 20 mol%), TBAI (tetrabutylammonium iodide) (92.3 mg, 0.25 mmol, 1 equiv.), 140 °C, 36 h.

b Yields are isolated pure products 3da–3ga.

Furthermore, to show the effectiveness of the protocol, the annulation of *ortho*-bromo secondary benzylic alcohol 1h was attempted with internal alkynes (2a & 2h), using optimized conditions. Notably, as anticipated, afforded the cyclic ethers 3ha and 3hh in 41% and 44% yields, respectively (Table 4).

**Table 4:** Scope with secondary benzylic alcohol 1h^a^^,^^b^

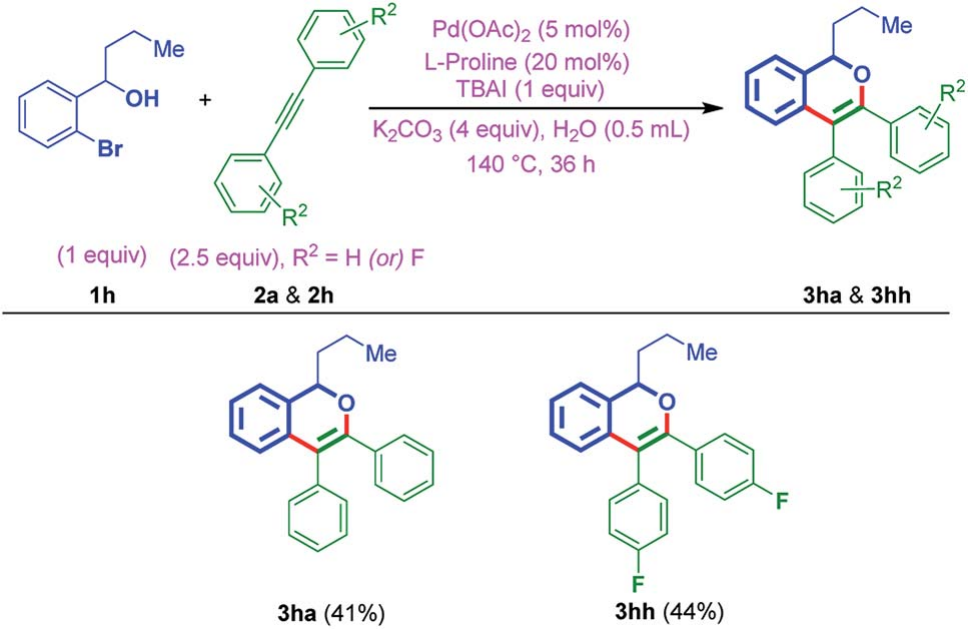

a Conditions: 1h (57 mg, 0.25 mmol, 1 equiv.), 2a or 2h (0.625 mmol, 2.5 equiv.), Pd(OAc)_2_ (2.8 mg, 0.012 mmol, 5 mol%), K_2_CO_3_ (138.6 mg, 1 mmol, 4 equiv.), l-proline (5.6 mg, 0.05 mol, 20 mol%), TBAI (92.3 mg, 0.25 mmol, 1 equiv.), 140 °C, 36 h.

b Yields are isolated pure products 3ha & 3hh.

**Scheme 1:**
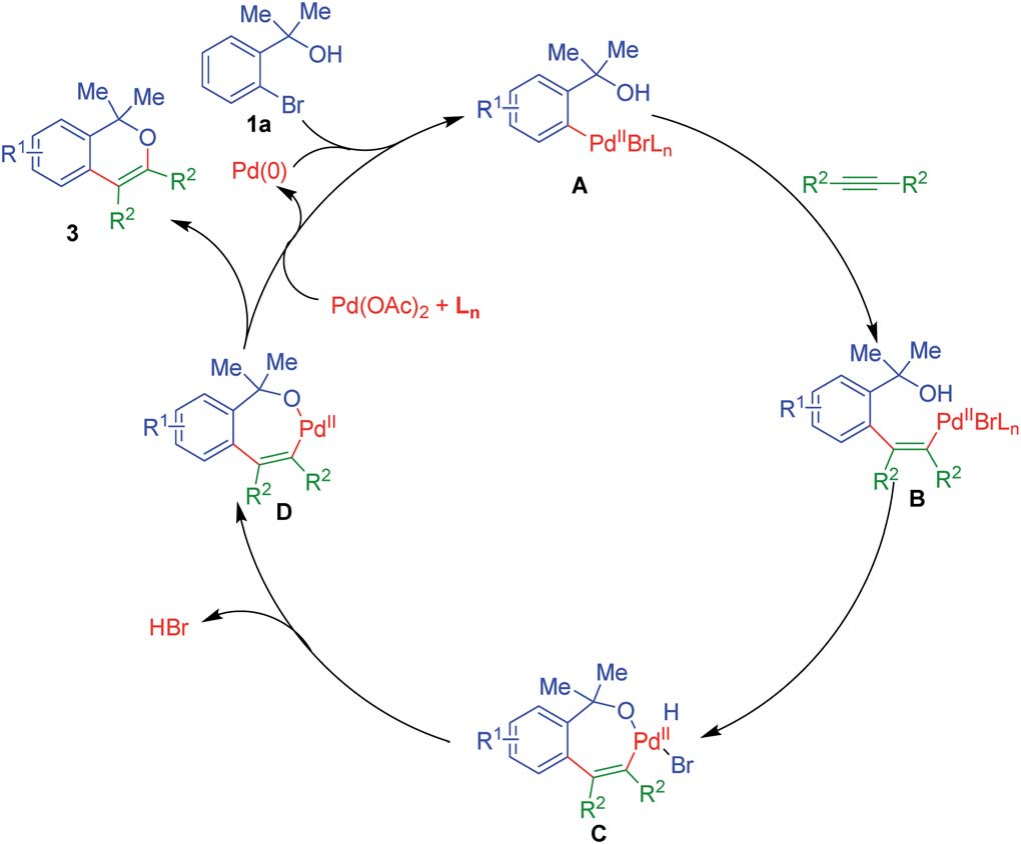
Plausible mechanism for the formation of isochromenes 3.

A plausible mechanistic path of this annulation process is as depicted in Scheme 1.

Initially, Pd(ii) species A could be formed *via* oxidative incorporation of active Pd(0) catalyst onto the sigma C–Br bond of *ortho*-bromo tertiary benzylic alcohols 1. The subsequent, *syn*-addition reaction of A upon C≡C bond of alkynes 2, gives acyclic Pd(ii)-species B, which on subsequent intramolecular coupling with the nucleophilic hydroxyl group, would furnish a seven-membered palladacycle C [Pd(ii)]. Removal of HBr from C could yield seven-membered Pd(ii)-cycle D. Ultimately, reductive elimination of D, gives isochromenes 3 along with the regeneration of Pd(0) active catalyst and hence, fulfils the overall catalytic path of the reaction.^19^

## Conclusions

In summary, a palladium-catalyst promoted annulation between *ortho*-bromo benzylic alcohols and internal acetylenes is established, for achieving isochromenes. Significantly, this dual bond-forming process was feasible in water solvent and enabled the formation of various isochoromenes.

## Experimental section

### General

IR spectra were recorded on a Bruker Tensor 37 (FTIR) spectrophotometer. ^1^H NMR spectra were recorded on Bruker Avance 400 (400 MHz) spectrometer at 295 K in CDCl_3_; chemical shifts (*δ* ppm) and coupling constants (Hz) are reported in standard fashion with reference to either internal standard tetramethylsilane (TMS) (*δ*_H_ = 0.00 ppm) or CDCl_3_ (*δ*_H_ = 7.25 ppm). ^13^C{^1^H} NMR spectra were recorded on Bruker Avance 400 (100 MHz) spectrometer at RT in CDCl_3_; chemical shifts (*δ* ppm) are reported relative to CDCl_3_ [*δ*_C_ = 77.00 ppm (central line of triplet)]. In the ^13^C{^1^H} NMR, the nature of carbons (C, CH, CH_2_ and CH_3_) was determined by recording the DEPT-135 spectra, and is given in parentheses and noted as s = singlet (for C), d = doublet (for CH), t = triplet (for CH_2_) and q = quartet (for CH_3_). In the ^1^H-NMR, the following abbreviations were used throughout: s = singlet, d = doublet, t = triplet, q = quartet, qui = quintet, sept = septet, dd = doublet of doublets, m = multiplet and br. s = broad singlet. The assignment of signals was confirmed by ^1^H, ^13^C{^1^H} CPD and DEPT spectra. High-resolution mass spectra (HR-MS) were recorded on an Agilent 6538 UHD Q-TOF electron spray ionization (ESI) mode and atmospheric pressure chemical ionization (APCI) modes. Solvents were distilled prior to use; petroleum ether with a boiling range of 60 to 80 °C was used. Palladium acetate, l-proline, TBAI (tetrabutylammonium iodide) and K_2_CO_3_ were purchased from Sigma-Aldrich/local sources and used as received. Acme's silica gel (60–120 mesh) was used for column chromatography (approximately 20 g per one gram of crude material). It is worth noting that these sort of experimental procedures have already been published elsewhere.^17^

### GP (general procedure for the synthesis of isochromenes)

An oven dried Screwcap vial was equipped with a magnetic stir bar and Pd(OAc)_2_ (2.8 mg, 5 mol%), l-proline (5.6 mg, 20 mol%), TBAI (92.3 mg, 0.25 mmol), K_2_CO_3_ (138.6 mg, 1 mmol) and water (0.3 mL) were added *via* syringe. After the addition of solvent, the solution was heated at 140 °C for 10 min for pre catalyst formation and brought to ambient temperature. A second oven dried vial was equipped with the starting materials, *ortho*-bromobenzyl alcohols 1 (54.0–84.8 mg, 0.25 mmol), internal alkynes 2 (111.2–196.2 mg, 0.625 mmol) and water (0.2 mL) was added at room temperature. This solution was then transferred to the first vial in which active catalyst was there *via* syringe. The reaction mixture was stirred at 140 °C for 36 h. Reaction mixture was then cooled to room temperature, quenched by the addition of aqueous NaHCO_3_ solution and extracted by using ethyl acetate (3 × 20 mL). The organic layer was washed with saturated NH_4_Cl solution, dried by Na_2_SO_4_ and then filtered. Evaporation of the solvent(s) under reduced pressure and refinement of the crude mixture by silica gel column chromatography (petroleum ether/ethyl acetate) gave the isochromenes 3 (19–82%) as semi-solid/solid.

### 1,1-Dimethyl-3,4-bis(3-methylphenyl)-1*H*-isochromene (3ab)

GP was carried out with 2-(2-bromophenyl)propan-2-ol 1a (53.4 mg, 0.25 mmol), 1-methyl-3-[(3-methylphenyl)ethynyl]benzene 2b (128.8 mg, 0.625 mmol), palladium acetate (2.8 mg, 5 mol%), l-proline (5.6 mg, 20 mol%), TBAI (92.3 mg, 0.25 mmol), K_2_CO_3_ (138.6 mg, 1 mmol) and water (0.5 mL). The crude material was then refined by silica gel column chromatography (petroleum ether/ethyl acetate, 100 : 0 to 99 : 1) to obtain isochromene 3ab (69.6 mg, 82%) as a light yellow solid compound, melting point = 140 °C, [TLC control (petroleum ether/ethyl acetate 99 : 1), *R*_f_ (2b) = 0.8, *R*_f_ (3ab) = 0.7, UV detection]. IR (MIR-ATR, 4000–600 cm^−1^): *ν*_max_ = 3057, 3018, 1688, 1471, 1355, 1249, 1040, 956, 744, 598 cm^−1^. ^1^H NMR (CDCl_3_, 400 MHz): *δ* = 7.26–7.17 (m, 3H, Ar-H), 7.16–7.08 (m, 3H, Ar-H), 7.07–6.99 (m, 4H, Ar-H), 6.96 (s, 1H, Ar-H), 6.88 (d, 1H, *J* = 7.8 Hz, Ar-H), 2.31 (s, 3H, CH_3_), 2.20 (s, 3H, CH_3_), 1.78 (s, 6H, 2 × CH_3_) ppm. ^13^C{^1^H} NMR (CDCl_3_, 100 MHz): *δ* = 147.9 (s, Ar-C), 138.0 (s, Ar-C), 137.0 (s, Ar-C), 136.7 (s, Ar-C), 136.4 (s, Ar-C), 135.9 (s, Ar-C), 132.2 (d, 2 × Ar-CH), 129.3 (d, Ar-CH), 128.7 (d, Ar-CH), 128.5 (d, Ar-CH), 128.3 (d, Ar-CH), 127.6 (d, Ar-CH), 127.2 (d, Ar-CH), 127.2 (d, Ar-CH), 126.7 (d, Ar-CH), 126.0 (d, Ar-CH), 123.6 (d, Ar-CH), 122.1 (d, Ar-CH), 115.7 (s, Ar-CH), 77.5 (s, Ar-CH), 27.0 (q, 2 × CH_3_), 21.4 (q, CH_3_), 21.4 (q, CH_3_) ppm. HR-MS (ESI+) *m*/*z* calculated for [C_25_H_25_O]^+^ = [M + H]^+^: 341.1900; found 341.1901.

### 1,1-Dimethyl-3,4-dithien-2-yl-1*H*-isochromene (3ad)

GP was carried out with 2-(2-bromophenyl)propan-2-ol 1a (53.4 mg, 0.25 mmol), 2-(thien-2-ylethynyl)thiophene 2d (118.6 mg, 0.625 mmol), palladium acetate (2.8 mg, 5 mol%), l-proline (5.6 mg, 20 mol%), TBAI (92.3 mg, 0.25 mmol), K_2_CO_3_ (138.6 mg, 1 mmol) and water (0.5 mL). The crude material was then refined by silica gel column chromatography (petroleum ether/ethyl acetate, 100 : 0 to 99 : 1) to obtain isochromene 3ad (32.3 mg, 40%) as a light yellow jelly compound, [TLC control (petroleum ether/ethyl acetate 99 : 1), *R*_f_ (2d) = 0.8, *R*_f_ (3ad) = 0.7, UV detection]. IR (MIR-ATR, 4000–600 cm^−1^): *ν*_max_ = 2981, 1714, 1589, 1445, 1238, 1158, 1093, 1017, 922, 750, 680 cm^−1^. ^1^H NMR (CDCl_3_, 400 MHz): *δ* = 7.52 (dd, 1H, *J* = 5.4 and 1.0 Hz, Ar-H), 7.24–7.13 (m, 5H, Ar-H), 7.05 (dd, 1H, *J* = 5.4 and 1.0 Hz, Ar-H), 7.00–6.92 (m, 2H, Ar-H), 6.91–6.83 (m, 1H, Ar-H), 1.75 (s, 6H, 2 × CH_3_) ppm. ^13^C{^1^H} NMR (CDCl_3_, 100 MHz): *δ* = 145.5 (s, Ar-C) 138.5 (s, Ar-C) 137.1 (s, Ar-C), 136.0 (s, Ar-C), 132.1 (s, Ar-C), 129.8 (d, Ar-CH), 127.8 (d, Ar-CH), 127.7 (d, Ar-CH), 127.6 (d, Ar-CH), 127.5 (d, Ar-CH) 127.5 (d, Ar-CH), 126.9 (d, Ar-CH), 126.8 (d, Ar-CH), 123.6 (d, Ar-CH), 122.0 (d, Ar-CH), 106.2 (s, Ar-CH), 78.5 (s, Ar-CH), 27.0 (q, 2 × CH_3_) ppm. HR-MS (ESI+) *m*/*z* calculated for [C_19_H_17_OS_2_]^+^ = [M + H]^+^: 325.0715; found 325.0705.

### 3,4-Bis(3-methoxyphenyl)-1,1-dimethyl-1*H*-isochromene (3ae)

GP was carried out with 2-(2-bromophenyl)propan-2-ol 1a (53.4 mg, 0.25 mmol), 1-methoxy-3-[(3-methoxyphenyl)ethynyl]benzene 2e (148.7 mg, 0.625 mmol), palladium acetate (2.8 mg, 5 mol%), l-proline (5.6 mg, 20 mol%), TBAI (92.3 mg, 0.25 mmol), K_2_CO_3_ (138.6 mg, 1 mmol) and water (0.5 mL). The crude material was then refined by silica gel column chromatography (petroleum ether/ethyl acetate, 99 : 1 to 98 : 2) to obtain isochromene 3ae (51 mg, 55%) as a light yellow solid compound, melting point = 82 °C, [TLC control (petroleum ether/ethyl acetate 98 : 2), *R*_f_ (2e) = 0.6, *R*_f_ (3ae) = 0.5, UV detection]. IR (MIR-ATR, 4000–600 cm^−1^): *ν*_max_ = 2839, 1596, 1511, 1458, 1249, 1140, 1027, 810, 761 cm^−1^. ^1^H NMR (CDCl_3_, 400 MHz): *δ* = 7.26 (ddd, *J* = 7.8, 78 and 1.0 Hz, 1H, Ar-H), 7.23–7.18 (m, 2H, Ar-H), 7.14–7.10 (m, 1H, Ar-H), 7.06 (ddd, *J* = 7.8, 78 and 1.0 Hz, 1H, Ar-H), 6.93 (ddd, *J* = 7.8, 78 and 1.0 Hz, 2H, Ar-H), 6.89–6.77 (m, 4H, Ar-H), 6.76–6.69 (m, 1H, Ar-H), 3.72 (s, 3H, OCH_3_), 3.55 (s, 3H, OCH_3_), 1.77 (s, 6H, 2 × CH_3_) ppm. ^13^C{^1^H} NMR (CDCl_3_, 100 MHz): *δ* = 159.8 (s, Ar-C), 158.6 (s, Ar-C), 147.6 (s, Ar-C), 138.5 (s, Ar-C), 137.2 (s, Ar-C), 136.3 (s, Ar-C), 131.8 (s, Ar-C), 129.6 (d, Ar-CH), 128.5 (d, Ar-CH), 127.2 (d, Ar-CH), 126.9 (d, Ar-CH), 124.1 (d, Ar-CH), 123.7 (d, Ar-CH), 122.1 (d, Ar-CH), 121.1 (d, Ar-CH), 116.9 (d, Ar-CH), 115.6 (s, Ar-CH), 114.2 (d, Ar-CH), 113.7 (d, Ar-CH), 112.6 (d, Ar-CH), 77.6 (s, Ar-C), 55.2 (q, OCH_3_), 54.9 (q, OCH_3_), 27.0 (q, 2 × CH_3_) ppm. HR-MS (APCI+) *m*/*z* calculated for [C_25_H_25_O_3_]^+^ = [M + H]^+^: 373.1798; found 373.1784.

### 1,1-Dimethyl-3,4-bis[4-(trifluoromethyl)phenyl]-1*H*-isochromene (3ai)

GP was carried out with 2-(2-bromophenyl)propan-2-ol 1a (53.4 mg, 0.25 mmol), 1-(trifluoromethyl)-4-{[4-(trifluoromethyl)phenyl]ethynyl}benzene 2i (196.2 mg, 0.625 mmol), palladium acetate (2.8 mg, 5 mol%), l-proline (5.6 mg, 20 mol%), TBAI (92.3 mg, 0.25 mmol), K_2_CO_3_ (138.6 mg, 1 mmol) and water (0.5 mL). The crude material was then refined by silica gel column chromatography (petroleum ether/ethyl acetate, 99 : 1 to 98 : 2) to obtain isochromene 3ai (84.9 mg, 76%) as a light yellow jelly compound, [TLC control (petroleum ether/ethyl acetate 98 : 2), *R*_f_ (2i) = 0.7, *R*_f_ (3ai) = 0.6, UV detection]. IR (MIR-ATR, 4000–600 cm^−1^): *ν*_max_ = 2922, 2854, 1619, 1444, 1329, 1245, 1166, 1124, 1073, 914, 803, 753, 700 cm^−1^. ^1^H NMR (CDCl_3_, 400 MHz): *δ* = 7.65–7.55 (m, 1H, Ar-H), 7.55–7.34 (m, 6H, Ar-H), 7.33–7.21 (m, 3H, Ar-H), 7.21–7.07 (m, 1H, Ar-H), 6.91–6.69 (m, 1H, Ar-H), 1.80 (s, 6H, 2 × CH_3_) ppm. ^13^C{^1^H} NMR (CDCl_3_, 100 MHz): *δ* = 147.5 (s, Ar-C), 137.5 (s, 1 Ar-C), 136.3 (s, Ar-C), 136.2 (s, Ar-C), 135.0 (d, Ar-CH), 131.7 (d, Ar-CH), 131.3 (d, *J* = 32.3 Hz, Ar-C), 130.8 (d, Ar-CH), 130.1 (d, *J* = 5.1 Hz, Ar-C), 129.3 (d, Ar-CH), 128.3 (q, *J*_c–f_ = 3.7 Hz, Ar-C), 128.1 (d, Ar-CH), 127.7 (d, Ar-CH), 127.5 (d, Ar-CH), 125.6 (q, *J*_c–f_ = 3.7 Hz, Ar-C), 124.6 (q, *J*_c–f_ = 3.7 Hz, Ar-C), 124.2 (q, *J*_c–f_ = 3.7 Hz, Ar-C), 124.0 (q, *J*_c–f_ = 272.9 Hz, Ar-CF_3_), 123.8 (q, *J*_c–f_ = 272.9 Hz, Ar-CF_3_), 123.4 (d, Ar-CH), 122.6 (d, Ar-CH), 115.5 (s, Ar-C), 78.4 (s, Ar-C), 27.2 (q, 2 × CH_3_) ppm. HR-MS (APCI+) *m*/*z* calculated for [C_25_H_19_F_6_O]^+^ = [M + H]^+^: 449.1335; found 449.1338.

### 3-(3-Methoxyphenyl)-1,1-dimethyl-4-phenyl-1*H*-isochromene (3aj) and 4-(3-methoxyphenyl)-1,1-dimethyl-3-phenyl-1*H*-isochromene (3aj′)

GP was carried out with 2-(2-bromophenyl)propan-2-ol 1a (53.4 mg, 0.25 mmol), 1-methoxy-3-(phenylethynyl)benzene 2j (130 mg, 0.625 mmol), palladium acetate (2.8 mg, 5 mol%), l-proline (5.6 mg, 20 mol%), TBAI (92.3 mg, 0.25 mmol), K_2_CO_3_ (138.6 mg, 1 mmol) and water (0.5 mL). The crude material was then refined by silica gel column chromatography (petroleum ether/ethyl acetate, 100 : 0 to 99 : 1) to obtain isochromene 3aj + 3aj′ (29.8 mg, 35%) as a light yellow jelly compound, [TLC control (petroleum ether/ethyl acetate 100 : 0), *R*_f_ (2j) = 0.7, *R*_f_ (3aj + 3aj′) = 0.6, UV detection]. IR (MIR-ATR, 4000–600 cm^−1^): *ν*_max_ = 2975, 2927, 1695, 1591, 1481, 1253, 1158, 1044, 761, 700 cm^−1^. ^1^H NMR (CDCl_3_, 400 MHz): *δ* = 7.41–7.33 (m, 2H, Ar-H), 7.33–7.22 (m, 6H, Ar-H), 7.22–7.18 (m, 4H, Ar-H), 7.18–7.10 (m, 5H, Ar-H), 7.06 (ddd, *J* = 7.8, 7.8 and 1.0 Hz, 1H, Ar-H), 6.97–6.87 (m, 3H, Ar-H), 6.86–6.81 (m, 2H, Ar-H), 6.79–6.76 (m, 1H, Ar-H), 6.75–6.72 (m, 1H, Ar-H), 6.72–6.66 (m, 1H, Ar-H), 3.73 (s, 3H, OCH_3_), 3.53 (s, 3H, OCH_3_), 1.79 (s, 12H, 4 × CH_3_) ppm. ^13^C{^1^H} NMR (CDCl_3_, 100 MHz): *δ* = 159.7 (s, Ar-C), 158.5 (s, Ar-C), 148.0 (s, Ar-C), 147.7 (s, Ar-C), 138.4 (s, Ar-C), 137.2 (s, Ar-C), 136.3 (s, Ar-C), 136.2 (s, Ar-C), 136.0 (s, Ar-C), 131.9 (s, Ar-C), 131.8 (s, Ar-C), 131.6 (d, 3 × Ar-CH), 129.5 (d, Ar-CH), 128.6 (d, 4 × Ar-CH), 128.4 (d, Ar-CH), 127.8 (d, Ar-CH), 127.5 (d, 3 × Ar-CH), 127.2 (d, Ar-CH), 127.2 (d, Ar-CH), 126.9 (d, Ar-CH), 126.9 (d, Ar-CH), 126.9 (d, Ar-CH), 124.2 (d, Ar-CH), 123.6 (d, Ar-CH), 123.6 (d, Ar-CH), 122.2 (d, 2 × Ar-CH), 121.2 (d, Ar-CH), 117.0 (d, Ar-CH), 115.8 (s, Ar-C), 115.5 (s, Ar-C), 114.2 (d, Ar-CH), 113.9 (d, Ar-CH), 112.6 (d, Ar-CH), 77.7 (s, Ar-C), 55.2 (q, OCH_3_), 54.9 (q, OCH_3_), 27.08 (q, 2 × CH_3_) 27.05 (q, 2 × CH_3_) ppm. HR-MS (APCI+) *m*/*z* calculated for [C_24_H_23_O_2_]^+^ = [M + H]^+^: 343.1693; found 343.1693.

### 3-(4-Fluorophenyl)-1,1-dimethyl-4-phenyl-1*H*-isochromene (3ak) and 4-(4-fluorophenyl)-1,1-dimethyl-3-phenyl-1*H*-isochromene (3ak′)

GP was carried out with 2-(2-bromophenyl)propan-2-ol 1a (53.4 mg, 0.25 mmol), 1-fluoro-4-(phenylethynyl)benzene 2k (122.5 mg, 0.625 mmol), palladium acetate (2.8 mg, 5 mol%), l-proline (5.6 mg, 20 mol%), TBAI (92.3 mg, 0.25 mmol), K_2_CO_3_ (138.6 mg, 1 mmol) and water (0.5 mL). The crude material was then refined by silica gel column chromatography (petroleum ether/ethyl acetate, 99 : 1 to 98 : 2) to obtain isochromene 3ak + 3ak′ (30.4 mg, 37%) as a light yellow jelly compound, [TLC control (petroleum ether/ethyl acetate 98 : 2), *R*_f_ (2k) = 0.8, *R*_f_ (3ak + 3ak′) = 0.7, UV detection]. IR (MIR-ATR, 4000–600 cm^−1^): *ν*_max_ = 3062, 2978, 2925, 1695, 1610, 1507, 1230, 1155, 1099, 962, 834, 760, 700 cm^−1^. ^1^H NMR (CDCl_3_, 400 MHz): *δ* = 7.42–7.28 (m, 4H, Ar-H), 7.28–7.18 (m, 12H, Ar-H), 7.17–7.10 (m, 4H, Ar-H), 7.06–7.00 (m, 2H, Ar-H), 6.93–6.75 (m, 4H, Ar-H), 1.79 (s, 12H, 4 × CH_3_) ppm. ^13^C{^1^H} NMR (CDCl_3_, 100 MHz): *δ* = 162.0 (d, *J* = 245.0 Hz, Ar-C), 160.8 (s, Ar-C), 147.1 (s, Ar-C), 136.9 (s, Ar-C), 136.2 (s, Ar-C), 135.9 (s, Ar-C), 133.3 (d, Ar-CH), 133.2 (d, Ar-CH), 131.8 (s, Ar-CH), 131.6 (d, 4 × Ar-CH), 130.7 (d, Ar-CH), 130.6 (d, Ar-CH), 128.7 (d, 3 × Ar-CH), 128.6 (d, 5 × Ar-CH), 127.9 (d, Ar-CH), 127.5 (d, 3 × Ar-CH), 127.3 (d, Ar-CH), 127.0 (d, 3 × Ar-CH), 127.0 (d, Ar-CH), 126.9 (d, Ar-CH), 123.5 (d, Ar-CH), 123.3 (d, Ar-CH), 122.3 (d, Ar-CH), 122.2 (d, Ar-CH), 115.7 (d, Ar-CH), 115.4 (d, Ar-CH), 114.5 (d, Ar-CH), 114.3 (d, Ar-CH), 77.8 (s, Ar-C), 27.1 (q, 4 × CH_3_) ppm. HR-MS (APCI+) *m*/*z* calculated for [C_23_H_20_FO]^+^ = [M + H]^+^: 331.1493; found 331.1494.

### 1-Methyl-3,4-diphenyl-1-propyl-1*H*-isochromene (3ba)

GP was carried out with 2-(2-bromophenyl)pentan-2-ol 1b (60.5 mg), (phenylethynyl)benzene 2a (111.2 mg, 0.625 mmol), palladium acetate (2.8 mg, 5 mol%), l-proline (5.6 mg, 20 mol%), TBAI (92.3 mg, 0.25 mmol), K_2_CO_3_ (138.6 mg, 1 mmol) and water (0.5 mL). The crude material was then refined by silica gel column chromatography (petroleum ether/ethyl acetate, 100 : 0 to 99 : 1) to obtain isochromene 3ba (36.5 mg, 43%) as a light yellow jelly compound, [TLC control (petroleum ether/ethyl acetate 100 : 0), *R*_f_ (2a) = 0.8, *R*_f_ (3ba) = 0.7, UV detection]. IR (MIR-ATR, 4000–600 cm^−1^): *ν*_max_ = 3055, 2947, 1702, 1605, 1477, 1445, 1348, 1278, 1231, 1026, 703 cm^−1^. ^1^H NMR (CDCl_3_, 400 MHz): *δ* = 7.37–7.27 (m, 3H, Ar-H), 7.27–7.19 (m, 4H, Ar-H), 7.19–7.05 (m, 6H, Ar-H), 6.85 (dd, *J* = 7.3 and 1.0 Hz, 1H, Ar-H), 2.22–1.99 (m, 2H, CH_2_), 1.74 (s, 3H, CH_3_), 1.58–1.39 (m, 2H, CH_2_), 0.93 (t, *J* = 7.3 Hz, 3H, CH_3_) ppm. ^13^C{^1^H} NMR (CDCl_3_, 100 MHz): *δ* = 148.0 (s, Ar-C), 137.2 (s, Ar-C), 136.1 (s, Ar-C), 135.3 (s, Ar-C), 132.4 (s, Ar-C), 131.7 (d, 2 × Ar-CH), 128.7 (d, 3 × Ar-CH), 128.5 (d, Ar-CH), 127.6 (d, Ar-CH), 127.4 (d, 2 × Ar-CH), 127.0 (d, Ar-CH), 126.9 (d, Ar-CH), 126.5 (d, Ar-CH), 123.5 (d, Ar-CH), 122.9 (d, Ar-CH), 115.1 (s, Ar-C), 80.1 (s, Ar-C), 41.8 (t, CH_2_), 25.7 (q, CH_3_), 17.4 (t, CH_2_), 14.6 (q, CH_3_) ppm. HR-MS (APCI+) *m*/*z* calculated for [C_25_H_25_O]^+^ = [M + H]^+^: 341.1900; found 341.1906.

### 1-Methyl-3,4-bis(4-methylphenyl)-1-propyl-1*H*-isochromene (3bc)

GP was carried out with 2-(2-bromophenyl)pentan-2-ol 1b (60.5 mg, 0.25 mmol), 1-methyl-4-[(4-methylphenyl)ethynyl]benzene 2c (128.8 mg, 0.625 mmol), palladium acetate (2.8 mg, 5 mol%), l-proline (5.6 mg, 20 mol%), TBAI (92.3 mg, 0.25 mmol), K_2_CO_3_ (138.6 mg, 1 mmol) and water (0.5 mL). The crude material was then refined by silica gel column chromatography (petroleum ether/ethyl acetate, 100 : 0 to 99 : 1) to obtain isochromene 3bc (18.4 mg, 20%) as a brown jelly compound, [TLC control (petroleum ether/ethyl acetate 99 : 1), *R*_f_ (2c) = 0.7, *R*_f_ (3bc) = 0.6, UV detection]. IR (MIR-ATR, 4000–600 cm^−1^): *ν*_max_ = 3025, 2920, 1670, 1507, 1446, 1261, 1181, 1108, 1033, 946, 819, 732 cm^−1^. ^1^H NMR (CDCl_3_, 400 MHz): *δ* = 7.20–6.99 (m, 8H, Ar-H), 6.94 (d, *J* = 1.0 Hz, 1H, Ar-H), 6.93(dd, *J* = 7.8 and 1.0 Hz, 1H, Ar-H), 6.84 (dd, *J* = 7.8 and 1.0 Hz, 1H, Ar-H), 6.64 (d, *J* = 2.0 Hz, 1H, Ar-H), 2.36 (s, 3H, CH_3_), 2.24 (s, 3H, CH_3_), 2.15–1.87 (m, 2H, CH_2_), 1.54 (s, 3H, CH_3_), 1.52–1.36 (m, 2H, CH_2_), 0.91 (t, *J* = 7.3 Hz, 3H, CH_3_) ppm. ^13^C{^1^H} NMR (CDCl_3_, 100 MHz): *δ* = 147.9 (s, Ar-C), 137.4 (s, Ar-C), 136.3 (s, Ar-C), 135.3 (s, Ar-C), 134.2 (s, Ar-C), 133.3 (s, Ar-C), 132.7 (s, Ar-C), 131.5 (d, 2 × Ar-CH), 131.3 (d, Ar-CH) 129.3 (d, 2 × Ar-CH), 128.6 (d, 2 × Ar-CH), 128.1 (d, 2 × Ar-CH), 127.0 (d, Ar-CH), 126.3 (d, Ar-CH), 123.5 (d, Ar-CH), 122.8 (s, Ar-C), 79.8 (s, Ar-C), 41.8 (t, CH_2_), 25.6 (q, CH_3_), 21.3 (q, CH_3_), 21.2 (q, CH_3_), 17.4 (t, CH_2_), 14.6 (q, CH_3_) ppm. HR-MS (APCI+) *m*/*z* calculated for [C_27_H_29_O]^+^ = [M + H]^+^: 369.2213; found 369.2218.

### 3,4-Bis(3,4-dimethoxyphenyl)-1,1-diphenyl-1*H*-isochromene (3cg)

GP was carried out with (2-bromophenyl)(diphenyl)methanol 1c (84.5 mg, 0.25 mmol), 4-[(3,4-dimethoxyphenyl)ethynyl]-1,2-dimethoxybenzene 2g (186.3 mg, 0.625 mmol), palladium acetate (2.8 mg, 5 mol%), l-proline (5.6 mg, 20 mol%), TBAI (92.3 mg, 0.25 mmol), K_2_CO_3_ (138.6 mg, 1 mmol) and water (0.5 mL). The crude material was then refined by silica gel column chromatography (petroleum ether/ethyl acetate, 73 : 27 to 70 : 30) to obtain isochromene 3cg (34.7 mg, 25%) as a light yellow jelly compound, [TLC control (petroleum ether/ethyl acetate 70 : 30), *R*_f_ (2g) = 0.5, *R*_f_ (3cg) = 0.4, UV detection]. IR (MIR-ATR, 4000–600 cm^−1^): *ν*_max_ = 2925, 2850, 1511, 1459, 1254, 1151, 1028, 763, 702 cm^−1^. ^1^H NMR (CDCl_3_, 400 MHz): *δ* = 7.37–7.26 (m, 10H, Ar-H), 7.23 (ddd, *J* = 7.8, 7.8 and 1.5 Hz, 1H, Ar-H), 7.11 (ddd, *J* = 7.8, 7.8 and 1.5 Hz, 1H, Ar-H), 7.08–6.99 (m, 2H, Ar-H), 6.81 (d, *J* = 8.3 Hz, 1H, Ar-H), 6.74–6.62 (m, 3H, Ar-H) 6.56 (dd, *J* = 8.3 and 2.0 Hz, 1H, Ar-H), 6.50 (d, *J* = 2.0 Hz, 1H, Ar-H), 3.85 (s, 3H, OCH_3_), 3.80 (s, 3H, OCH_3_), 3.68 (s, 3H, OCH_3_), 3.48 (s, 3H, OCH_3_) ppm. ^13^C{^1^H} NMR (CDCl_3_, 100 MHz): *δ* = 149.1 (s, Ar-C), 148.5 (s, Ar-C), 148.0 (s, Ar-C), 147.9 (s, Ar-C), 147.4 (s, Ar-C), 144.0 (d, Ar-CH), 134.2 (s, Ar-C), 133.5 (s, Ar-C), 131.5 (s, Ar-C), 130.0 (s, Ar-C), 129.8 (s, Ar-C), 128.8 (d, 3 × Ar-CH), 128.2 (s, Ar-CH), 127.9 (d, Ar-CH), 127.7 (d, 2 × Ar-CH), 127.5 (d, 4 × Ar-CH), 126.8 (s, Ar-C), 126.0 (d, Ar-CH), 123.6 (d, Ar-CH), 123.1 (d, Ar-CH), 121.3 (d, Ar-CH), 116.5 (s, Ar-C), 114.5 (d, Ar-CH), 111.9 (d, Ar-CH), 111.4 (d, Ar-CH), 110.0 (d, Ar-CH), 86.7 (s, Ar-C), 55.9 (q, OCH_3_), 55.8 (q, OCH_3_), 55.6 (q, OCH_3_), 55.3 (q, OCH_3_) ppm. HR-MS (APCI+) *m*/*z* calculated for [C_37_H_33_O_5_]^+^ = [M + H]^+^: 557.2323; found 557.2331.

### 7-Methoxy-1,1-dimethyl-3,4-diphenyl-1*H*-isochromene (3da)

GP was carried out with 2-(2-bromo-5-methoxyphenyl)propan-2-ol 1d (61 mg, 0.25 mmol), (phenylethynyl)benzene 2a (111.2 mg, 0.625 mmol), palladium acetate (2.8 mg, 5 mol%), l-proline (5.6 mg, 20 mol%), TBAI (92.3 mg, 0.25 mmol), K_2_CO_3_ (138.6 mg, 1 mmol) and water (0.5 mL). The crude material was then refined by silica gel column chromatography (petroleum ether/ethyl acetate, 100 : 0 to 99 : 1) to obtain isochromene 3da (57.2 mg, 67%) as a brown jelly compound, [TLC control (petroleum ether/ethyl acetate 100 : 0), *R*_f_ (2a) = 0.8, *R*_f_ (3da) = 0.7, UV detection]. IR (MIR-ATR, 4000–600 cm^−1^): *ν*_max_ = 2925, 2364, 1743, 1694, 1511, 1301, 1069, 758, 699 cm^−1^. ^1^H NMR (CDCl_3_, 400 MHz): *δ* = 7.39–7.28 (m, 2H, Ar-H), 7.28–7.19 (m, 4H, Ar-H), 7.17–7.08 (m, 3H, Ar-H), 6.89–6.75 (m, 3H, Ar-H), 6.67 (dd, *J* = 8.6 and 2.7 Hz, 1H, Ar-H), 3.81 (s, 3H, OCH_3_), 1.77 (s, 6H, 2 × CH_3_) ppm. ^13^C{^1^H} NMR (CDCl_3_, 100 MHz): *δ* = 158.8 (s, Ar-C), 146.0 (s, Ar-C), 138.3 (s, Ar-C), 137.3 (s, Ar-C), 136.1 (s, Ar-C), 131.6 (d, 2 × Ar-CH), 131.4 (d, Ar-CH), 128.6 (d, Ar-CH), 128.5 (d, 2 × Ar-CH), 127.4 (d, Ar-CH), 127.4 (d, Ar-CH), 126.9 (d, Ar-CH), 126.5 (d, Ar-CH), 125.3 (s, Ar-C), 125.1 (d, Ar-CH), 115.6 (s, Ar-C), 111.3 (d, Ar-CH), 109.0 (d, Ar-CH), 77.4 (s, Ar-C), 55.4 (q, OCH_3_), 26.9 (q, 2 × CH_3_) ppm. HR-MS (APCI+) *m*/*z* calculated for [C_24_H_23_O_2_]^+^ = [M + H]^+^: 343.1693; found 343.1686.

### 7-Methoxy-1,1-dimethyl-3,4-bis(3-methylphenyl)-1*H*-isochromene (3db)

GP was carried out with 2-(2-bromo-5-methoxyphenyl)propan-2-ol 1d (61 mg, 0.25 mmol), 1-methyl-3-[(3-methylphenyl)ethynyl]benzene 2b (128.8 mg, 0.625 mmol), palladium acetate (2.8 mg, 5 mol%), l-proline (5.6 mg, 20 mol%), TBAI (92.3 mg, 0.25 mmol), K_2_CO_3_ (138.6 mg, 1 mmol) and water (0.5 mL). The crude material was then refined by silica gel column chromatography (petroleum ether/ethyl acetate, 99 : 1 to 98 : 2) to obtain isochromene 3db (55.5 mg, 60%) as a brown jelly compound, [TLC control (petroleum ether/ethyl acetate 98 : 2), *R*_f_ (2b) = 0.7, *R*_f_ (3db) = 0.6, UV detection]. IR (MIR-ATR, 4000–600 cm^−1^): *ν*_max_ = 2924, 2364, 1691, 1611, 1506, 1295, 1223, 1043, 785, 704 cm^−1^. ^1^H NMR (CDCl_3_, 400 MHz): *δ* = 7.28–7.19 (m, 1H, Ar-H), 7.18–7.09 (m, 2H, Ar-H), 7.09–6.98 (m, 4H, Ar-H), 6.95 (m, 1H, Ar-H), 6.81 (dd, *J* = 8.3 and 2.5 Hz, 1H, Ar-H), 6.76 (d, *J* = 2.5 Hz, 1H, Ar-H), 6.67 (dd, *J* = 8.3 and 2.5 Hz, 1H, Ar-H), 3.81 (s, 3H, OCH_3_), 2.32 (s, 3H, CH_3_), 2.21 (s, 3H, CH_3_), 1.77 (s, 6H, 2 × CH_3_) ppm. ^13^C{^1^H} NMR (CDCl_3_, 100 MHz): *δ* = 158.7 (s, Ar-C), 145.8 (s, Ar-C), 138.4 (s, Ar-C), 138.0 (s, Ar-C), 137.2 (s, Ar-C), 136.8 (s, Ar-C), 136.0 (s, Ar-C), 132.0 (d, Ar-CH), 129.1 (d, Ar-CH), 128.6 (d, Ar-CH), 128.3 (d, Ar-CH), 128.2 (d, Ar-CH), 127.5 (d, Ar-CH), 127.2 (d, Ar-CH), 125.8 (d, Ar-CH), 125.5 (d, Ar-CH), 125.1 (d, Ar-CH), 115.6 (s, Ar-C), 111.3 (d, Ar-CH), 108.9 (d, Ar-CH), 77.2 (s, Ar-C), 55.3 (q, OCH_3_), 26.9 (q, 2 × CH_3_), 21.4 (q, 2 × CH_3_) ppm. HR-MS (APCI+) *m*/*z* calculated for [C_26_H_27_O_2_]^+^ = [M + H]^+^: 371.2006; found 371.1992.

### 7-Methoxy-1,1-dimethyl-3,4-bis(4-methylphenyl)-1*H*-isochromene (3dc)

GP was carried out with 2-(2-bromo-5-methoxyphenyl)propan-2-ol 1d (61 mg, 0.25 mmol), 1-methyl-4-[(4-methylphenyl)ethynyl]benzene 2c (128.8 mg, 0.625 mmol), palladium acetate (2.8 mg, 5 mol%), l-proline (5.67 mg, 20 mol%), TBAI (92.3 mg, 0.25 mmol), K_2_CO_3_ (138.6 mg, 1 mmol) and water (0.5 mL). The crude material was then refined by silica gel column chromatography (petroleum ether/ethyl acetate, 99 : 1 to 98 : 2) to obtain isochromene 3dc (25.9 mg, 28%) as a brown jelly compound, [TLC control (petroleum ether/ethyl acetate 98 : 2), *R*_f_ (2c) = 0.7, *R*_f_ (3dc) = 0.6, UV detection]. IR (MIR-ATR, 4000–600 cm^−1^): *ν*_max_ = 2923, 2856, 1701, 1614, 1498, 1295, 1222, 1044, 964, 816, 756 cm^−1^. ^1^H NMR (CDCl_3_, 400 MHz): *δ* = 7.20–7.04 (m, 6H, Ar-H), 6.94 (dd, *J* = 7.8 and 1.0 Hz, 2H, Ar-H), 6.81 (d, *J* = 8.8 Hz, 1H, Ar-H), 6.76 (d, *J* = 2.9 Hz, 1H, Ar-H), 6.69–6.58 (m, 1H, Ar-H), 3.80 (s, 3H, OCH_3_), 2.37 (s, 3H, CH_3_), 2.25 (s, 3H, CH_3_), 1.74 (s, 6H, 2 × CH_3_) ppm. ^13^C{^1^H} NMR (CDCl_3_, 100 MHz): *δ* = 158.6 (s, Ar-C), 145.8 (s, Ar-C), 138.3 (s, Ar-C), 137.1 (s, Ar-C), 136.3 (s, Ar-C), 134.3 (s, Ar-C), 133.3 (s, Ar-C), 131.3 (d, 2 × Ar-CH), 129.3 (d, 2 × Ar-CH), 128.4 (d, 2 × Ar-CH), 128.1 (d, 2 × Ar-CH), 125.6 (s, Ar-C), 125.0 (d, Ar-CH), 115.0 (s, Ar-C), 111.3 (d, Ar-CH), 108.9 (d, Ar-CH), 77.2 (s, Ar-C), 55.3 (q, OCH_3_) 26.9 (q, 2 × CH_3_) 21.3 (q, CH_3_), 21.2 (q, CH_3_) ppm. HR-MS (APCI+) *m*/*z* calculated for [C_26_H_27_O_2_]^+^ = [M + H]^+^: 371.2006; found 371.1991.

### 7-Methoxy-1,1-dimethyl-3,4-dithien-2-yl-1*H*-isochromene (3dd)

GP was carried out with 2-(2-bromo-5-methoxyphenyl)propan-2-ol 1d (61 mg, 0.25 mmol), 2-(thien-2-ylethynyl)thiophene 2d (118.6 mg, 0.625 mmol), palladium acetate (2.8 mg, 5 mol%), l-proline (5.6 mg, 20 mol%), TBAI (92.3 mg, 0.25 mmol), K_2_CO_3_ (138.6 mg, 1 mmol) and water (0.5 mL). The crude material was then refined by silica gel column chromatography (petroleum ether/ethyl acetate, 99 : 1 to 98 : 2) to obtain isochromene 3dd (36.2 mg, 41%) as a brown jelly compound, [TLC control (petroleum ether/ethyl acetate 98 : 2), *R*_f_ (2d) = 0.6, *R*_f_ (3dd) = 0.5, UV detection]. IR (MIR-ATR, 4000–600 cm^−1^): *ν*_max_ = 3041, 2923, 1712, 1595, 1482, 1446, 1264, 1209, 1167, 1081, 989, 907, 745, 695 cm^−1^. ^1^H NMR (CDCl_3_, 400 MHz): *δ* = 7.51 (dd, *J* = 5.4 and 1.5 Hz, 1H, Ar-H), 7.23–7.15 (m, 2H, Ar-H), 7.04 (dd, *J* = 3.4 and 1.0 Hz, 1H, Ar-H), 6.96–6.85 (m, 3H, Ar-H), 6.75 (d, *J* = 2.9 Hz, 1H, Ar-H), 6.70 (dd, *J* = 8.8 and 3.0 Hz, 1H, Ar-H), 3.80 (s, 3H, OCH_3_), 1.73 (s, 6H, 2 × CH_3_) ppm. ^13^C{^1^H} NMR (CDCl_3_, 100 MHz): *δ* = 158.9 (s, Ar-C), 143.5 (s, Ar-C), 138.7 (s, Ar-C), 138.0 (s, Ar-C), 137.3 (s, Ar-C), 129.6 (d, Ar-CH), 127.7 (d, Ar-CH), 127.4 (d, Ar-CH), 127.1 (d, Ar-CH), 126.9 (d, Ar-CH), 126.7 (d, Ar-CH), 125.4 (s, Ar-C), 125.1 (d, Ar-CH), 111.6 (d, Ar-CH), 108.9 (d, Ar-CH), 106.3 (s, Ar-C), 78.2 (s, Ar-C), 55.4 (q, OCH_3_), 26.8 (q, 2 × CH_3_) ppm. HR-MS (APCI+) *m*/*z* calculated for [C_20_H_19_O_2_S_2_]^+^ = [M + H]^+^: 355.0821; found 355.0818.

### 7-Methoxy-3,4-bis(3-methoxyphenyl)-1,1-dimethyl-1*H*-isochromene (3de)

GP was carried out with 2-(2-bromo-5-methoxyphenyl)propan-2-ol 1d (61 mg, 0.25 mmol), 1-methoxy-3-[(3-methoxyphenyl)ethynyl]benzene 2e (148.7 mg, 0.625 mmol), palladium acetate (2.8 mg, 5 mol%), l-proline (5.6 mg, 20 mol%), TBAI (92.3 mg, 0.25 mmol), K_2_CO_3_ (138.6 mg, 1 mmol) and water (0.5 mL). The crude material was then refined by silica gel column chromatography (petroleum ether/ethyl acetate, 99 : 1 to 98 : 2) to obtain isochromene 3de (40.2 mg, 40%) as a brown jelly compound, [TLC control (petroleum ether/ethyl acetate 98 : 2), *R*_f_ (2e) = 0.6, *R*_f_ (3de) = 0.5, UV detection]. IR (MIR-ATR, 4000–600 cm^−1^): *ν*_max_ = 3087, 2974, 2838, 1608, 1487, 1425, 1297, 1216, 1043, 834, 701 cm^−1^. ^1^H NMR (CDCl_3_, 400 MHz): *δ* = 7.27 (ddd, *J* = 7.8, 7.8 and 1.0 Hz, 1H, Ar-H), 7.06 (ddd, *J* = 7.8, 7.8 and 1.0 Hz, 1H, Ar-H), 6.92 (dt, *J* = 7.8 and 1.0 Hz, 1H, Ar-H), 6.88–6.82 (m, 3H, Ar-H), 6.82–6.72 (m, 3H, Ar-H), 6.67 (m, 2H, Ar-H), 3.80 (s, 3H, OCH_3_), 3.73 (s, 3H, OCH_3_), 3.55 (s, 3H, OCH_3_), 1.75 (s, 6H, 2 × CH_3_) ppm. ^13^C{^1^H} NMR (CDCl_3_, 100 MHz): *δ* = 159.8 (s, Ar-C), 158.9 (s, Ar-C), 158.6 (s, Ar-C), 145.5 (s, Ar-C), 138.8 (s, Ar-C), 138.3 (s, Ar-C), 137.3 (s, Ar-C), 129.6 (d, Ar-CH), 128.4 (d, Ar-CH), 125.1 (d, Ar-CH), 125.1 (s, Ar-C), 124.0 (d, Ar-CH), 120.9 (d, Ar-CH), 116.8 (d, Ar-CH), 115.6 (s, Ar-C), 113.9 (q, Ar-CH), 113.5 (d, Ar-CH), 112.7 (d, Ar-CH), 111.4 (d, Ar-CH), 109.0 (d, Ar-CH), 77.4 (s, Ar-C), 55.3 (q, OCH_3_), 55.2 (q, OCH_3_), 54.9 (q, OCH_3_), 26.9 (q, 2 × CH_3_) ppm. HR-MS (APCI+) *m*/*z* calculated for [C_26_H_27_O_4_]^+^ = [M + H]^+^: 403.1904; found 403.1901.

### 6,7-Dimethoxy-1,1-dimethyl-3,4-diphenyl-1*H*-isochromene (3ea)

GP was carried out with 2-(2-bromo-4,5-dimethoxyphenyl)propan-2-ol 1e (68.5 mg, 0.25 mmol), (phenylethynyl)benzene 2a (111.2 mg, 0.625 mmol), palladium acetate (2.8 mg, 5 mol%), l-proline (5.6 mg, 20 mol%), TBAI (92.3 mg, 0.25 mmol), K_2_CO_3_ (138.6 mg, 1 mmol) and water (0.5 mL). The crude material was then refined by silica gel column chromatography (petroleum ether/ethyl acetate, 85 : 15 to 80 : 20) to obtain isochromene 3ea (37.2 mg, 40%) as a brown jelly compound, [TLC control (petroleum ether/ethyl acetate 80 : 20), *R*_f_ (2a) = 0.6, *R*_f_ (3ea) = 0.5, UV detection]. IR (MIR-ATR, 4000–600 cm^−1^): *ν*_max_ = 2942, 2843, 1603, 1505, 1452, 1242, 1171, 1026, 825, 733 cm^−1^. ^1^H NMR (CDCl_3_, 400 MHz): *δ* = 7.42–7.17 (m, 7H, Ar-H), 7.17–7.00 (m, 3H, Ar-H), 6.73 (s, 1H, Ar-H), 6.45 (s, 1H, Ar-H), 3.91 (s, 3H, OCH_3_), 3.64 (s, 3H, OCH_3_), 1.76 (s, 6H, 2 × CH_3_) ppm. ^13^C{^1^H} NMR (CDCl_3_, 100 MHz): *δ* = 148.0 (s, Ar-C), 147.9 (s, Ar-C), 146.6 (s, Ar-C), 137.2 (s, Ar-C), 136.0 (s, Ar-C), 131.5 (d, 3 × Ar-CH), 129.0 (s, Ar-C), 128.6 (d, Ar-CH), 128.6 (d, 2 × Ar-CH), 127.5 (d, 2 × Ar-CH), 127.4 (d, Ar-CH), 126.9 (d, Ar-CH), 125.3 (s, Ar-C), 115.6 (s, Ar-C), 107.6 (d, Ar-CH), 106.2 (d, Ar-CH), 77.3 (s, Ar-C), 56.2 (q, OCH_3_), 55.8 (q, OCH_3_), 27.1 (q, 2 × CH_3_) ppm. HR-MS (APCI+) *m*/*z* calculated for [C_25_H_25_O_3_]^+^ = [M + H]^+^: 373.1798; found 373.1780.

### 6,7-Dimethoxy-1,1-dimethyl-3,4-bis(3-methylphenyl)-1*H*-isochromene (3eb)

GP was carried out with 2-(2-bromo-4,5-dimethoxyphenyl)propan-2-ol 1e (68.5 mg, 0.25 mmol), 1-methyl-3-[(3-methylphenyl)ethynyl]benzene 2b (128.8 mg, 0.625 mmol), palladium acetate (2.8 mg, 5 mol%), l-proline (5.6 mg, 20 mol%), TBAI (92.3 mg, 0.25 mmol), K_2_CO_3_ (138.6 mg, 1 mmol) and water (0.5 mL). The crude material was then refined by silica gel column chromatography (petroleum ether/ethyl acetate, 93 : 7 to 90 : 10) to obtain isochromene 3eb (52 mg, 52%) as a brown jelly compound, [TLC control (petroleum ether/ethyl acetate 90 : 10), *R*_f_ (2b) = 0.6, *R*_f_ (3eb) = 0.5, UV detection]. IR (MIR-ATR, 4000–600 cm^−1^): *ν*_max_ = 3055, 2933, 1595, 1485, 1448, 1244, 1072, 1027, 747, 697 cm^−1^. ^1^H NMR (CDCl_3_, 400 MHz): *δ* = 7.22 (ddd, *J* = 7.8, 7.8 and 1.0 Hz, 1H, Ar-H), 7.16–7.07 (m, 2H, Ar-H), 7.07–7.01 (m, 2H, Ar-H), 7.01–6.89 (m, 3H, Ar-H), 6.72 (s, 1H, Ar-H), 6.46 (s, 1H, Ar-H), 3.90 (s, 3H, OCH_3_), 3.65 (s, 3H, OCH_3_), 2.30 (s, 3H, CH_3_), 2.20 (s, 3H, CH_3_), 1.76 (s, 6H, 2 × CH_3_) ppm. ^13^C{^1^H} NMR (CDCl_3_, 100 MHz): *δ* = 147.9 (s, Ar-C), 147.9 (s, Ar-C), 146.5 (s, Ar-C), 138.0 (s, Ar-C), 137.1 (s, Ar-C), 136.8 (s, Ar-C), 136.0 (s, Ar-C), 132.0 (d, Ar-CH), 129.1 (d, Ar-CH), 128.6 (d, Ar-CH), 128.3 (d, Ar-CH), 128.2 (d, Ar-CH), 127.6 (d, Ar-CH), 127.2 (d, Ar-CH), 125.8 (d, Ar-CH), 125.6 (s, Ar-C), 115.6 (s, Ar-C), 107.8 (d, Ar-CH), 106.2 (d, Ar-CH), 77.2 (s, Ar-C), 56.2 (q, OCH_3_), 55.8 (q, OCH_3_), 27.0 (q, 2 × CH_3_), 21.4 (q, 2 × CH_3_) ppm. HR-MS (APCI+) *m*/*z* calculated for [C_27_H_29_O_3_]^+^ = [M + H]^+^: 401.2111; found 401.2101.

### 6,7-Dimethoxy-1,1-dimethyl-3,4-bis(4-methylphenyl)-1*H*-isochromene (3ec)

GP was carried out with 2-(2-bromo-4,5-dimethoxyphenyl)propan-2-ol 1e (68.5 mg, 0.25 mmol), 1-methyl-4-[(4-methylphenyl)ethynyl]benzene 2c (128.8 mg, 0.625 mmol), palladium acetate (2.8 mg, 5 mol%), l-proline (5.6 mg, 20 mol%), TBAI (92.3 mg, 0.25 mmol), K_2_CO_3_ (138.6 mg, 1 mmol) and water (0.5 mL). The crude material was then refined by silica gel column chromatography (petroleum ether/ethyl acetate, 93 : 7 to 90 : 10) to obtain isochromene 3ec (44 mg, 44%) as a light yellow solid compound, melting point = 88 °C, [TLC control (petroleum ether/ethyl acetate 90 : 10), *R*_f_ (2c) = 0.6, *R*_f_ (3ec) = 0.5, UV detection]. IR (MIR-ATR, 4000–600 cm^−1^): *ν*_max_ = 3094, 2975, 2936, 2841, 1703, 1609, 1490, 1419, 1290, 1219, 1044, 835, 703 cm^−1^. ^1^H NMR (CDCl_3_, 400 MHz): *δ* = 7.18–7.11 (m, 6H, Ar-H), 6.93 (dd, *J* = 7.8 and 1.0 Hz, 2H, Ar-H), 6.72 (s, 1H, Ar-H), 6.47 (s, 1H, Ar-H), 3.91 (s, 3H, OCH_3_), 3.66 (s, 3H, OCH_3_), 2.37 (s, 3H, CH_3_), 2.25 (s, 3H, CH_3_), 1.75 (s, 6H, 2 × CH_3_) ppm. ^13^C{^1^H} NMR (CDCl_3_, 100 MHz): *δ* = 147.9 (s, Ar-C), 147.8 (s, Ar-C), 146.5 (s, Ar-C), 137.2 (s, Ar-C), 136.3 (s, Ar-C), 134.2 (s, Ar-C), 133.3 (s, Ar-C), 131.3 (d, 2 × Ar-CH), 129.3 (d, 2 × Ar-CH), 129.1 (s, Ar-C), 128.5 (d, 2 × Ar-CH), 128.1 (d, 2 × Ar-CH), 125.7 (s, Ar-C), 114.9 (s, Ar-C), 107.6 (d, Ar-CH), 106.2 (d, Ar-CH), 77.1 (s, Ar-C), 56.2 (q, OCH_3_), 55.8 (q, OCH_3_), 27.0 (q, 2 × CH_3_), 21.3 (q, CH_3_), 21.2 (q, CH_3_) ppm. HR-MS (APCI+) *m*/*z* calculated for [C_27_H_29_O_3_]^+^ = [M + H]^+^: 401.2111; found 401.2107.

### 6,7-Dimethoxy-3,4-bis(3-methoxyphenyl)-1,1-dimethyl-1*H*-isochromene (3ee)

GP was carried out with 2-(2-bromo-4,5-dimethoxyphenyl)propan-2-ol 1e (68.5 mg, 0.25 mmol), 1-methoxy-3-[(3-methoxyphenyl)ethynyl]benzene 2e (148.7 mg, 0.625 mmol), palladium acetate (2.8 mg, 5 mol%), l-proline (5.6 mg, 20 mol%), TBAI (92.3 mg, 0.25 mmol), K_2_CO_3_ (138.6 mg, 1 mmol) and water (0.5 mL). The crude material was then refined by silica gel column chromatography (petroleum ether/ethyl acetate, 73 : 27 to 70 : 30) to obtain isochromene 3ee (44.2 mg, 41%) as a brown jelly compound, [TLC control (petroleum ether/ethyl acetate 70 : 30), *R*_f_ (2e) = 0.5, *R*_f_ (3ee) = 0.4, UV detection]. IR (MIR-ATR, 4000–600 cm^−1^): *ν*_max_ = 2944, 2834, 1597, 1504, 1452, 1237, 1163, 1105, 1020, 825, 741 cm^−1^. ^1^H NMR (CDCl_3_, 400 MHz): *δ* = 7.28 (ddd, *J* = 7.8, 7.8 and 2.4 Hz, 1H, Ar-H), 7.06 (ddd, *J* = 7.8, 7.8 and 2.4 Hz, 1H, Ar-H), 6.94–6.88 (m, 2H, Ar-H), 6.87–6.82 (m, 1H, Ar-H), 6.82–6.76 (m, 2H, Ar-H), 6.72 (s, 1H, Ar-H), 6.71–6.66 (m, 1H, Ar-H), 6.49 (s, 1H, Ar-H), 3.91 (s, 3H, OCH_3_), 3.73 (s, 3H, OCH_3_), 3.66 (s, 3H, OCH_3_), 3.56 (s, 3H, OCH_3_), 1.75 (s, 6H, 2 × CH_3_) ppm. ^13^C{^1^H} NMR (CDCl_3_, 100 MHz): *δ* = 159.9 (s, Ar-C), 158.6 (s, Ar-C), 148.1 (s, Ar-C), 147.9 (s, Ar-C), 146.2 (s, Ar-C), 138.7 (s, Ar-C), 137.3 (s, Ar-C), 129.6 (d, Ar-CH), 129.1 (s, Ar-C), 128.4 (d, Ar-CH), 125.1 (s, Ar-C), 123.9 (d, Ar-CH), 121.0 (d, Ar-CH), 116.7 (d, Ar-CH), 115.5 (s, Ar-C), 114.0 (d, Ar-CH), 113.5 (d, Ar-CH), 112.8 (d, Ar-CH), 107.7 (d, Ar-CH), 106.3 (d, Ar-CH), 77.3 (s, Ar-C), 56.2 (q, OCH_3_), 55.8 (q, OCH_3_), 55.2 (q, OCH_3_), 54.9 (q, OCH_3_), 27.1 (q, 2 × CH_3_) ppm. HR-MS (APCI+) *m*/*z* calculated for [C_27_H_29_O_5_]^+^ = [M + H]^+^: 433.2010; found 433.2016.

### 6,7-Dimethoxy-3,4-bis(4-methoxyphenyl)-1,1-dimethyl-1*H*-isochromene (3ef)

GP was carried out with 2-(2-bromo-4,5-dimethoxyphenyl)propan-2-ol 1e (68.5 mg, 0.25 mmol), 1-methoxy-4-[(4-methoxyphenyl)ethynyl]benzene 2f (148.7 mg, 0.625 mmol), palladium acetate (2.8 mg, 5 mol%), l-proline (5.6 mg, 20 mol%), TBAI (92.3 mg, 0.25 mmol), K_2_CO_3_ (138.6 mg, 1 mmol) and water (0.5 mL). The crude material was then refined by silica gel column chromatography (petroleum ether/ethyl acetate, 73 : 27 to 70 : 30) to obtain isochromene 3ef (52.9 mg, 49%) as a light yellow jelly compound, [TLC control (petroleum ether/ethyl acetate 70 : 30), *R*_f_ (3f) = 0.5, *R*_f_ (3ef) = 0.4, UV detection]. IR (MIR-ATR, 4000–600 cm^−1^): *ν*_max_ = 3090, 1792, 1652, 1529, 1409, 1266, 1198, 834, 697 cm^−1^. ^1^H NMR (CDCl_3_, 400 MHz): *δ* = 7.23–7.12 (m, 4H, Ar-H), 6.94–6.84 (m, 2H, Ar-H), 6.71 (s, 1H, Ar-H), 6.70–6.61 (m, 2H, Ar-H), 6.47 (s, 1H, Ar-H), 3.90 (s, 3H, OCH_3_), 3.83 (s, 3H, OCH_3_), 3.73 (s, 3H, OCH_3_), 3.66 (s, 3H, OCH_3_), 1.74 (s, 6H, 2 × CH_3_) ppm. ^13^C{^1^H} NMR (CDCl_3_, 100 MHz): *δ* = 158.7 (s, Ar-C), 158.4 (s, Ar-C), 147.9 (s, Ar-C), 147.7 (s, Ar-C), 146.4 (s, Ar-C), 132.5 (d, 2 × Ar-CH), 129.9 (d, 2 × Ar-CH), 129.6 (s, Ar-C), 128.9 (s, Ar-C), 128.7 (s, Ar-C), 125.9 (s, Ar-C), 114.0 (d, 2 × Ar-CH), 113.9 (s, Ar-C), 112.8 (d, 2 × Ar-CH), 107.5 (d, Ar-CH), 106.3 (d, Ar-CH), 77.1 (s, Ar-C), 56.2 (q, OCH_3_), 55.8 (q, OCH_3_), 55.1 (q, OCH_3_), 55.0 (q, OCH_3_), 27.0 (q, 2 × CH_3_) ppm. HR-MS (APCI+) *m*/*z* calculated for [C_27_H_29_O_5_]^+^ = [M + H]^+^: 433.2010; found 433.2018.

### 5,6,7-Trimethoxy-1,1-dimethyl-3,4-diphenyl-1*H*-isochromene (3fa)

GP was carried out with 2-(2-bromo-3,4,5-trimethoxyphenyl)propan-2-ol 1f (76 mg, 0.25 mmol), (phenylethynyl)benzene 2a (111.2 mg, 0.625 mmol), palladium acetate (2.8 mg, 5 mol%), l-proline (5.6 mg, 20 mol%), TBAI (92.3 mg, 0.25 mmol), K_2_CO_3_ (138.6 mg, 1 mmol) and water (0.5 mL). The crude material was then refined by silica gel column chromatography (petroleum ether/ethyl acetate, 85 : 15 to 80 : 20) to obtain isochromene 3fa (19 mg, 19%) as a brown jelly compound, [TLC control (petroleum ether/ethyl acetate 80 : 20), *R*_f_ (2a) = 0.6, *R*_f_ (3fa) = 0.5, UV detection]. IR (MIR-ATR, 4000–600 cm^−1^): *ν*_max_ = 2936, 2364, 1742, 1695, 1649, 1515, 1461, 1258, 1099, 1025, 758, 700 cm^−1^. ^1^H NMR (CDCl_3_, 400 MHz): *δ* = 7.24–7.19 (m, 4H, Ar-H), 7.18–7.12 (m, 3H, Ar-H), 7.11–7.08 (m, 3H, Ar-H), 6.57 (s, 1H, Ar-H), 3.91 (s, 3H, OCH_3_), 3.78 (s, 3H, OCH_3_), 3.09 (s, 3H, OCH_3_), 1.75 (s, 6H, 2 × CH_3_) ppm. ^13^C{^1^H} NMR (CDCl_3_, 100 MHz): *δ* = 152.6 (s, Ar-C), 150.4 (s, Ar-C), 148.1 (s, Ar-C), 142.3 (s, Ar-C), 139.6 (s, Ar-C), 136.6 (s, Ar-C), 134.6 (s, Ar-C), 130.9 (d, 2 × Ar-CH), 128.6 (d, 2 × Ar-CH), 127.4 (d, 2 × Ar-CH), 127.3 (d, Ar-CH), 127.3 (d, 2 × Ar-CH), 125.9 (d, Ar-CH), 119.2 (s, Ar-C), 115.3 (s, Ar-C), 102.1 (d, Ar-CH), 77.6 (s, Ar-C), 60.6 (q, OCH_3_), 60.1 (q, OCH_3_), 56.2 (q, OCH_3_), 26.6 (q, 2 × CH_3_) ppm. HR-MS (APCI+) *m*/*z* calculated for [C_26_H_27_O_4_]^+^ = [M + H]^+^: 403.1904; found 403.1907.

### 5,6,7-Trimethoxy-1,1-dimethyl-3,4-bis(3-methylphenyl)-1*H*-isochromene (3fb)

GP was carried out with 2-(2-bromo-3,4,5-trimethoxyphenyl)propan-2-ol 1f (76 mg, 0.25 mmol),1-methyl-3-[(3-methylphenyl)ethynyl]benzene 2b (128.8 mg, 0.625 mmol), palladium acetate (2.8 mg, 5 mol%), l-proline (5.6 mg, 20 mol%), TBAI (92.3 mg, 0.25 mmol), K_2_CO_3_ (138.6 mg, 1 mmol) and water (0.5 mL). The crude material was then refined by silica gel column chromatography (petroleum ether/ethyl acetate, 93 : 7 to 90 : 10) to obtain isochromene 3fb (21.5 mg, 20%) as a brown jelly compound, [TLC control (petroleum ether/ethyl acetate 90 : 10), *R*_f_ (2b) = 0.5, *R*_f_ (3fb) = 0.4, UV detection]. IR (MIR-ATR, 4000–600 cm^−1^): *ν*_max_ = 2926, 2856, 2365, 1696, 1650, 1514, 1463, 1097, 753 cm^−1^. ^1^H NMR (CDCl_3_, 400 MHz): *δ* = 7.08 (ddd, *J* = 7.8, 7.8 and 1.0 Hz, 1H, Ar-H), 7.05–7.01 (m, 3H, Ar-H), 7.00–6.86 (m, 4H, Ar-H), 6.54 (s, 1H, Ar-H), 3.89 (s, 3H, OCH_3_), 3.76 (s, 3H, OCH_3_), 3.09 (s, 3H, OCH_3_), 2.26 (s, 3H, CH_3_), 2.19 (s, 3H, CH_3_) 1.72 (s, 6H, 2 × CH_3_) ppm. ^13^C{^1^H} NMR (CDCl_3_, 100 MHz): *δ* = 152.5 (s, Ar-C), 150.4 (s, Ar-C), 147.9 (s, Ar-C), 142.3 (s, Ar-C), 139.5 (s, Ar-C), 136.7 (s, Ar-C), 136.5 (s, Ar-C), 136.4 (s, Ar-C), 134.8 (s, Ar-C), 131.3 (d, Ar-CH), 129.2 (d, Ar-CH), 128.1 (d, Ar-CH), 128.0 (d, Ar-CH), 127.1 (d, 2 × Ar-CH), 126.5 (d, Ar-CH), 125.7 (d, Ar-CH), 119.5 (s, Ar-C), 115.4 (s, Ar-C), 102.0 (d, Ar-CH), 77.5 (s, Ar-C), 60.6 (s, Ar-C), 60.1 (q, OCH_3_), 56.1 (q, OCH_3_), 26.6 (q, 2 × CH_3_), 21.4 (q, 2 × CH_3_) ppm. HR-MS (APCI+) *m*/*z* calculated for [C_28_H_31_O_4_]^+^ = [M + H]^+^: 431.2217; found 431.2214.

### 5,6,7-Trimethoxy-1,1-dimethyl-3,4-bis(4-methylphenyl)-1*H*-isochromene (3fc)

GP was carried out with 2-(2-bromo-3,4,5-trimethoxyphenyl)propan-2-ol 1f (76 mg, 0.25 mmol),1-methyl-4-[(4-methylphenyl)ethynyl]benzene 2c (128.8 mg, 0.625 mmol), palladium acetate (2.8 mg, 5 mol%), l-proline (5.6 mg, 20 mol%), TBAI (92.3 mg, 0.25 mmol), K_2_CO_3_ (138.6 mg, 1 mmol) and water (0.5 mL). The crude material was then refined by silica gel column chromatography (petroleum ether/ethyl acetate, 90 : 10 to 85 : 15) to obtain isochromene 3fc (21.5 mg, 20%) as a brown jelly compound, [TLC control (petroleum ether/ethyl acetate 85 : 15), *R*_f_ (2c) = 0.5, *R*_f_ (3fc) = 0.4, UV detection]. IR (MIR-ATR, 4000–600 cm^−1^): *ν*_max_ = 2925, 2855, 2365, 1742, 1697, 1649, 1512, 1462, 1232, 1158, 1099, 828, 758, 700 cm^−1^. ^1^H NMR (CDCl_3_, 400 MHz): *δ* = 7.15–7.06 (m, 4H, Ar-H), 7.01 (d, *J* = 7.3 Hz, 2H, Ar-H), 6.92 (d, *J* = 7.8 Hz, 2H, Ar-H), 6.53 (s, 1H, Ar-H), 3.88 (s, 3H, OCH_3_), 3.75 (s, 3H, OCH_3_), 3.07 (s, 3H, OCH_3_), 2.30 (s, 3H, CH_3_), 2.23 (s, 3H, CH_3_), 1.70 (s, 6H, 2 × CH_3_) ppm. ^13^C{^1^H} NMR (CDCl_3_, 100 MHz): *δ* = 152.4 (s, Ar-C), 150.4 (s, Ar-C), 147.9 (s, Ar-C), 142.3 (s, Ar-C), 137.0 (s, Ar-C), 136.5 (s, Ar-C), 135.2 (s, Ar-C), 134.7 (s, Ar-C), 133.8 (s, Ar-C), 130.7 (d, 2 × Ar-CH), 128.5 (d, 2 × Ar-CH), 128.1 (d, 2 × Ar-CH), 128.0 (d, 2 × Ar-CH), 119.6 (s, Ar-C), 114.9 (s, Ar-C), 102.0 (d, Ar-CH), 77.2 (s, Ar-C), 60.6 (q, OCH_3_), 60.2 (q, OCH_3_), 56.1 (q, OCH_3_), 26.6 (q, 2 × CH_3_), 21.3 (q, CH_3_), 21.2 (q, CH_3_) ppm. HR-MS (APCI+) *m*/*z* calculated for [C_28_H_31_O_4_]^+^ = [M + H]^+^: 431.2217; found 431.2220.

### 7-Chloro-1,1-dimethyl-3,4-diphenyl-1*H*-isochromene (3ga)

GP was carried out with 2-(2-bromo-5-chlorophenyl)propan-2-ol 1g (61.9 mg, 0.25 mmol), (phenylethynyl)benzene 2a (111.2 mg, 0.625 mmol), palladium acetate (2.8 mg, 5 mol%), l-proline (5.6 mg, 20 mol%), TBAI (92.3 mg, 0.25 mmol), K_2_CO_3_ (138.6 mg, 1 mmol) and water (0.5 mL). The crude material was then refined by silica gel column chromatography (petroleum ether/ethyl acetate, 100 : 1 to 98 : 2) to obtain isochromene 3ga (51.8 mg, 60%) as a light yellow jelly compound, [TLC control (petroleum ether/ethyl acetate 98 : 2), *R*_f_ (2a) = 0.7, *R*_f_ (3ga) = 0.6, UV detection]. IR (MIR-ATR, 4000–600 cm^−1^): *ν*_max_ = 3405, 3042, 1692, 1602, 1453, 1320, 1260, 1088, 1025, 793, 744, 700 cm^−1^. ^1^H NMR (CDCl_3_, 400 MHz): *δ* = 7.36–7.28 (3H, m), 7.25–7.18 (4H, m), 7.18–7.11 (4H, m), 7.07 (1H, dd, *J* = 8.4, 2.1 Hz), 6.80 (1H, d, *J* = 8.4 Hz), 1.76 (6H, s) ppm. ^13^C{^1^H} NMR (CDCl_3_, 100 MHz): *δ* = 148.3 (s, Ar-C), 137.9 (s, Ar-C), 136.6 (s, Ar-C), 135.6 (s, Ar-C), 132.1 (s, Ar-C), 131.5 (d, 2 × Ar-CH), 128.8 (d, 2 × Ar-CH), 128.7 (d, 2 × Ar-CH), 127.9 (d, Ar-CH), 127.5 (d, 2 × Ar-CH), 127.2 (d, Ar-CH), 127.1 (d, Ar-CH), 125.9 (s, Ar-C), 125.0 (d, Ar-CH), 122.5 (d, Ar-CH), 114.9 (s, Ar-C), 77.4 (s, Ar-C), 26.9 (q, 2 × CH_3_) ppm.

### 3,4-Diphenyl-1-propyl-1*H*-isochromene (3ha)

GP was carried out with 1-(2-bromophenyl)butan-1-ol 1h (57 mg, 0.25 mmol), (phenylethynyl)benzene 2a (111.2 mg, 0.62 mmol), palladium acetate (2.8 mg, 5 mol%), l-proline (5.6 mg, 20 mol%), TBAI (92.3 mg, 0.25 mmol), K_2_CO_3_ (138.6 mg, 1 mmol) and water (0.5 mL). The crude material was then refined by silica gel column chromatography (petroleum ether/ethyl acetate, 99 : 1 to 98 : 2) to obtain isochromene 3ha (33.4 mg, 41%) as a light yellow jelly compound, [TLC control (petroleum ether/ethyl acetate 98 : 2), *R*_f_ (2a) = 0.8, *R*_f_ (3ha) = 0.7, UV detection]. IR (MIR-ATR, 4000–600 cm^−1^): *ν*_max_ = 3085, 2973, 2839, 1607, 1486, 1424, 1295, 1211, 1040, 831, 698 cm^−1^. ^1^H NMR (CDCl_3_, 400 MHz): *δ* = 7.40–7.28 (m, 3H, Ar-H), 7.28–7.20 (m, 4H, Ar-H), 7.20–7.03 (m, 6H, Ar-H), 6.86 (dd, *J* = 7.3 and 1.0 Hz, 1H, Ar-H), 5.28 (dd, *J* = 8.3 and 4.9 Hz, 1H, Ar-H), 2.29–2.11 (m, 1H, CH_2_), 2.02–1.84 (m, 1H, CH_2_), 1.77–1.61 (m, 1H, CH_2_) 1.60–1.46 (m, 1H, CH_2_), 1.01 (t, *J* = 7.34 Hz, 3H, CH_3_) ppm. ^13^C{^1^H} NMR (CDCl_3_, 100 MHz): *δ* = 148.9 (s, Ar-C), 137.0 (s, Ar-C), 135.7 (s, Ar-C), 132.8 (s, Ar-C), 131.6 (d, 2 × Ar-CH), 128.7 (d, Ar-CH), 128.7 (s, Ar-C), 128.6 (d, 2 × Ar-CH), 127.7 (s, Ar-C), 127.1 (d, Ar-CH), 127.5 (d, Ar-CH), 127.4 (d, 2 × Ar-CH), 126.9 (d, Ar-CH), 126.5 (d, Ar-CH), 123.6 (d, Ar-CH), 123.2 (d, Ar-CH), 115.9 (s, Ar-C), 77.4 (s, Ar-C), 36.0 (t, CH_2_), 18.7 (t, CH_2_), 14.0 (q, CH_3_) ppm. HR-MS (APCI+) *m*/*z* calculated for [C_24_H_23_O]^+^ = [M + H]^+^: 327.1743; found 327.1746.

### 3,4-Bis(4-fluorophenyl)-1-propyl-1*H*-isochromene (3hh)

GP was carried out with 1-(2-bromophenyl)butan-1-ol 1h (57 mg, 0.25 mmol), 1-fluoro-4-[(4-fluorophenyl)ethynyl]benzene 2h (133.7 mg, 0.625 mmol), palladium acetate (2.8 mg, 5 mol%), l-proline (5.6 mg, 20 mol%), TBAI (92.3 mg, 0.25 mmol), K_2_CO_3_ (138.6 mg, 1 mmol) and water (0.5 mL). The crude material was then refined by silica gel column chromatography (petroleum ether/ethyl acetate, 100 : 0 to 99 : 1) to obtain isochromene 3hh (39.8 mg, 44%) as a light yellow jelly compound, [TLC control (petroleum ether/ethyl acetate 100 : 0), *R*_f_ (2h) = 0.7, *R*_f_ (3hh) = 0.6, UV detection]. IR (MIR-ATR, 4000–600 cm^−1^): *ν*_max_ = 3096, 2974, 2930, 2842, 1610, 1491, 1423, 1297, 1220, 1044, 837, 701 cm^−1^. ^1^H NMR (CDCl_3_, 400 MHz): *δ* = 7.24–7.13 (m, 6H, Ar-H), 7.13–6.99 (m, 3H, Ar-H), 6.91–6.77 (m, 3H, Ar-H), 5.26 (dd, *J* = 8.6 and 5.1 Hz, 1H, Ar-H), 2.24–2.07 (m, 1H, CH_2_), 1.99–1.83 (m, 1H, CH_2_), 1.73–1.46 (m, 2H, CH_2_), 1.01 (t, *J* = 7.3 Hz, 3H, CH_3_) ppm. ^13^C{^1^H} NMR (CDCl_3_, 100 MHz): *δ* = 162.1 (d, *J* = 248.0 Hz, Ar-C), 161.9 (s, *J* = 246.5 Hz, Ar-C), 148.3 (s, Ar-C), 133.2 (d, Ar-CH), 133.1 (d, Ar-CH), 132.1 (d, *J* = 103.4 Hz, Ar-C), 131.9 (d, *J* = 103.4 Hz, Ar-C), 131.5 (d, *J* = 103.4 Hz, Ar-C), 130.6 (d, Ar-CH), 130.5 (d, Ar-CH), 127.6 (d, 2 × Ar-CH), 126.7 (d, 2 × Ar-CH), 123.7 (d, 2 × Ar-CH), 123.0 (d, 2 × Ar-CH), 115.7 (d, *J* = 21.3 Hz, Ar-CH), 114.6 (d, *J* = 21.3 Hz, Ar-CH), 77.5 (d, Ar-CH) 35.9 (t, CH_2_) 18.70 (t, CH_2_) 14.03 (q, CH_3_) ppm. HR-MS (APCI+) *m*/*z* calculated for [C_24_H_21_F_2_O]^+^ = [M + H]^+^: 363.1555; found 363.1563.

## Conflicts of interest

There are no conflicts to declare.

## Supplementary Material

RA-010-C9RA08792C-s001
