# Supplementary material for: Palladium mediated domino reaction: synthesis of isochromenes under aqueous medium
Source: RSC Adv. 2020 Jan 2;10(1):338–49. doi: 10.1039/c9ra08792c (PMC9047682; doi:10.1039/c9ra08792c)
Supplement: RA-010-C9RA08792C-s001 [file RA-010-C9RA08792C-s001.pdf]

Electronic Supplementary Material (ESI) for RSC Advances.
This journal is © The Royal Society of Chemistry 2019

Supporting Information

Palladium Mediated Domino Reaction: Synthesis of Isochromenes Under Aqueous Medium

Lavisha Punia, Karu Ramesh and Gedu Satyanarayana*

Department of Chemistry, Indian Institute of Technology Hyderabad, Kandi – 502 285, Sangareddy, Telangana, INDIA.

Phone: (040) 2301 6033; Fax: (040) 2301 6003 / 32, E-mail: gvsatya@iith.ac.in

^{1}H & ^{13}C-NMR Spectra all new compounds S3-S30

Table S1: Substituted *ortho*-bromobenzyl alcohols **1a**-**1h**.

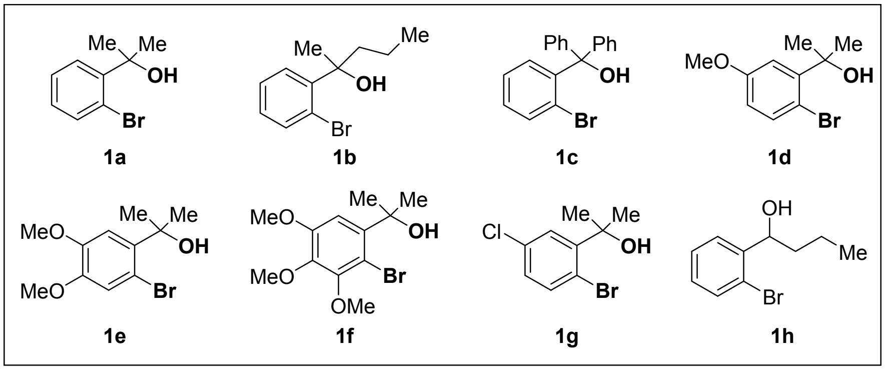

Table S2: Biaryl acetylenes **2a**-**2k**.

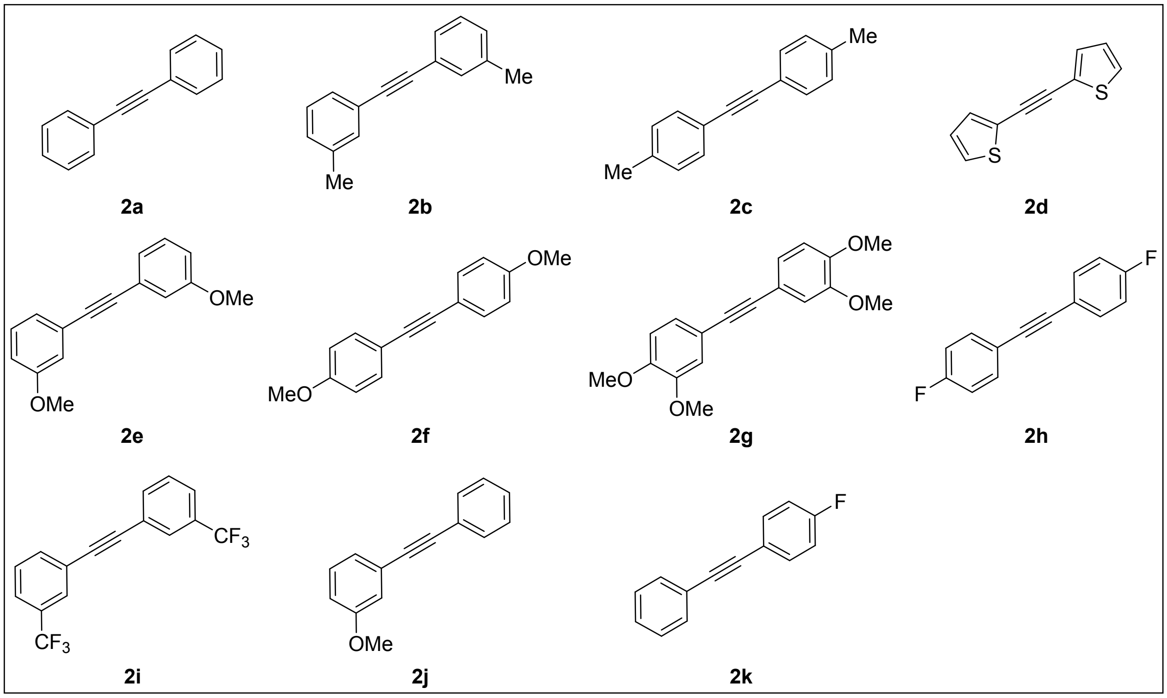

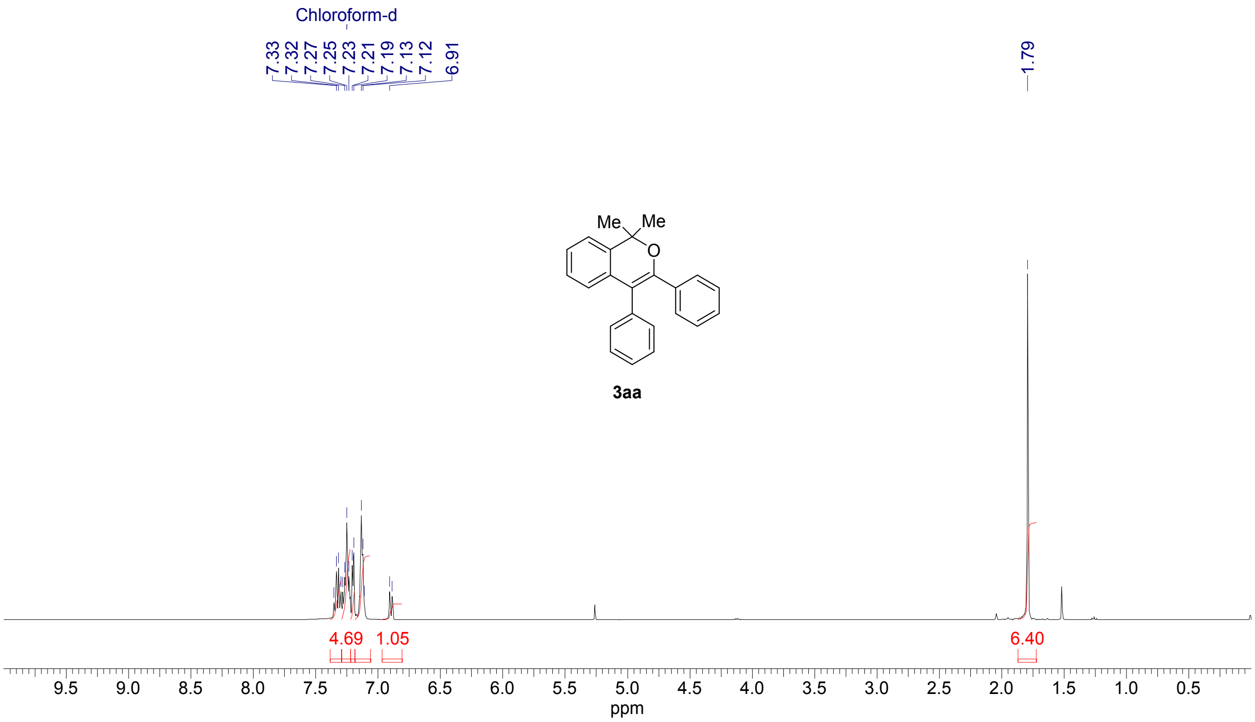

^{1}H-NMR (400 MHz) spectrum of **3aa** in $CDCl_3$

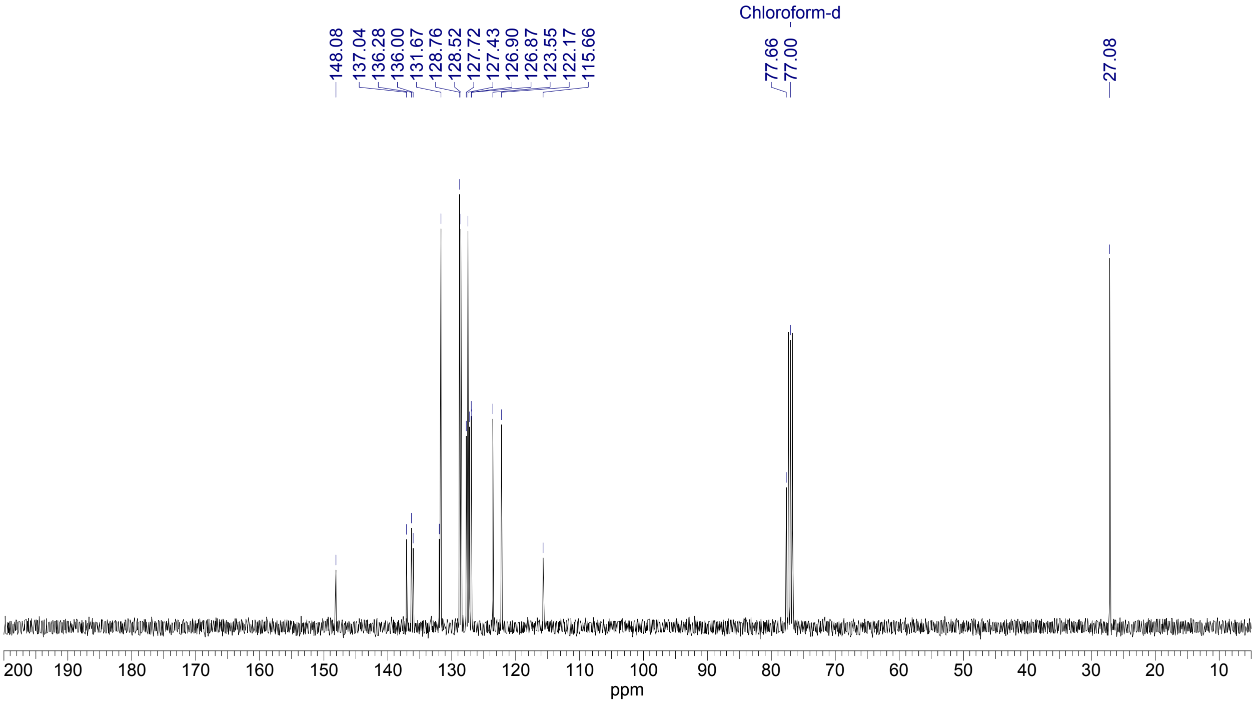

^{13}C{^{1}H}-NMR (100 MHz) spectrum of **3aa** in $CDCl_3$

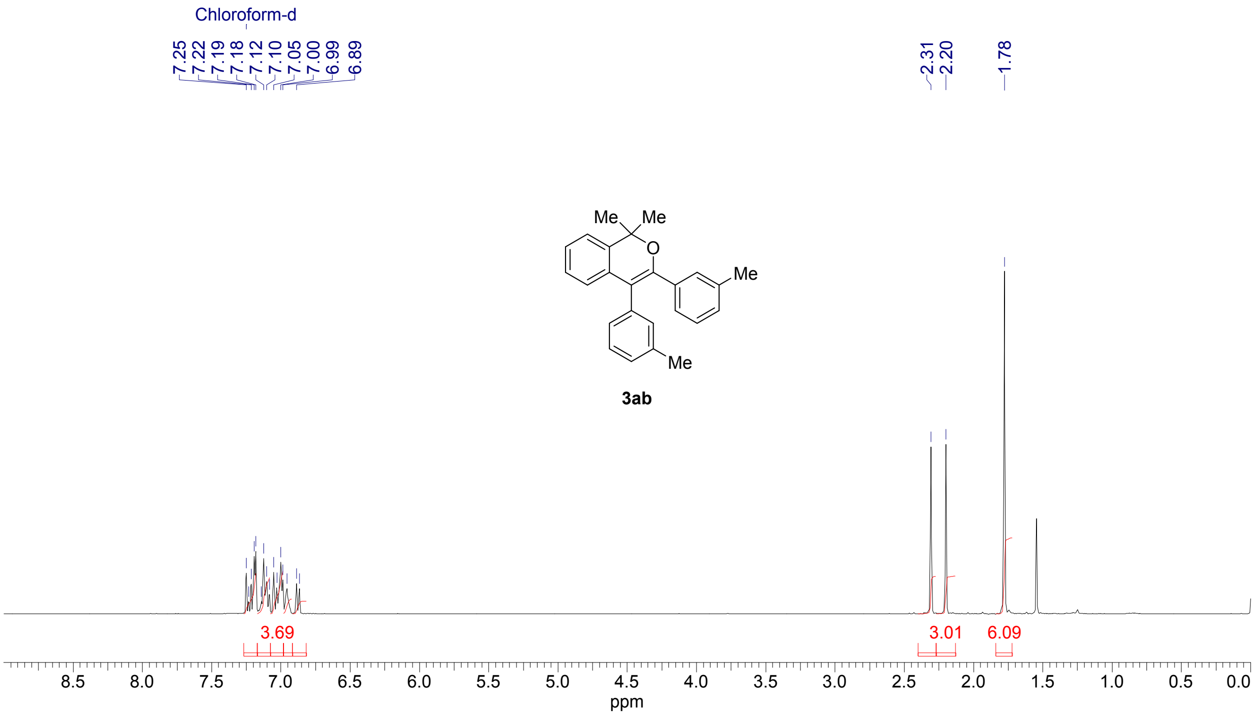

^{1}H-NMR (400 MHz) spectrum of **3ab** in $CDCl_3$

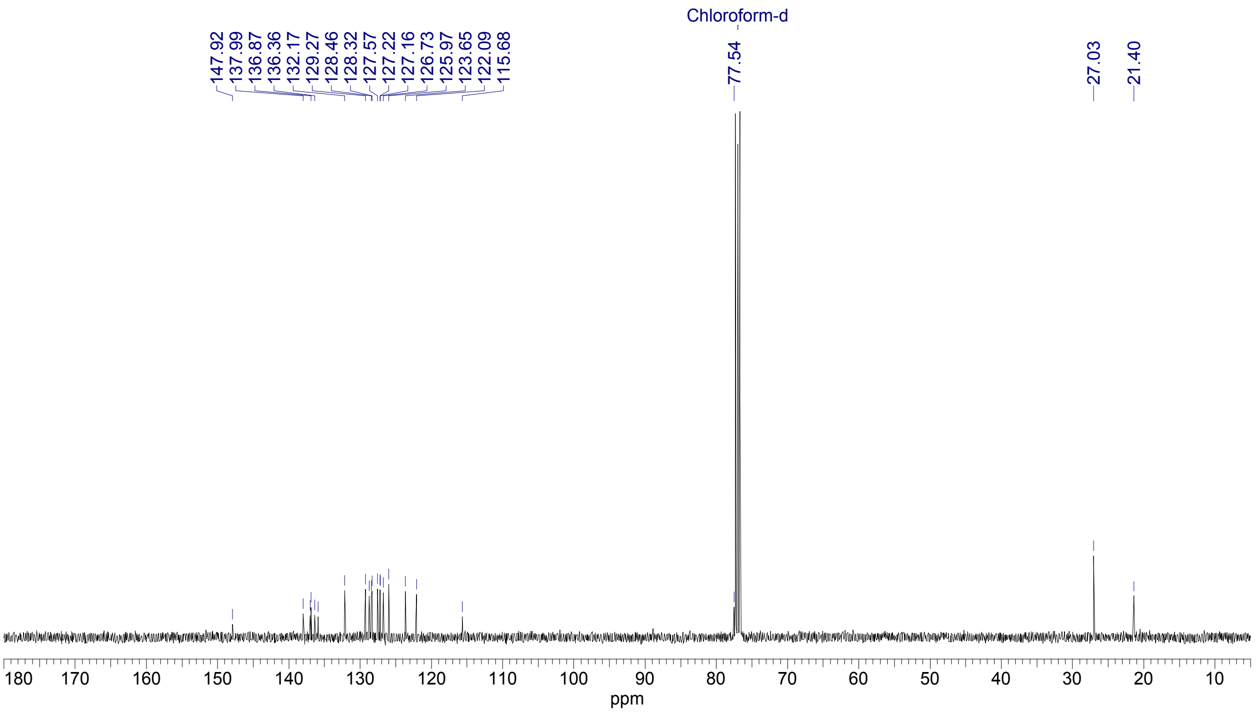

^{13}C{^{1}H}-NMR (100 MHz) spectrum of **3ab** in $CDCl_3$

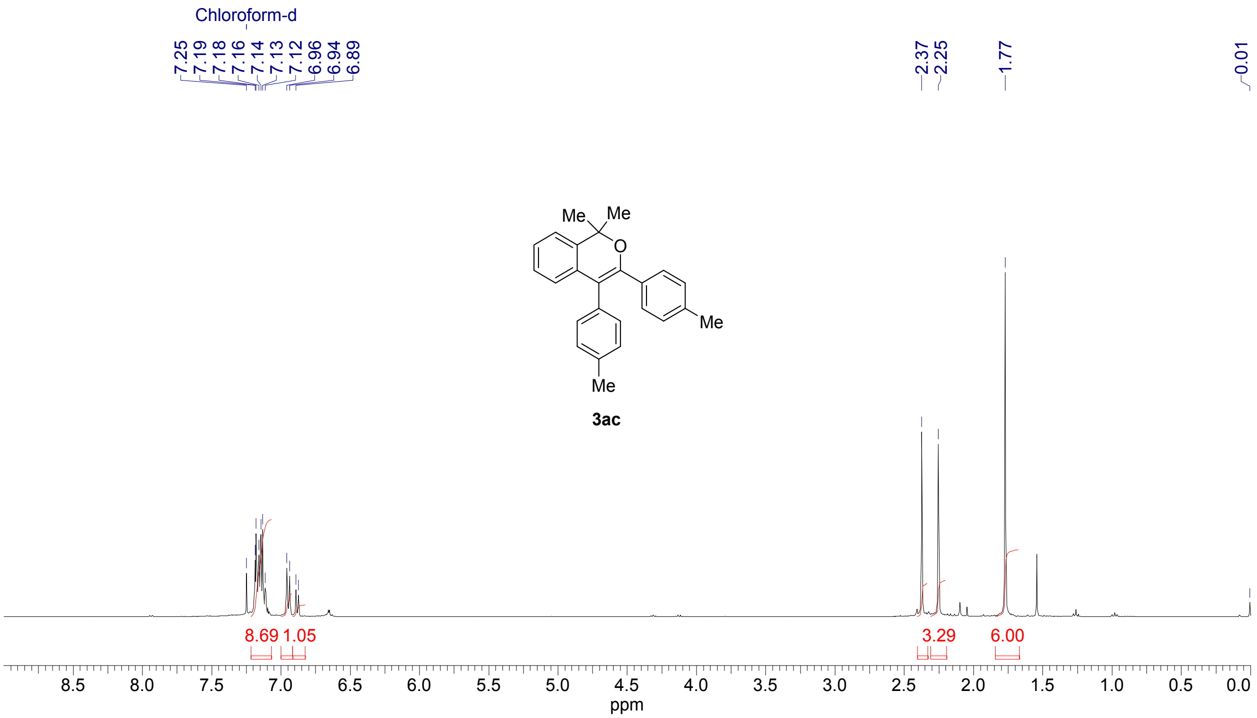

^{1}H-NMR (400 MHz) spectrum of **3ac** in $CDCl_3$

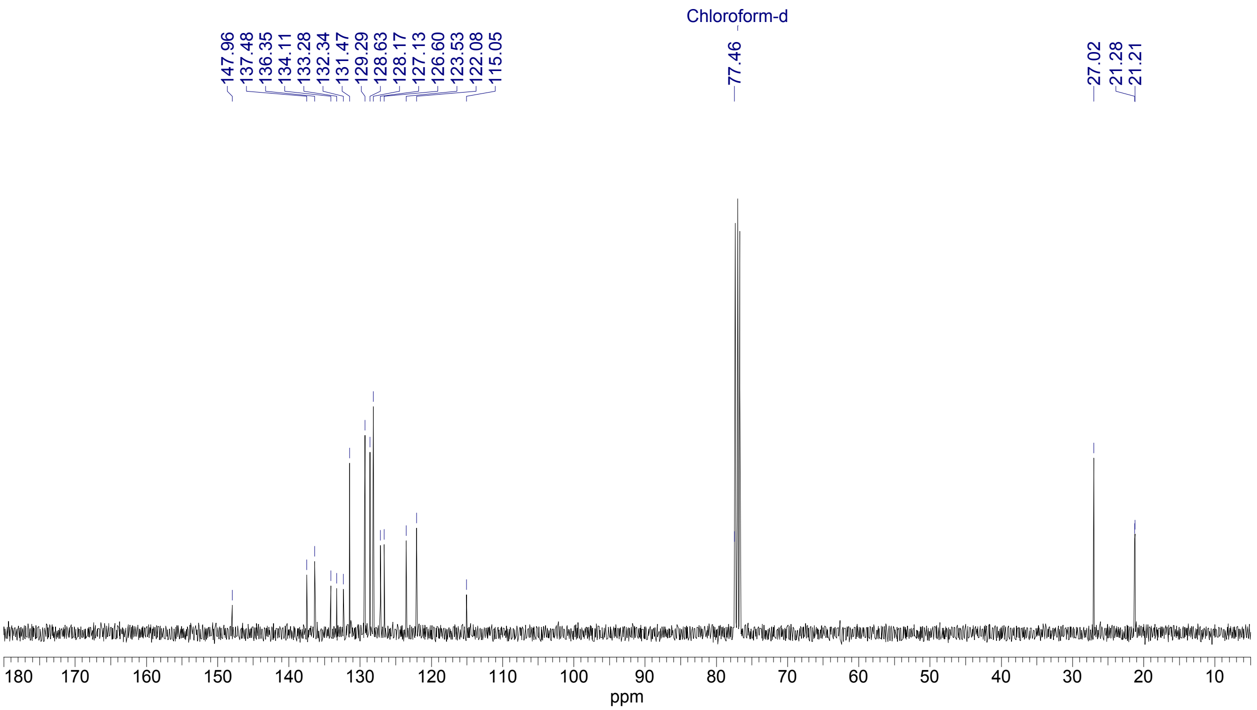

^{13}C{^{1}H}-NMR (100 MHz) spectrum of **3ac** in $CDCl_3$

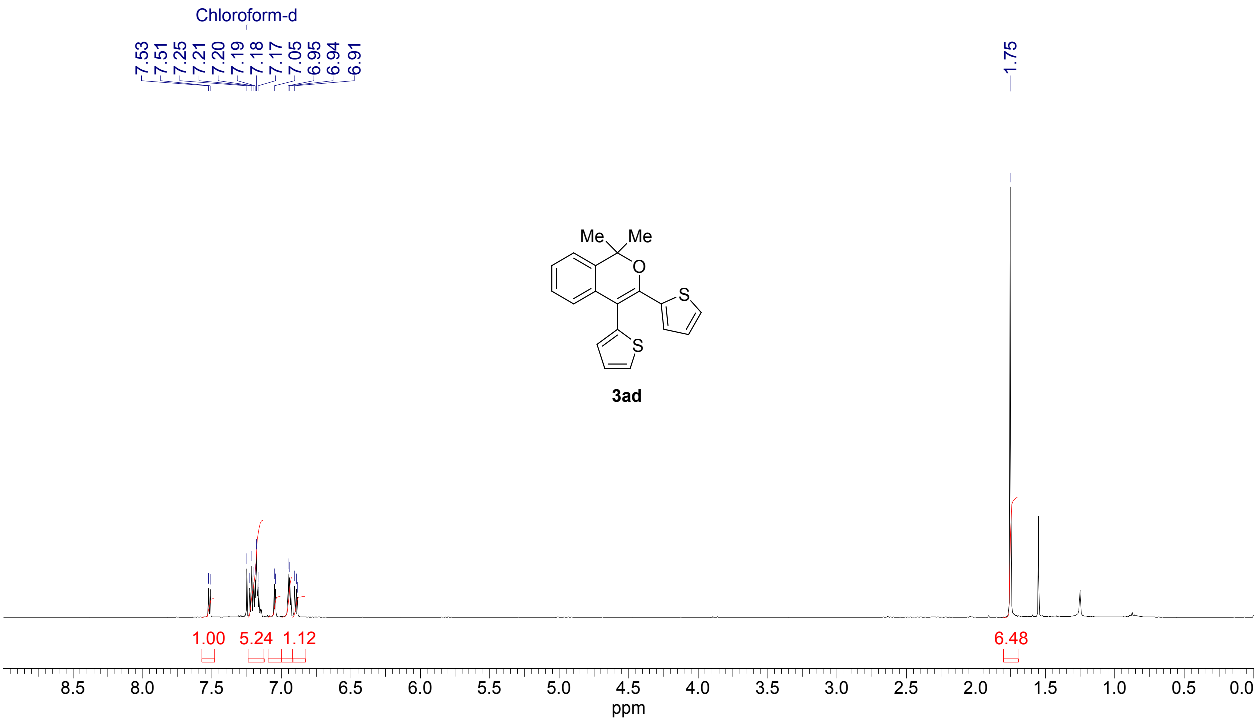

^{1}H-NMR (400 MHz) spectrum of **3ad** in $CDCl_3$

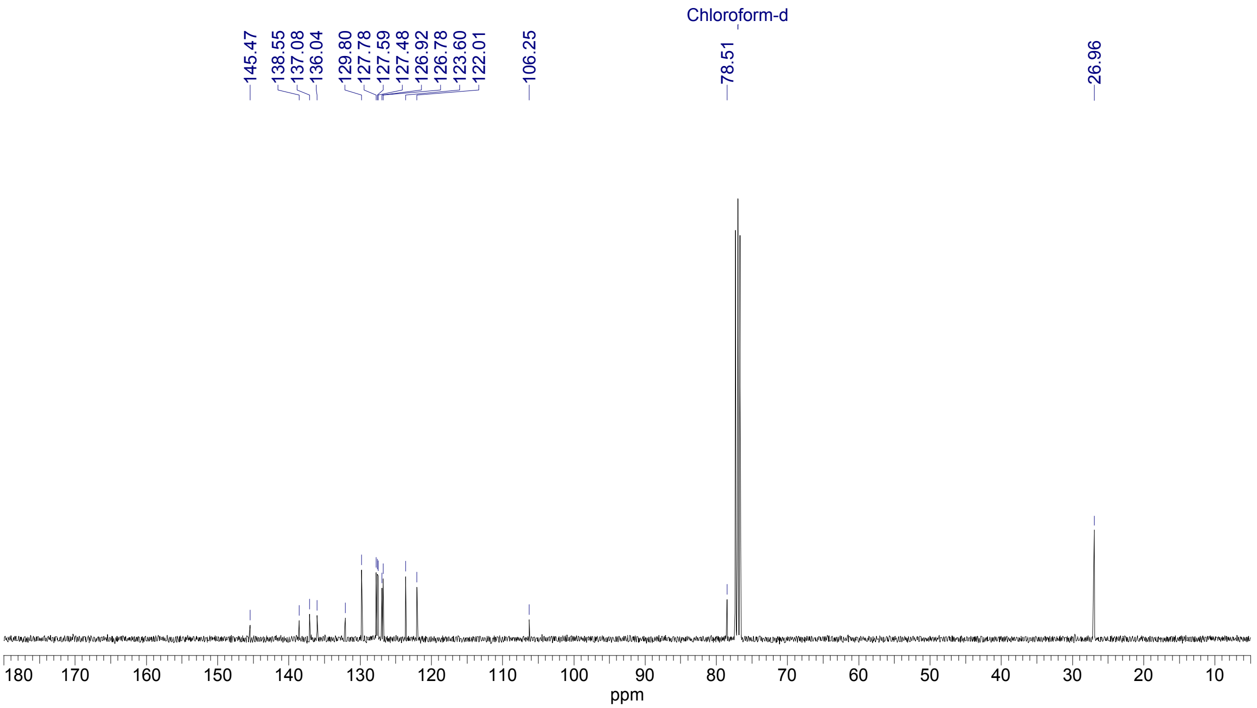

^{13}C{^{1}H}-NMR (100 MHz) spectrum of **3ad** in $CDCl_3$

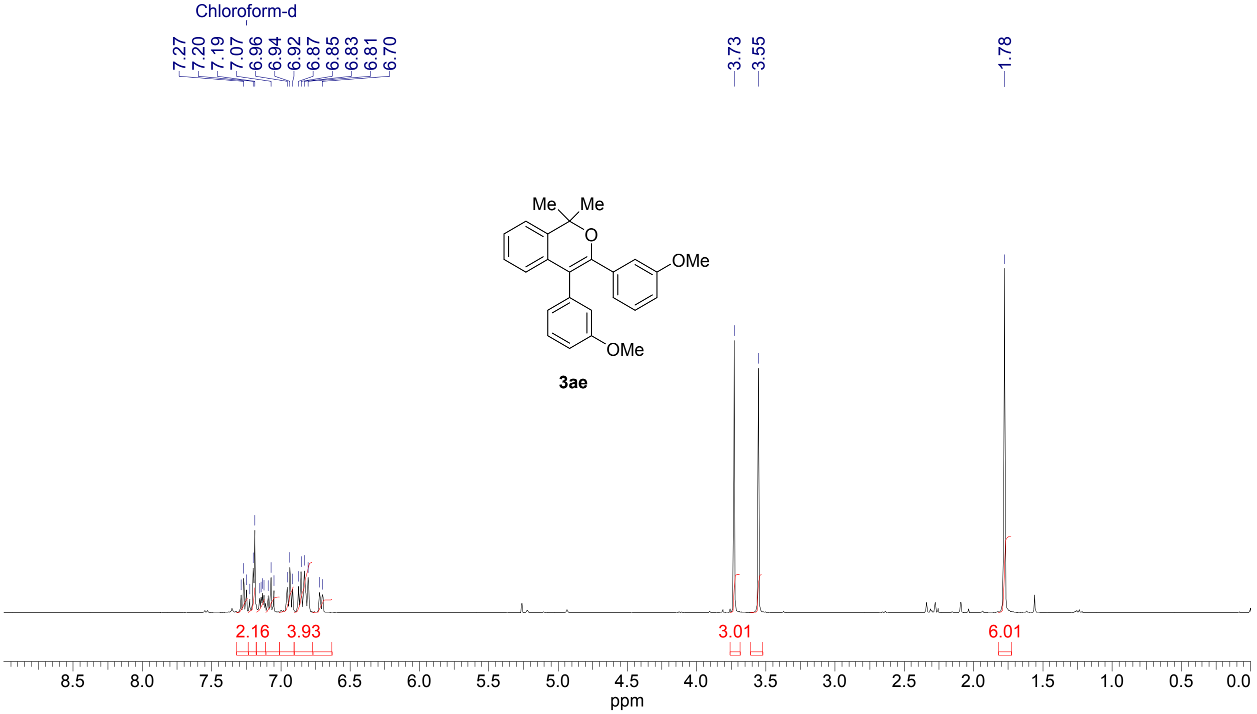

^{1}H-NMR (400 MHz) spectrum of **3ae** in $CDCl_3$

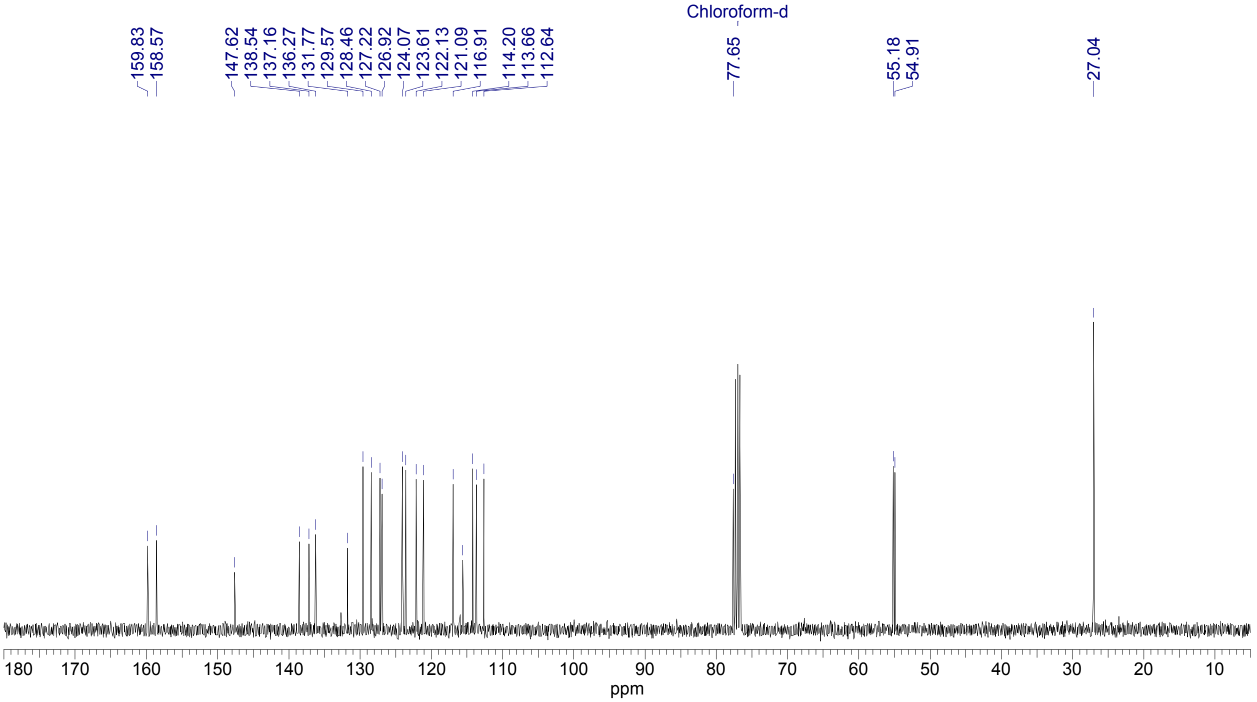

^{13}C{^{1}H}-NMR (100 MHz) spectrum of **3ae** in $CDCl_3$

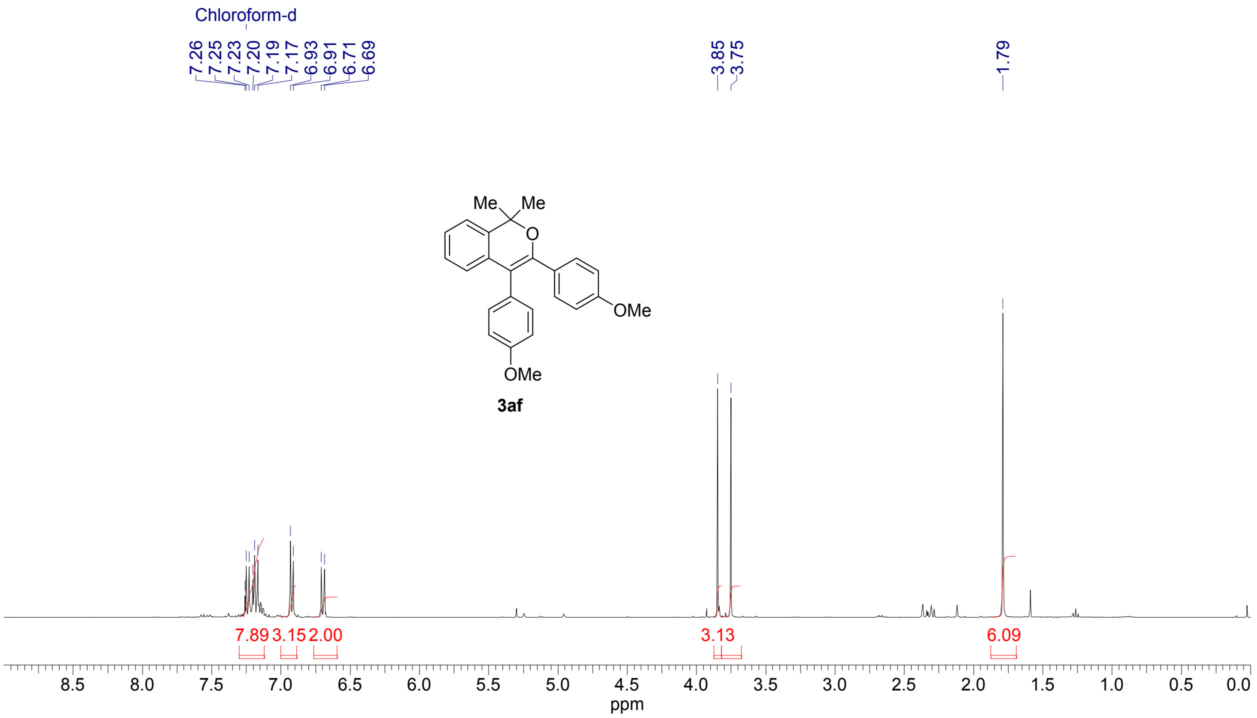

^{1}H-NMR (400 MHz) spectrum of **3af** in $CDCl_3$

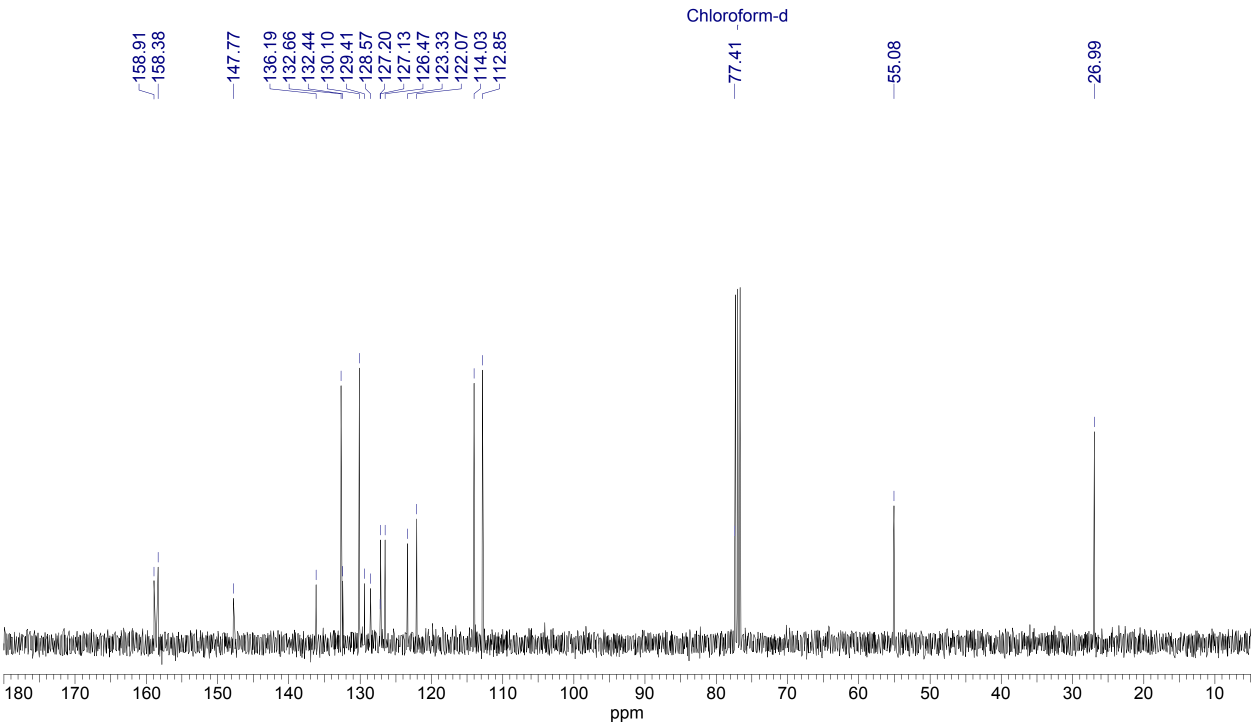

$^{13}C\{^{1}H\}$-NMR (100 MHz) spectrum of **3af** in $CDCl_3$

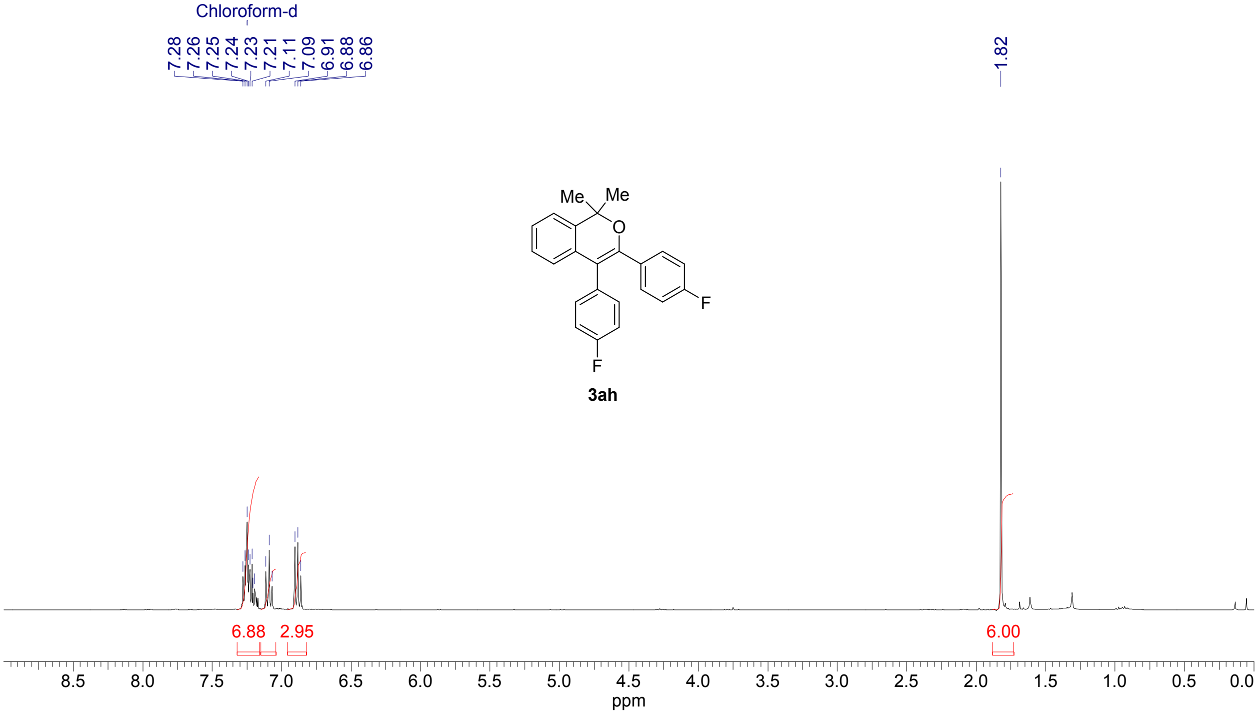

^{1}H-NMR (400 MHz) spectrum of **3ah** in $CDCl_3$

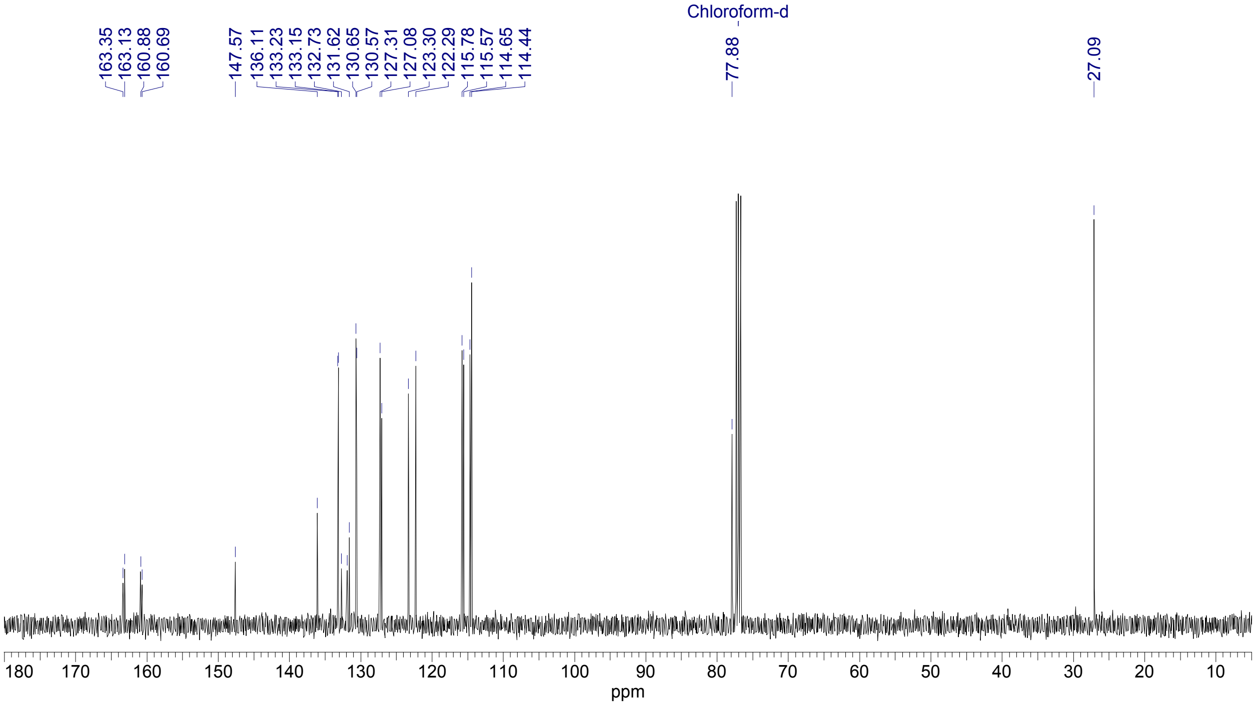

^{13}C{^{1}H}-NMR (100 MHz) spectrum of **3ah** in $CDCl_3$

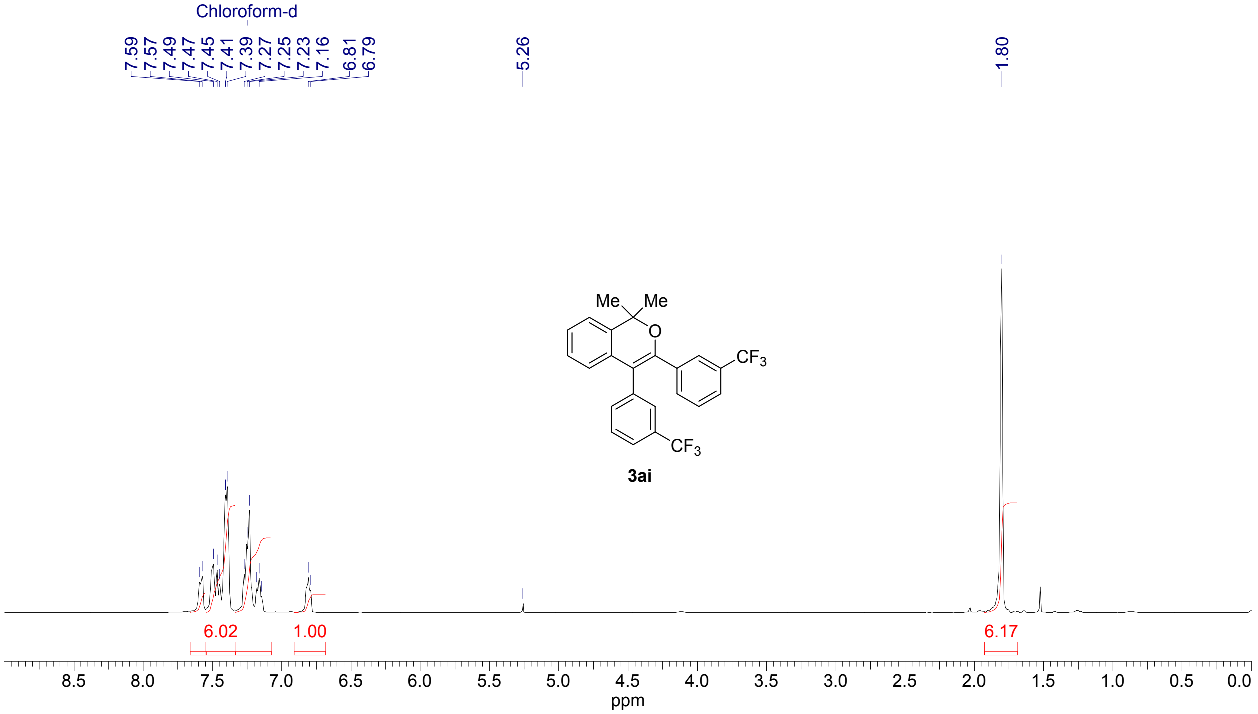

^{1}H-NMR (400 MHz) spectrum of **3ai** in $CDCl_3$

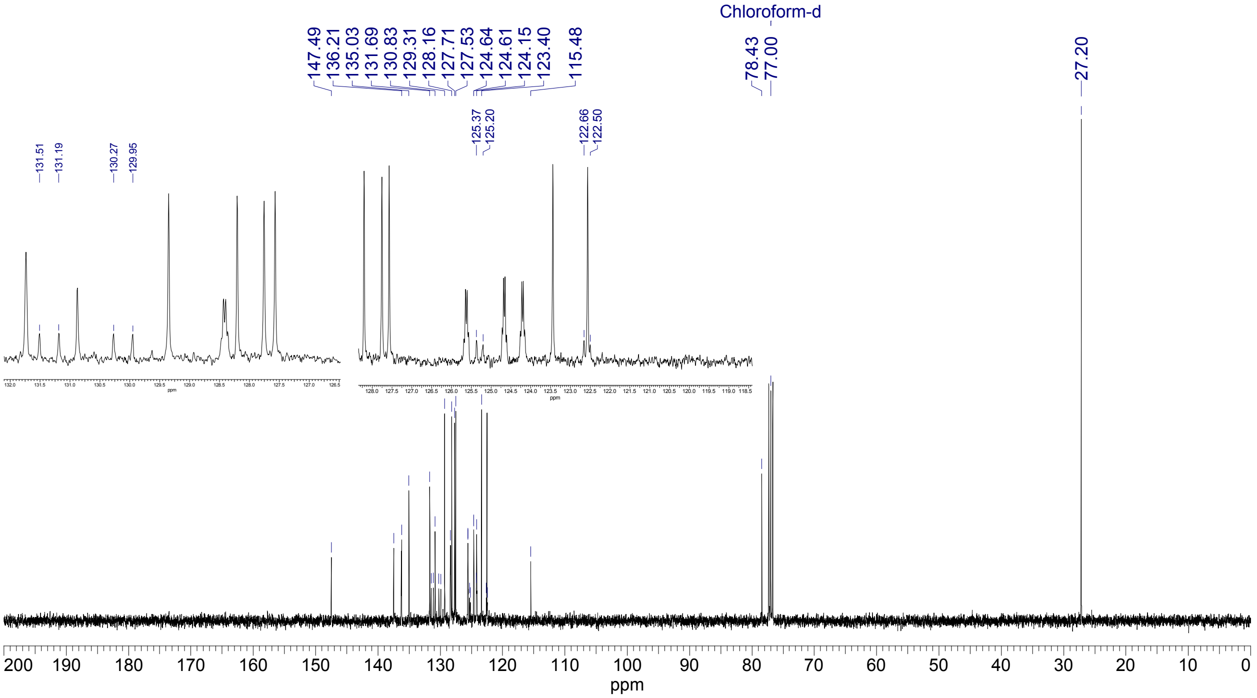

^{13}C{^{1}H}-NMR (100 MHz) spectrum of **3ai** in $CDCl_3$

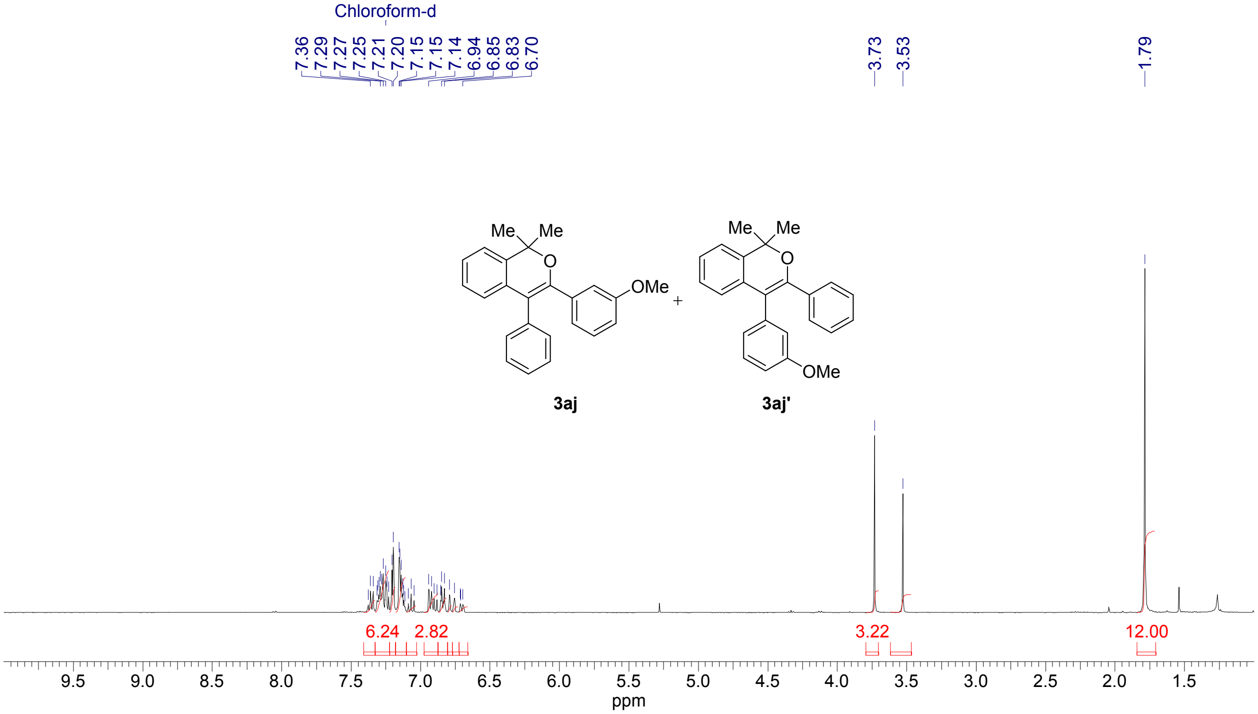

^{1}H-NMR (400 MHz) spectrum of **3aj** and **3aj'** in $CDCl_3$

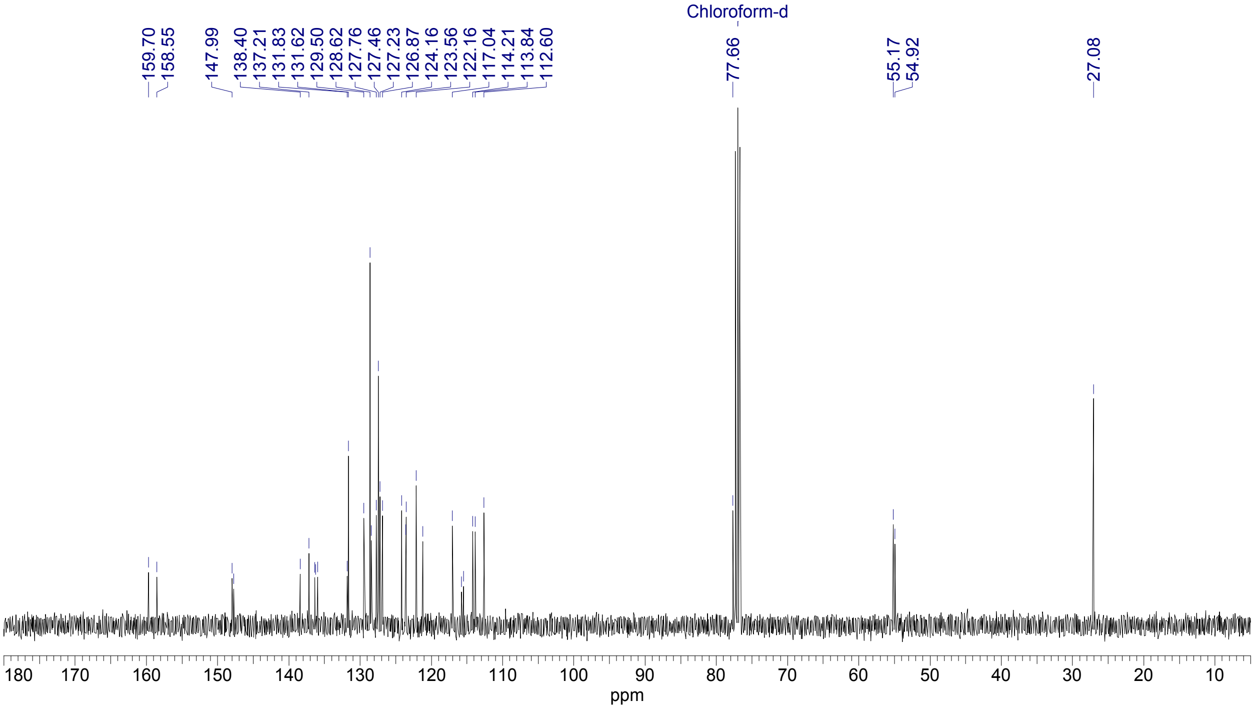

^{13}C{^{1}H}-NMR (100 MHz) spectrum of **3aj** and **3aj'** in $CDCl_3$

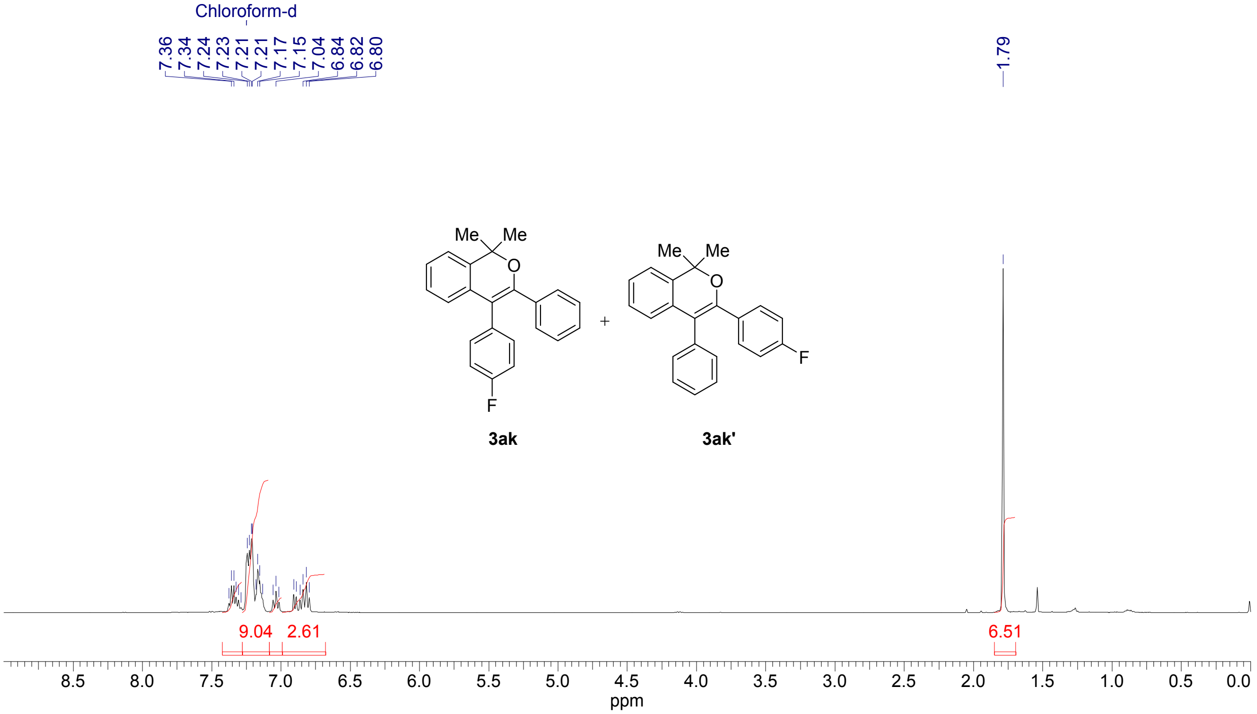

^{1}H-NMR (400 MHz) spectrum of **3ak** and **3ak'** in $CDCl_3$

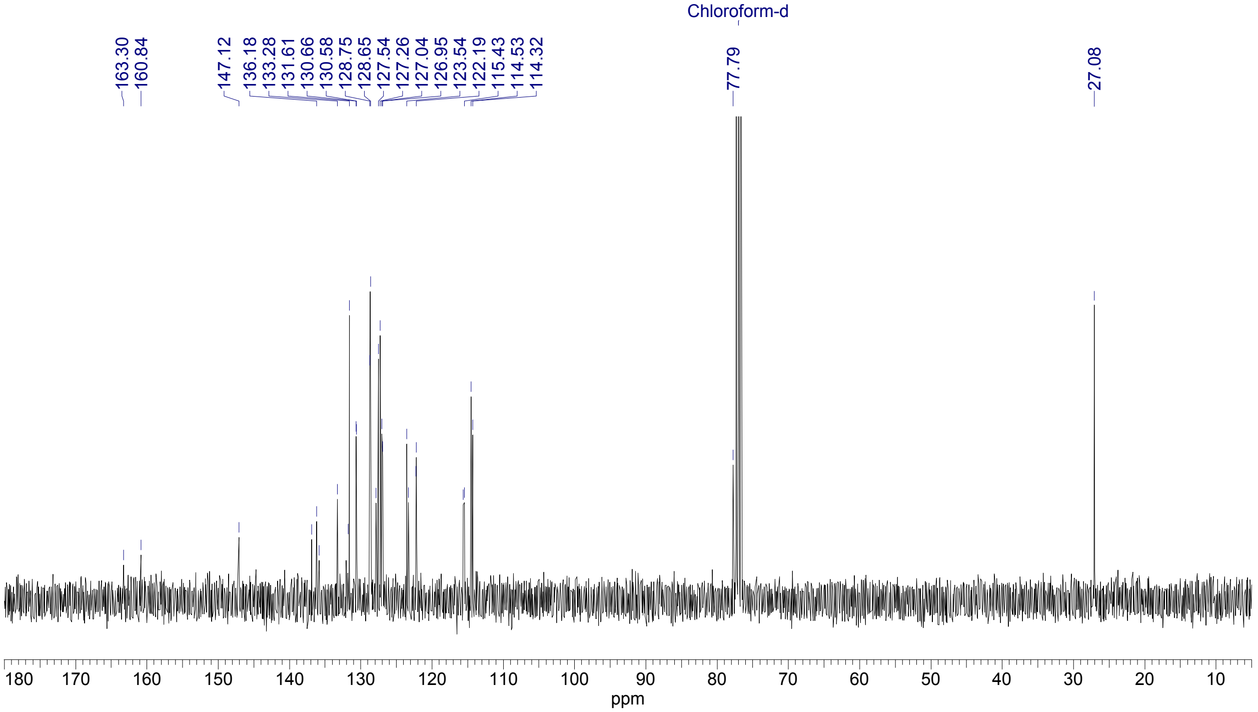

^{13}C{^{1}H}-NMR (100 MHz) spectrum of **3ak** and **3ak'** in $CDCl_3$

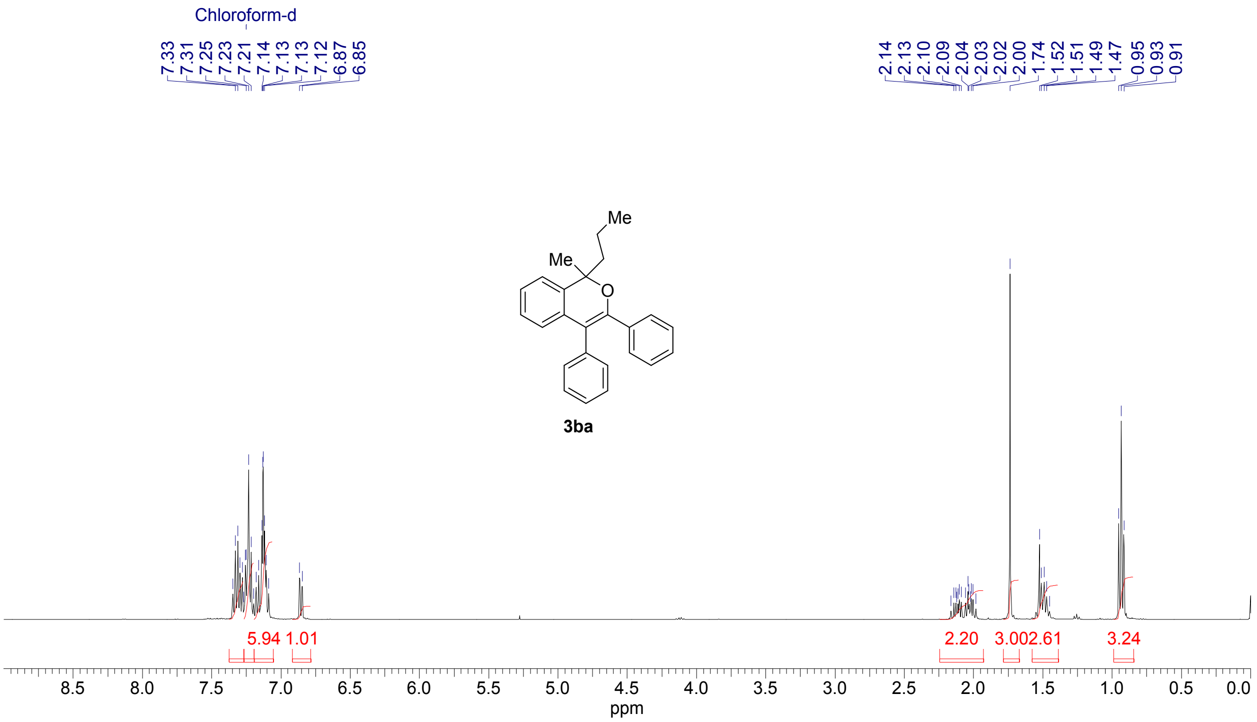

^{1}H-NMR (400 MHz) spectrum of **3ba** in $CDCl_3$

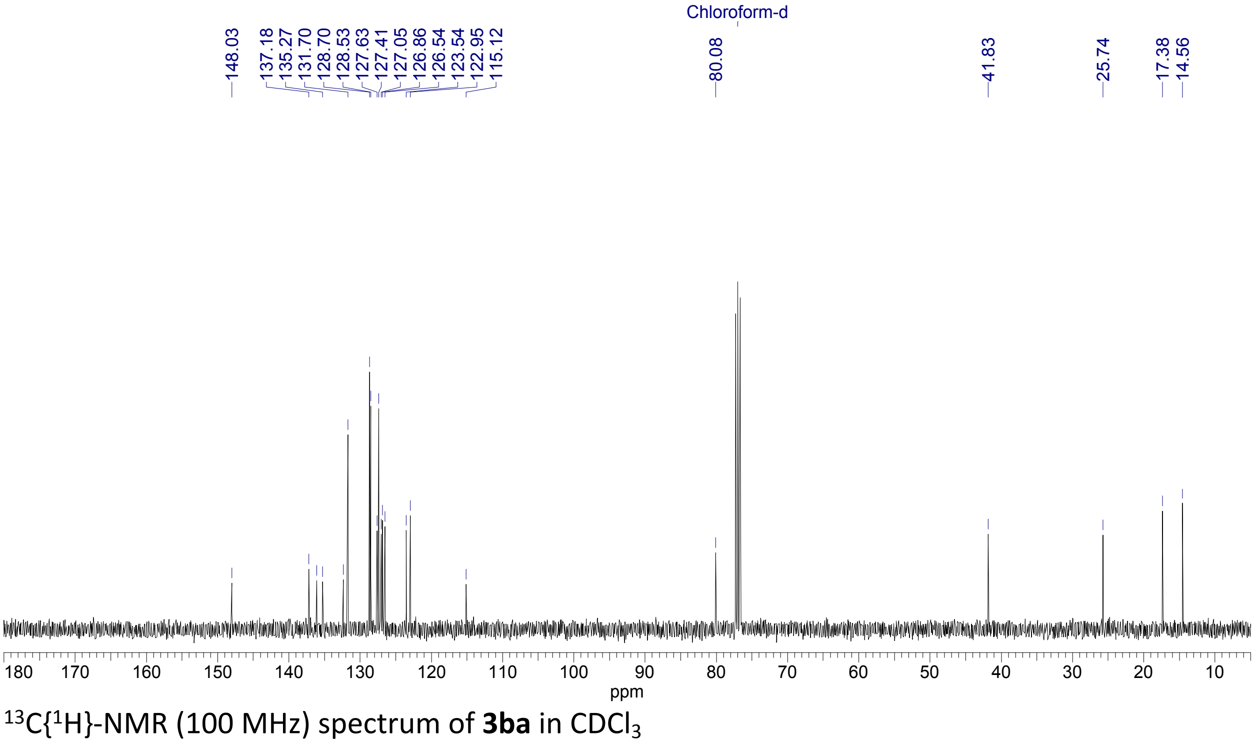

$^{13}C\{^{1}H\}$-NMR (100 MHz) spectrum of **3ba** in $CDCl_3$

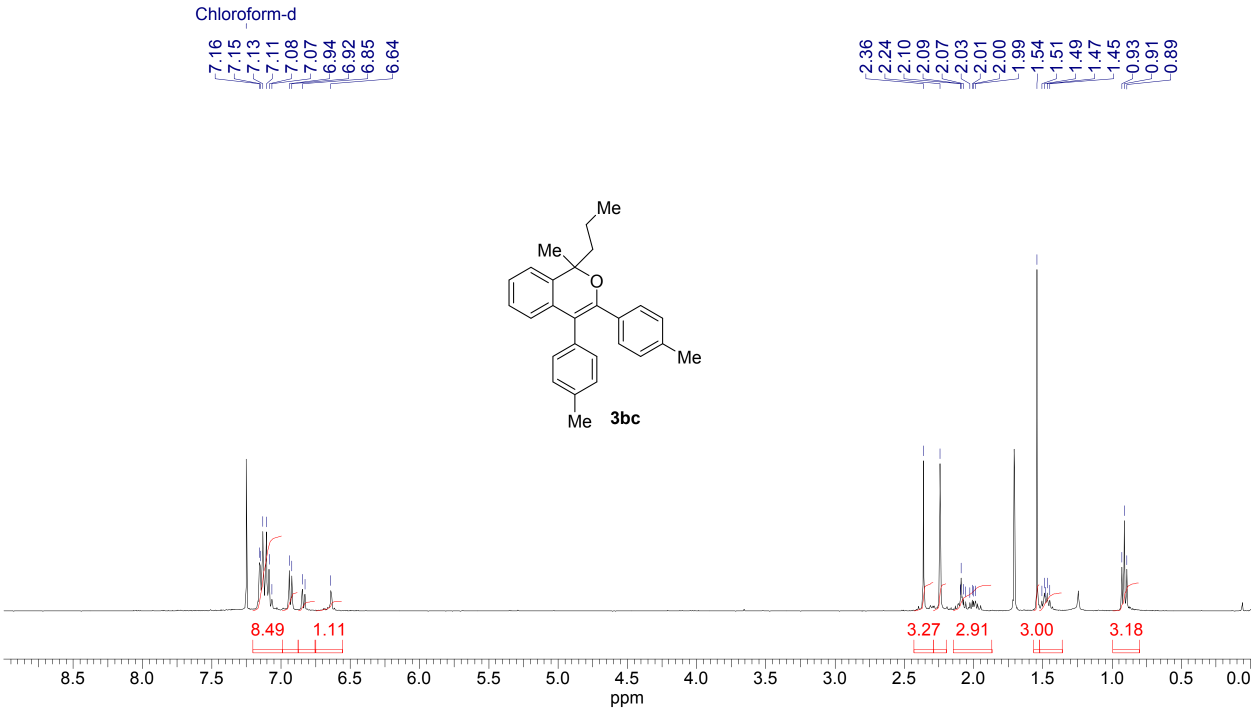

^{1}H-NMR (400 MHz) spectrum of **3bc** in $CDCl_3$

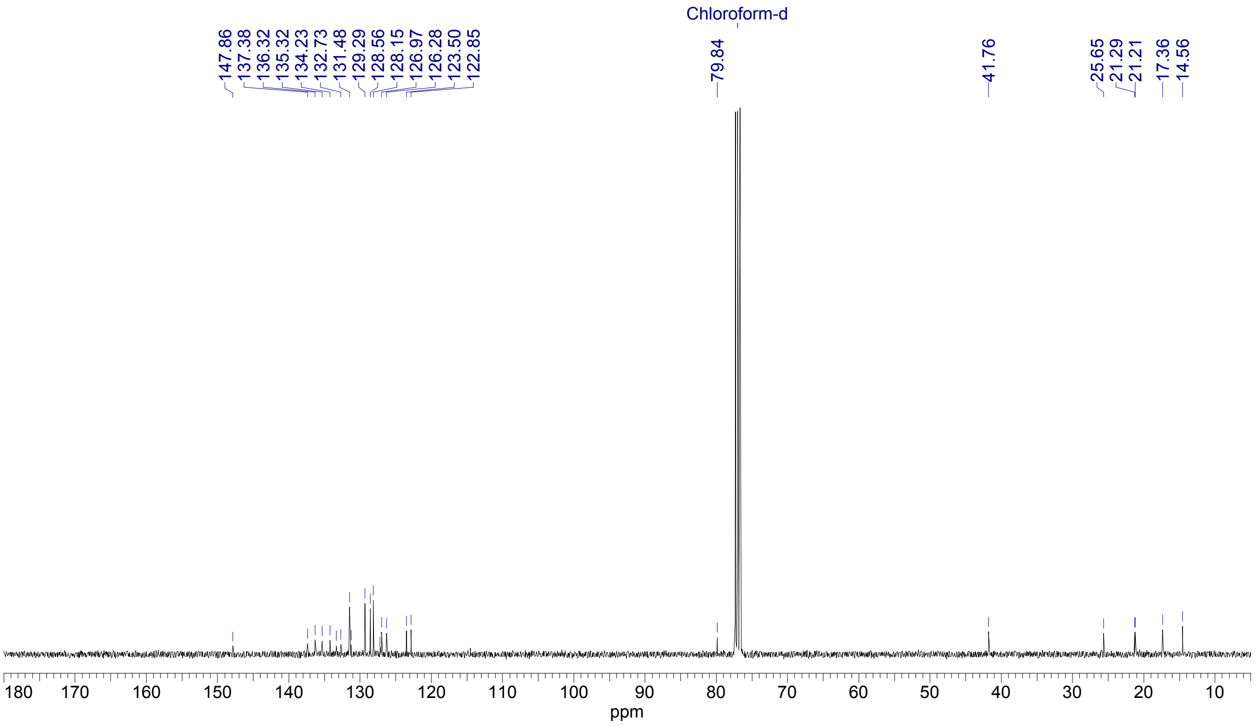

^{13}C{^{1}H}-NMR (100 MHz) spectrum of **3bc** in $CDCl_3$

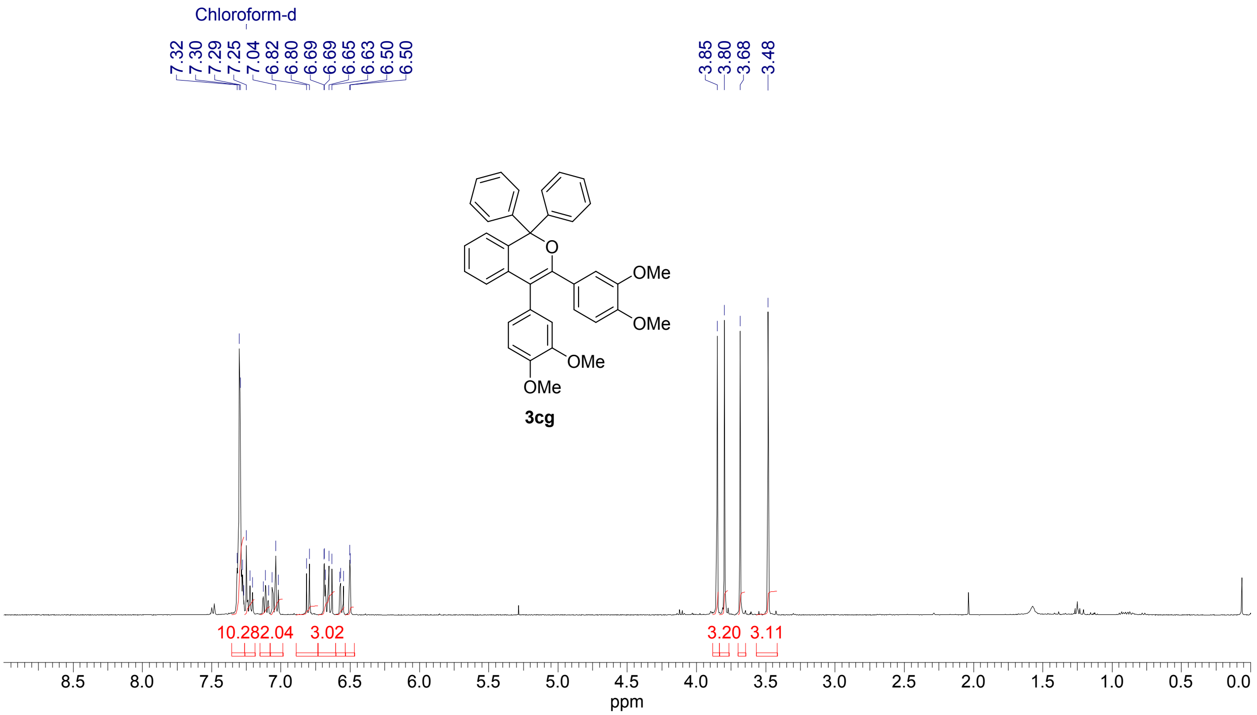

^{1}H-NMR (400 MHz) spectrum of **3cg** in $CDCl_3$

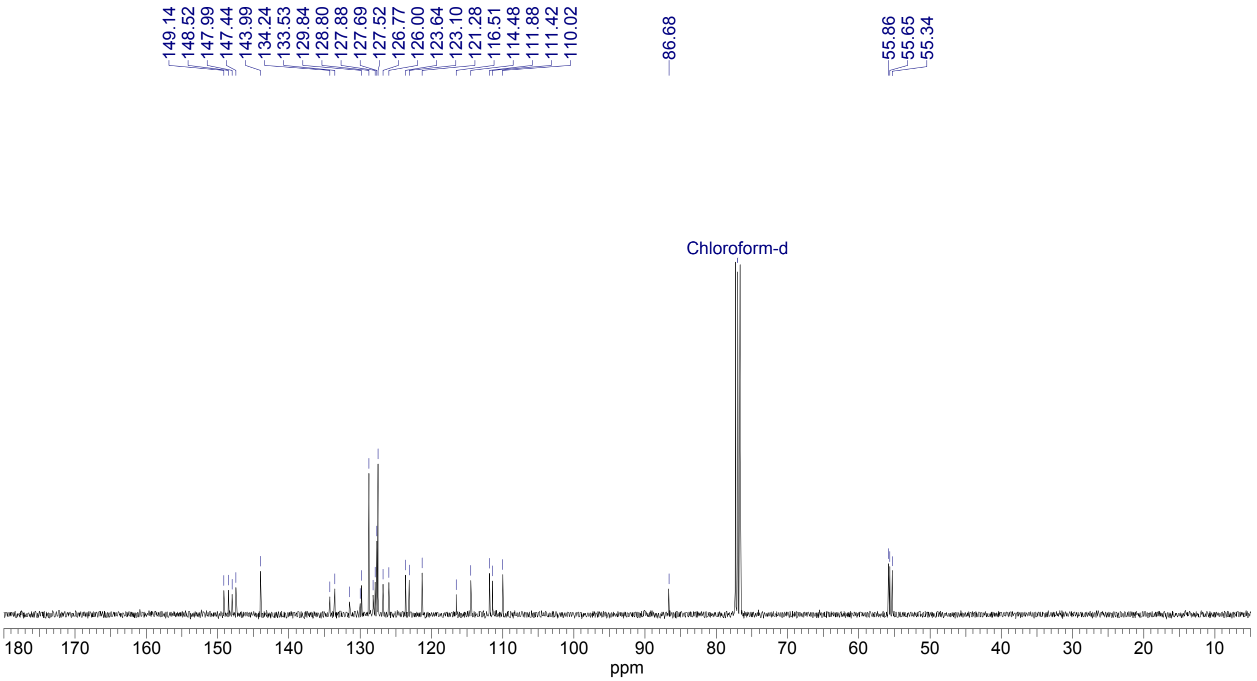

^{13}C{^{1}H}-NMR (100 MHz) spectrum of **3cg** in $CDCl_3$

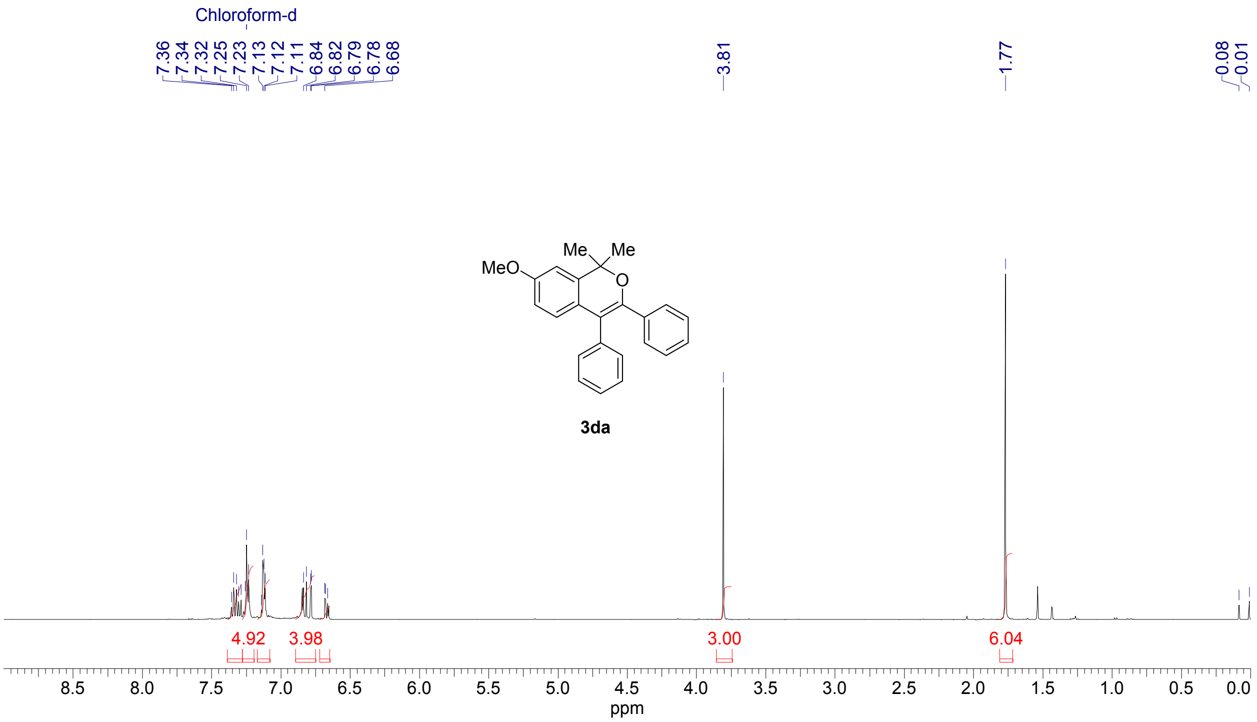

^{1}H-NMR (400 MHz) spectrum of **3da** in $CDCl_3$

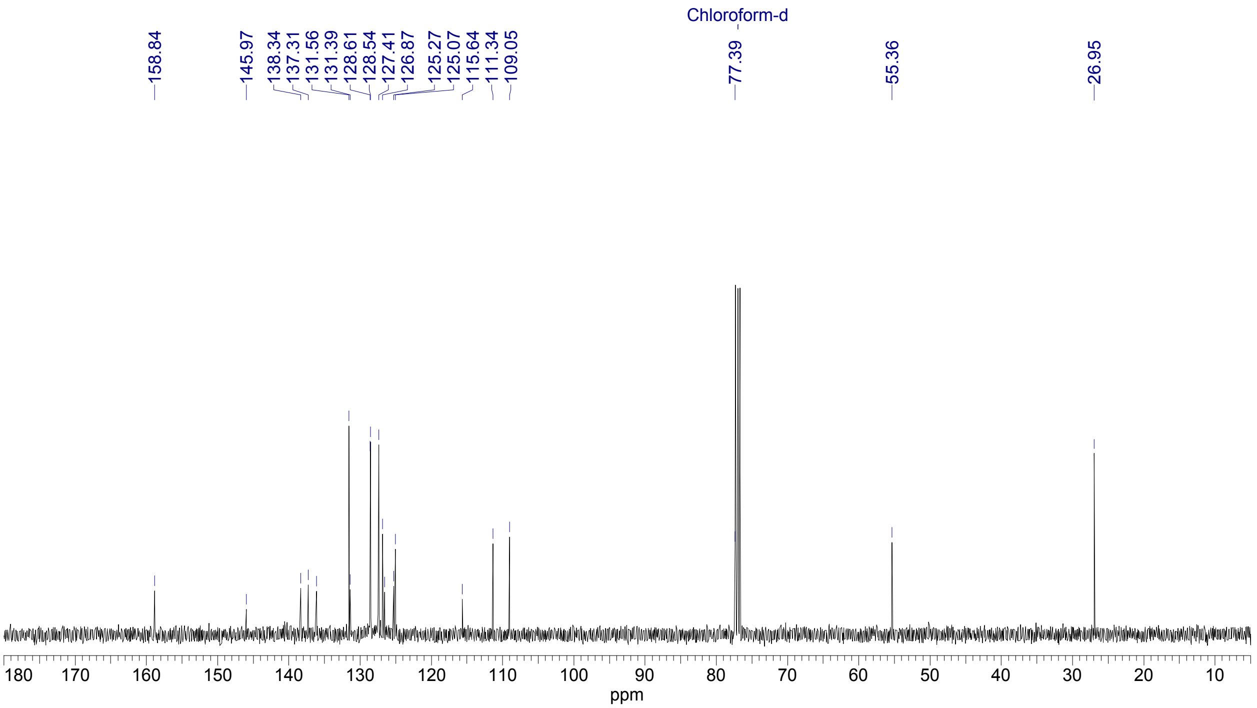

$^{13}C\{^{1}H\}$-NMR (100 MHz) spectrum of **3da** in $CDCl_3$

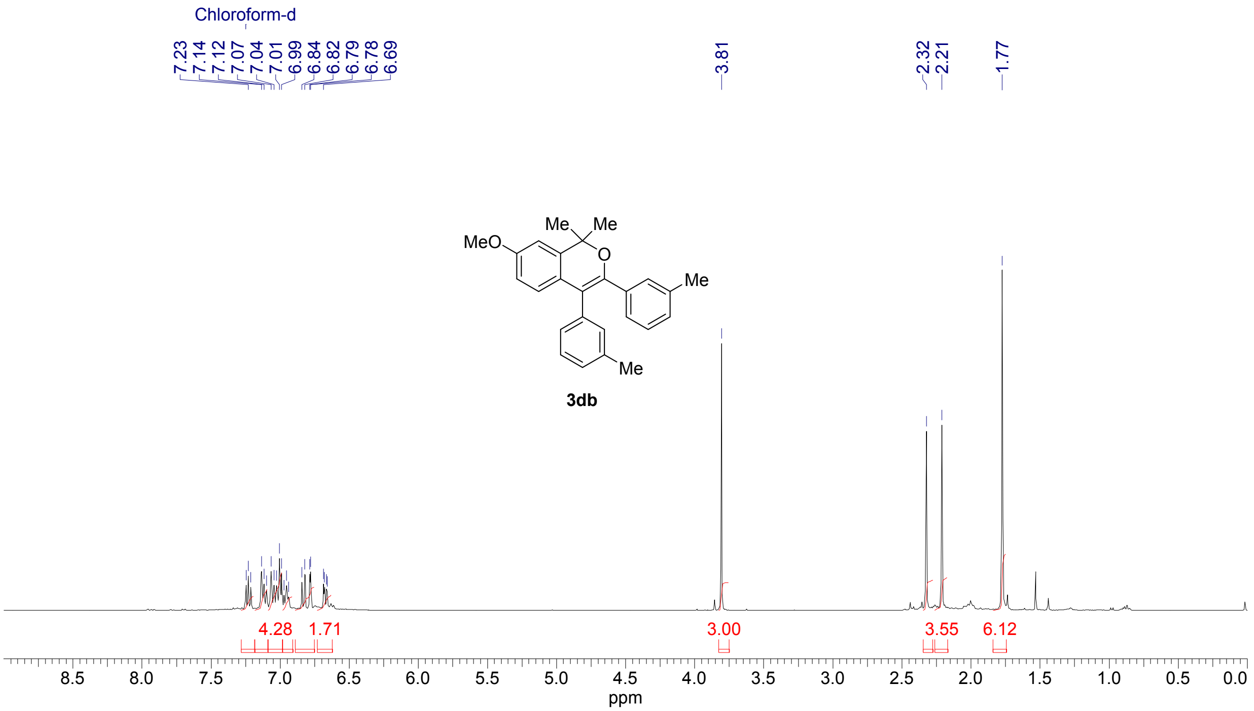

^{1}H-NMR (400 MHz) spectrum of **3db** in $CDCl_3$

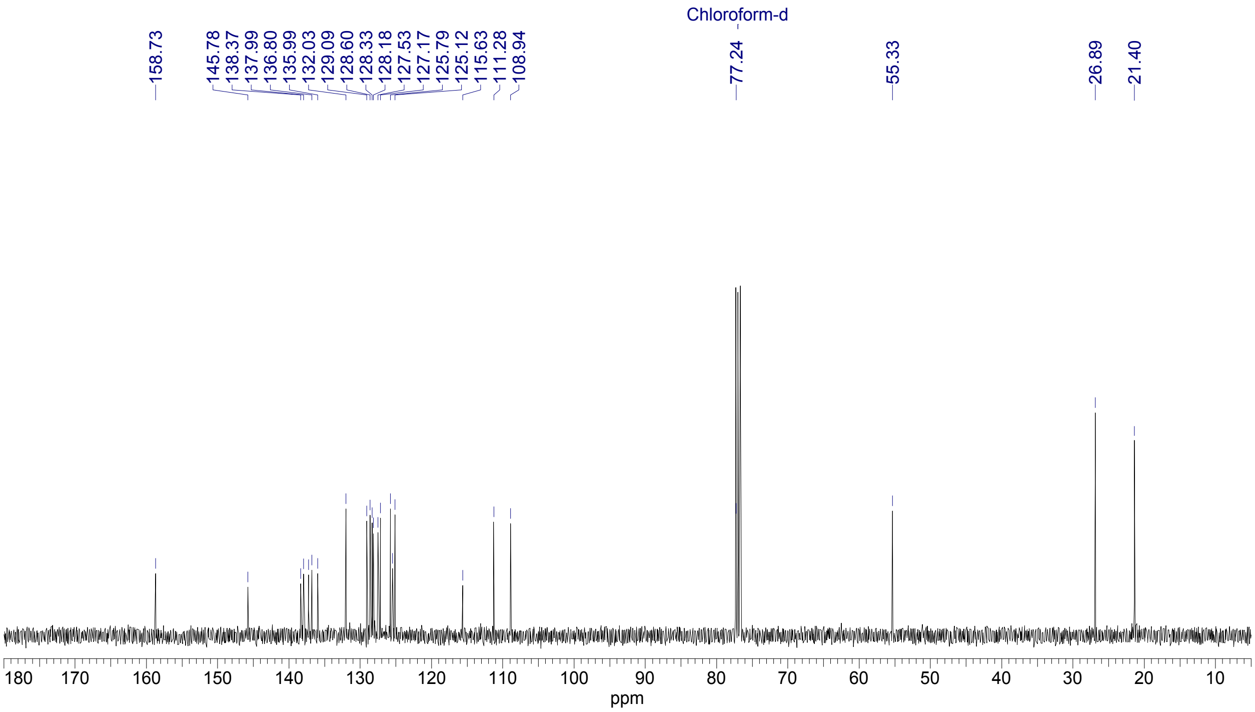

^{13}C{^{1}H}-NMR (100 MHz) spectrum of **3db** in $CDCl_3$

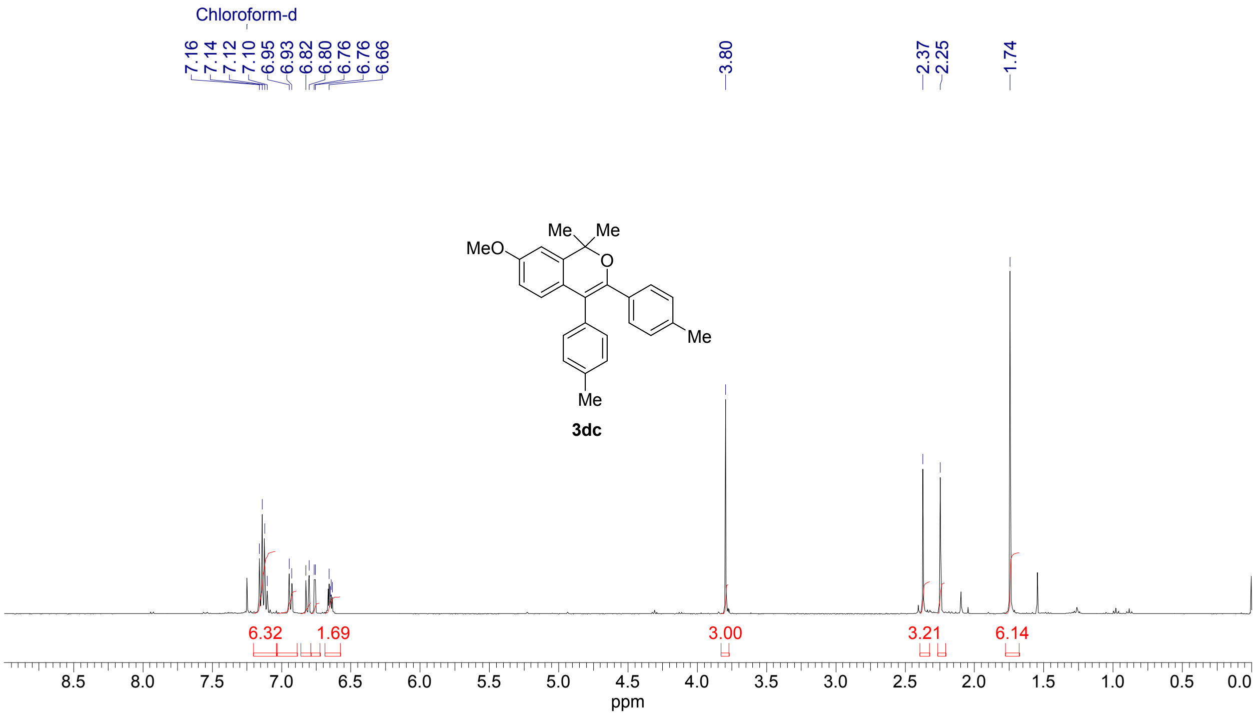

^{1}H-NMR (400 MHz) spectrum of **3dc** in $CDCl_3$

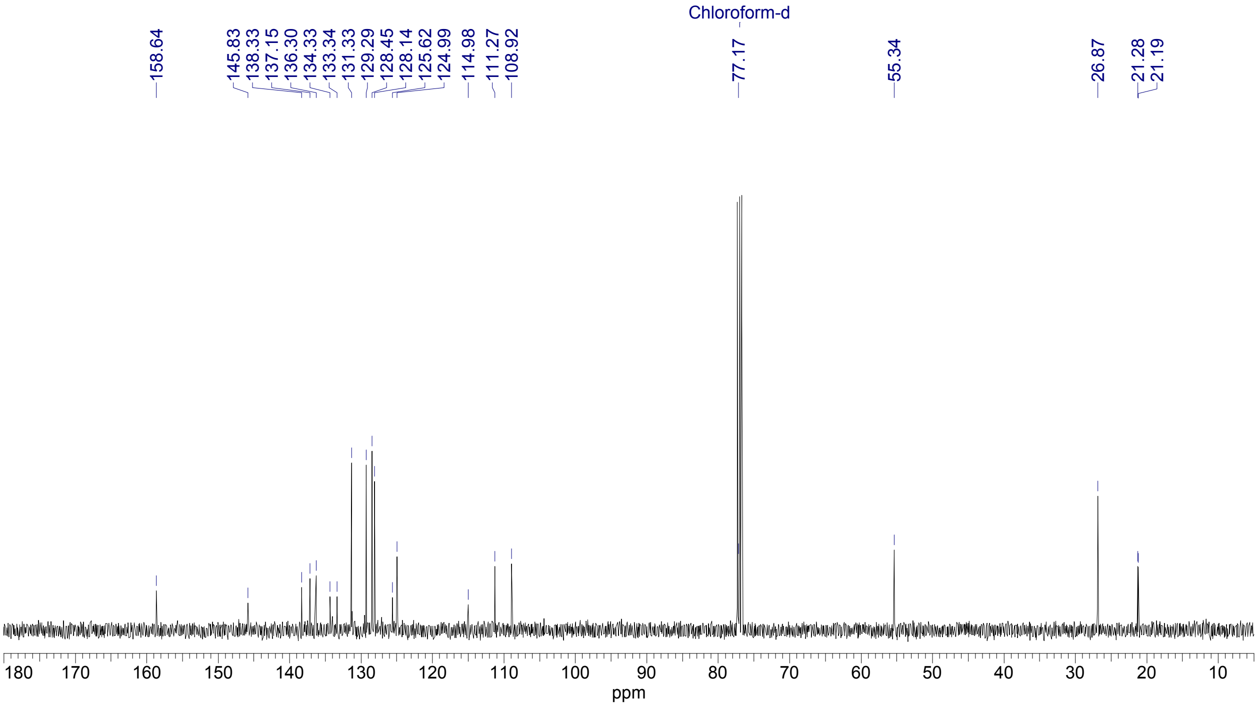

$^{13}C\{^1H\}$-NMR (100 MHz) spectrum of **3dc** in $CDCl_3$

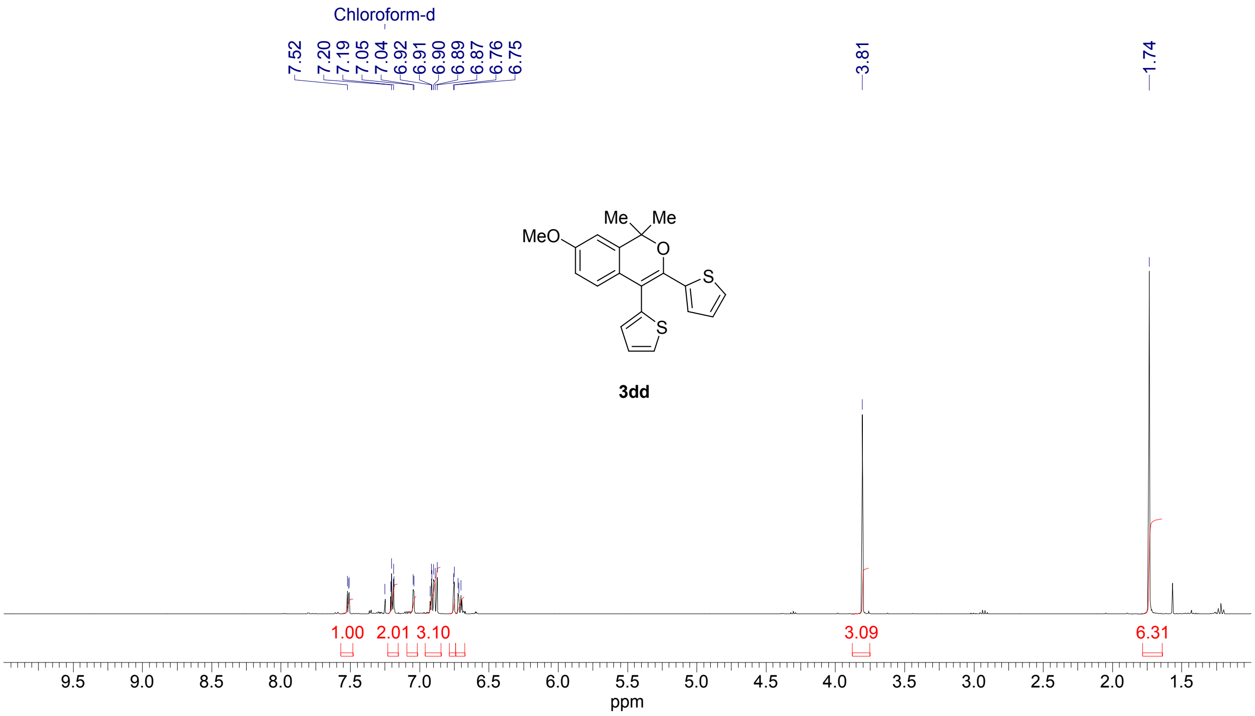

^{1}H-NMR (400 MHz) spectrum of **3dd** in $CDCl_3$

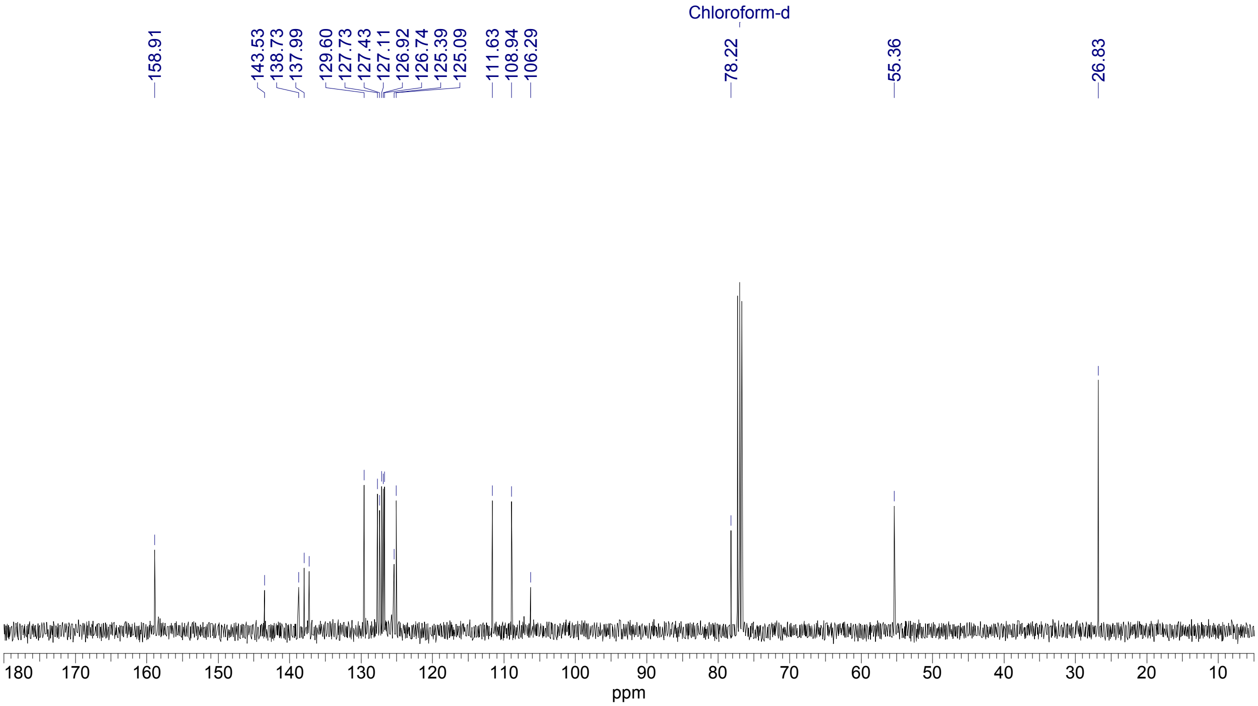

$^{13}C\{^1H\}$-NMR (100 MHz) spectrum of **3dd** in $CDCl_3$

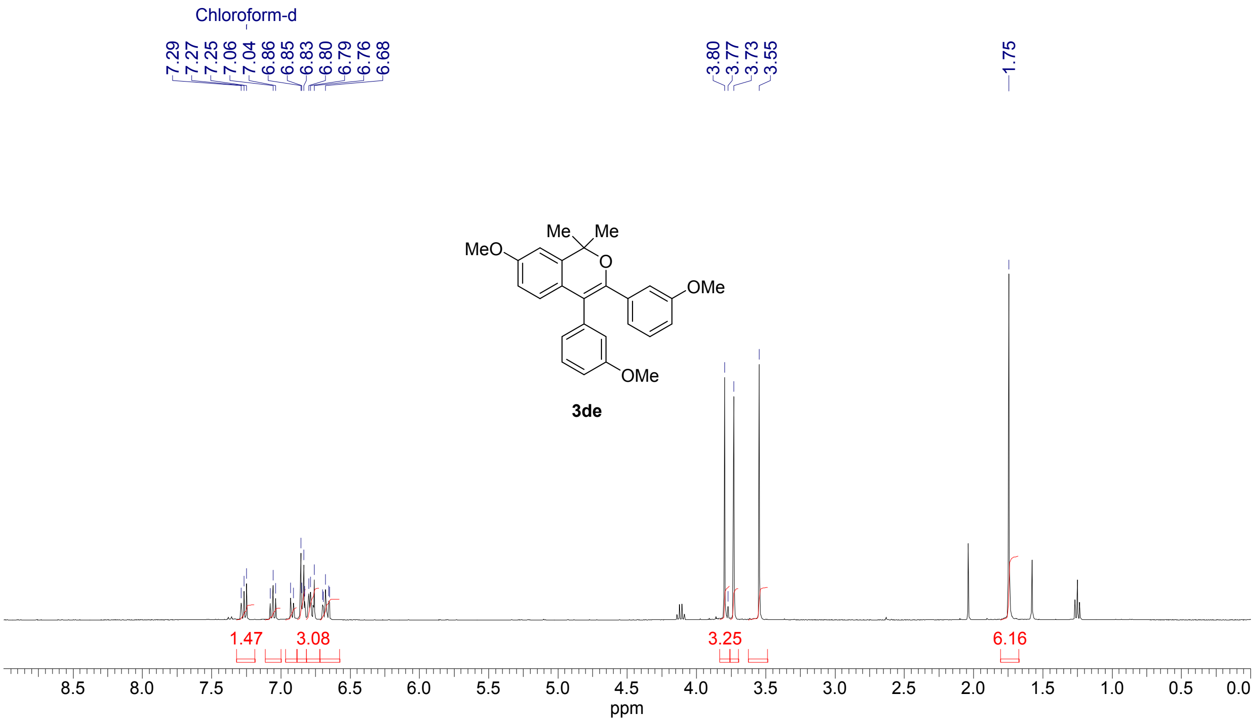

^{1}H-NMR (400 MHz) spectrum of **3de** in $CDCl_3$

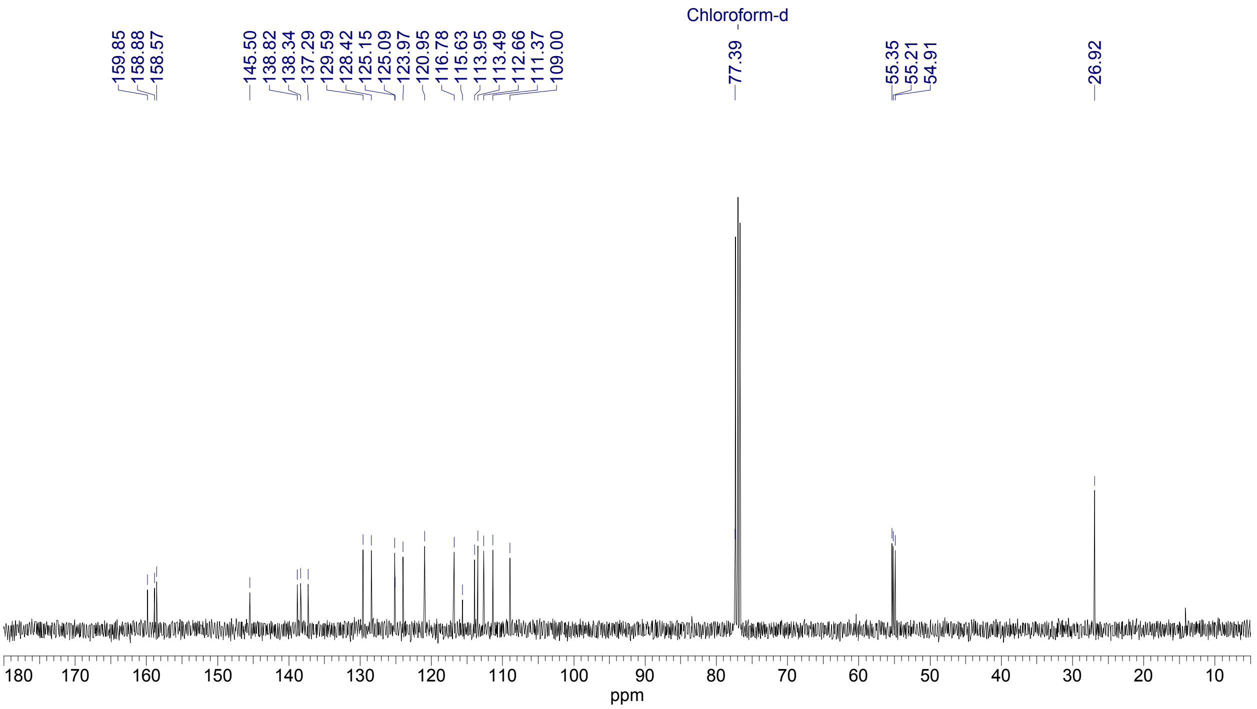

$^{13}C\{^{1}H\}$-NMR (100 MHz) spectrum of **3de** in $CDCl_3$

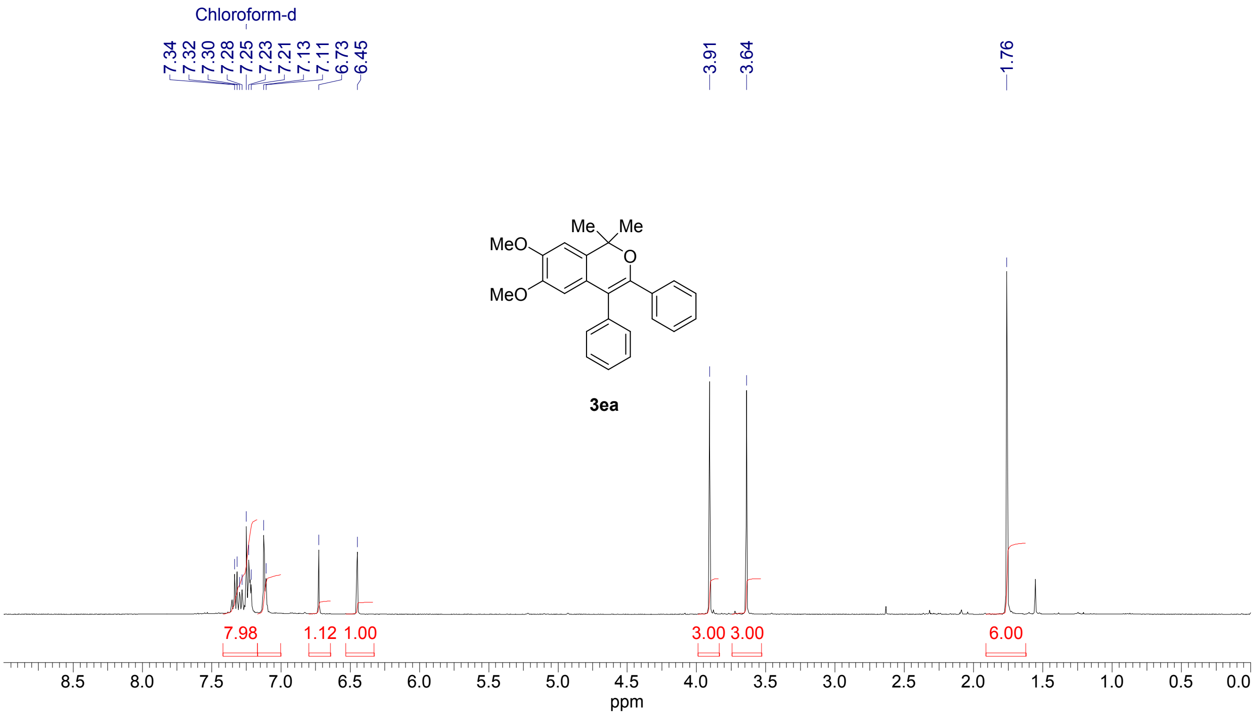

^{1}H-NMR (400 MHz) spectrum of **3ea** in $CDCl_3$

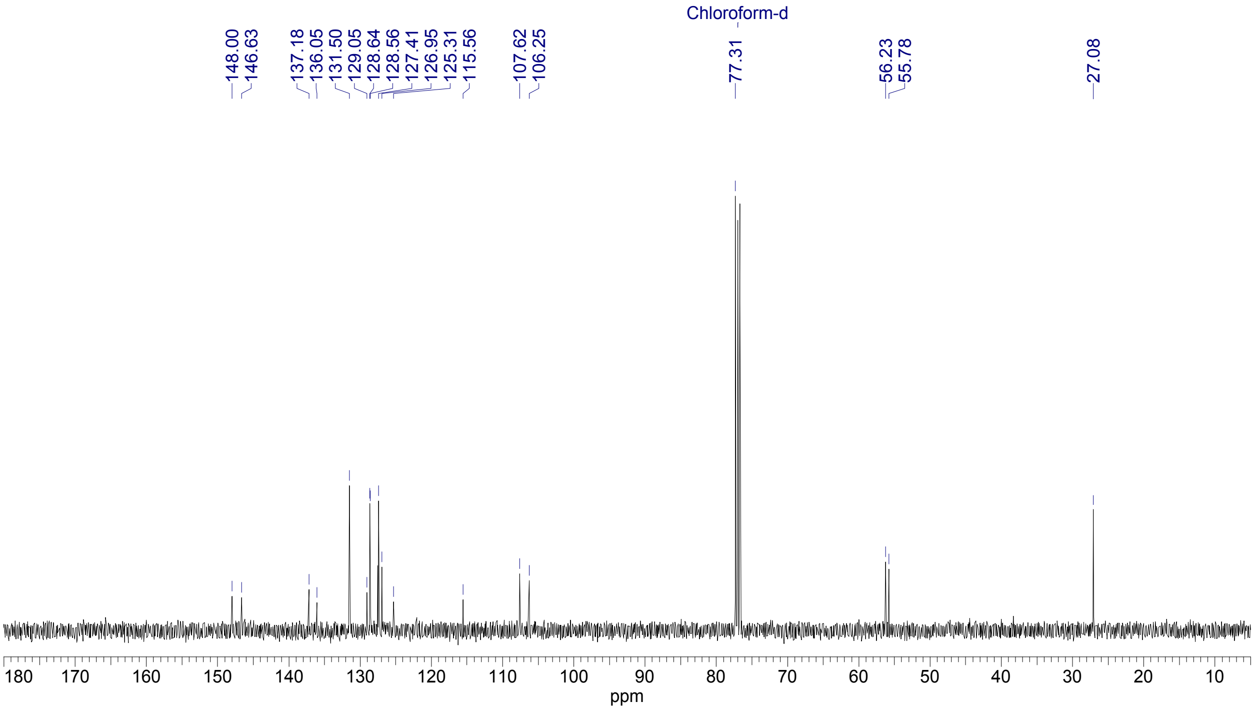

^{13}C{^{1}H}-NMR (100 MHz) spectrum of **3ea** in $CDCl_3$

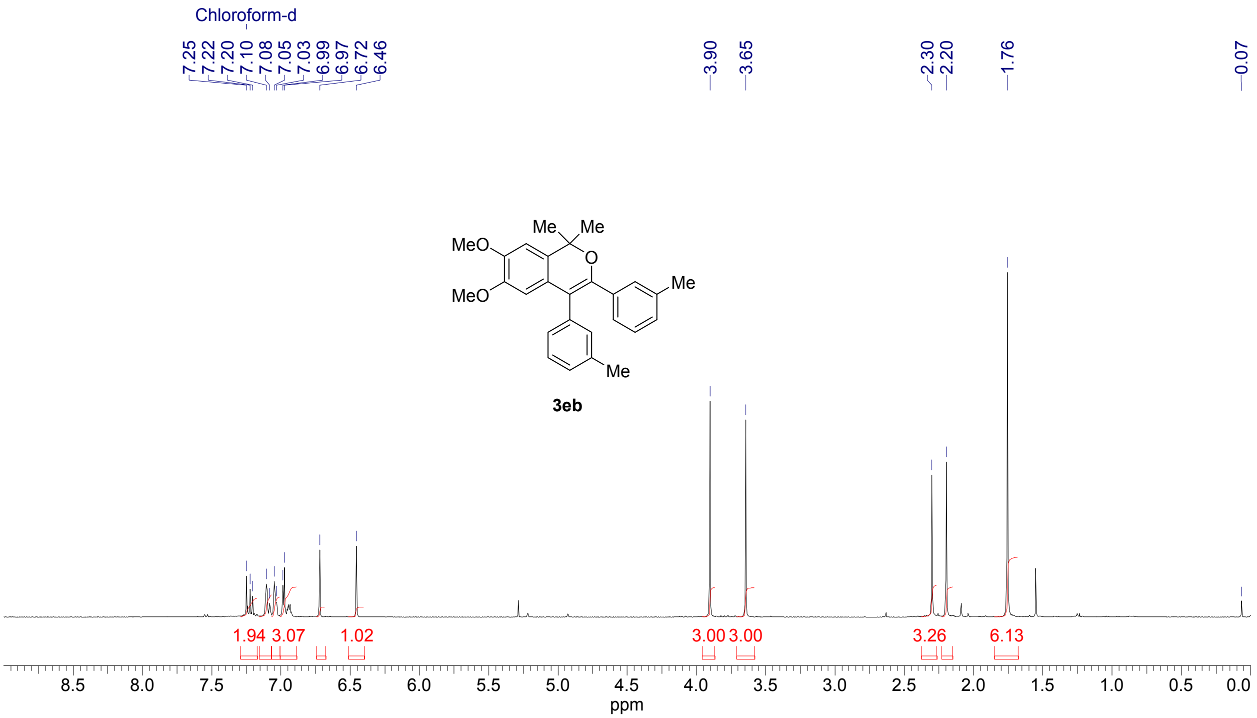

^{1}H-NMR (400 MHz) spectrum of **3eb** in $CDCl_3$

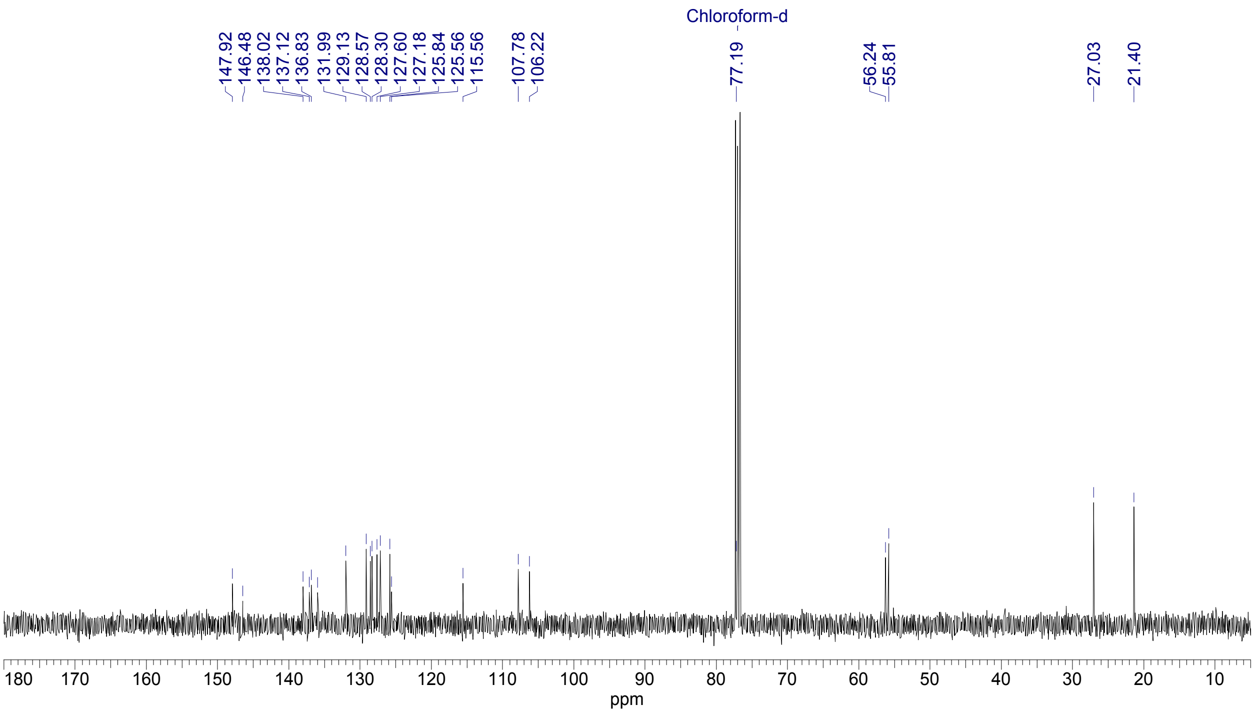

^{13}C{^{1}H}-NMR (100 MHz) spectrum of **3eb** in $CDCl_3$

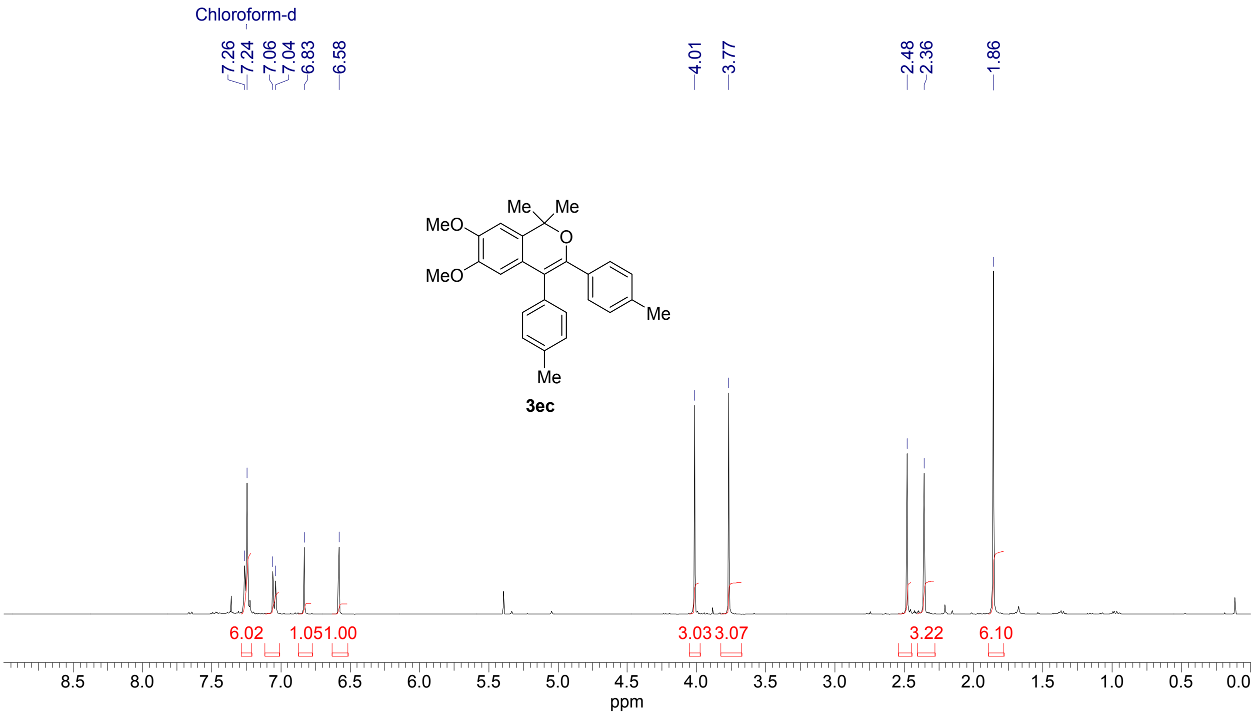

^{1}H-NMR (400 MHz) spectrum of **3ec** in $CDCl_3$

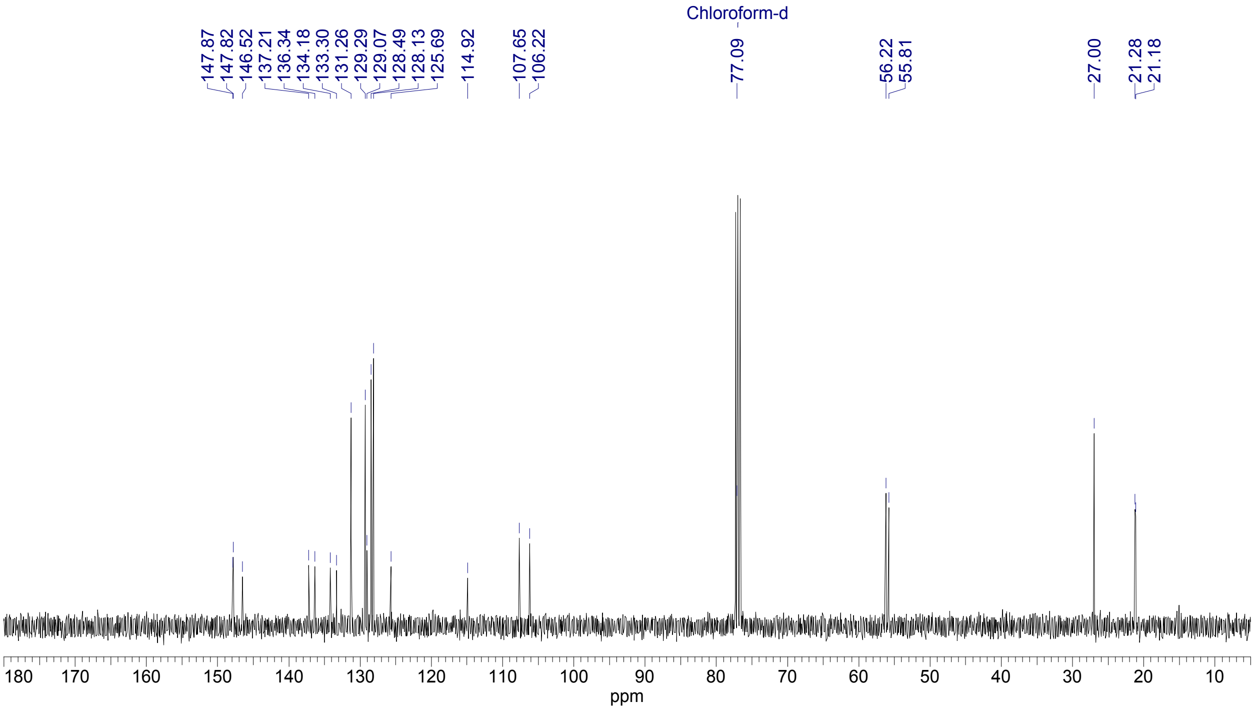

$^{13}C\{^{1}H\}$-NMR (100 MHz) spectrum of **3ec** in $CDCl_3$

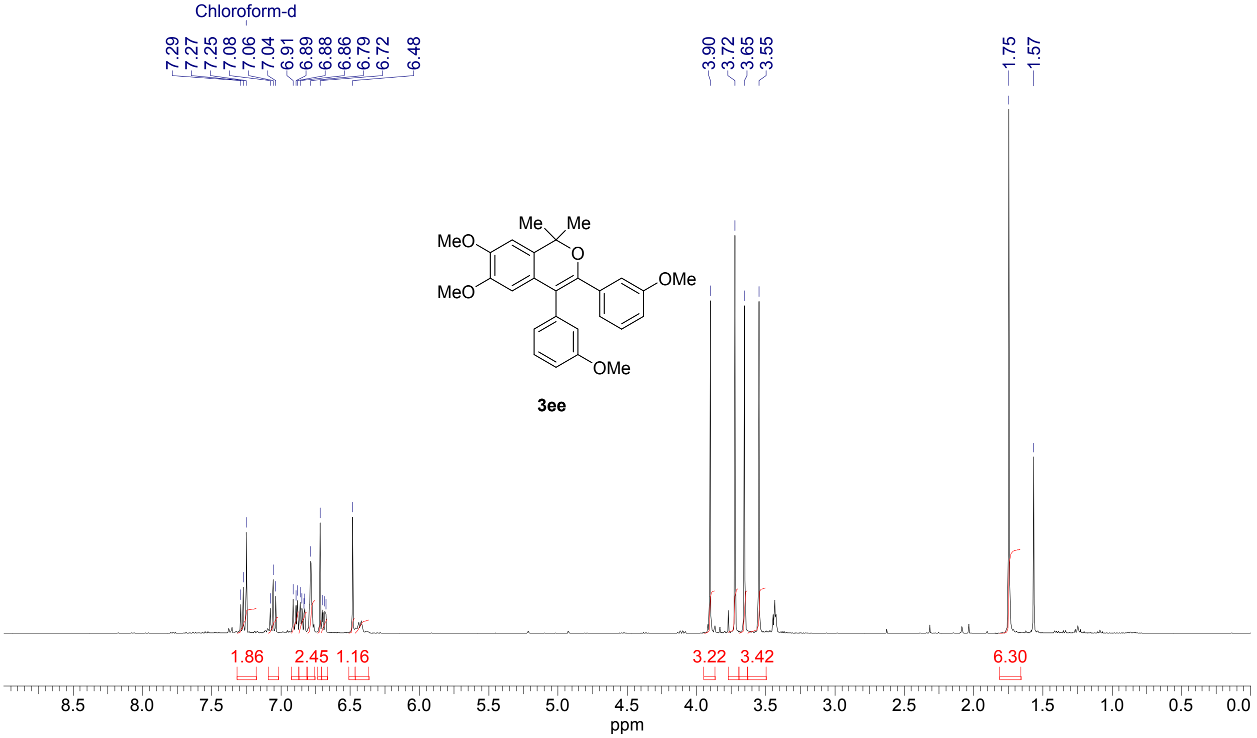

^{1}H-NMR (400 MHz) spectrum of **3ee** in $CDCl_3$

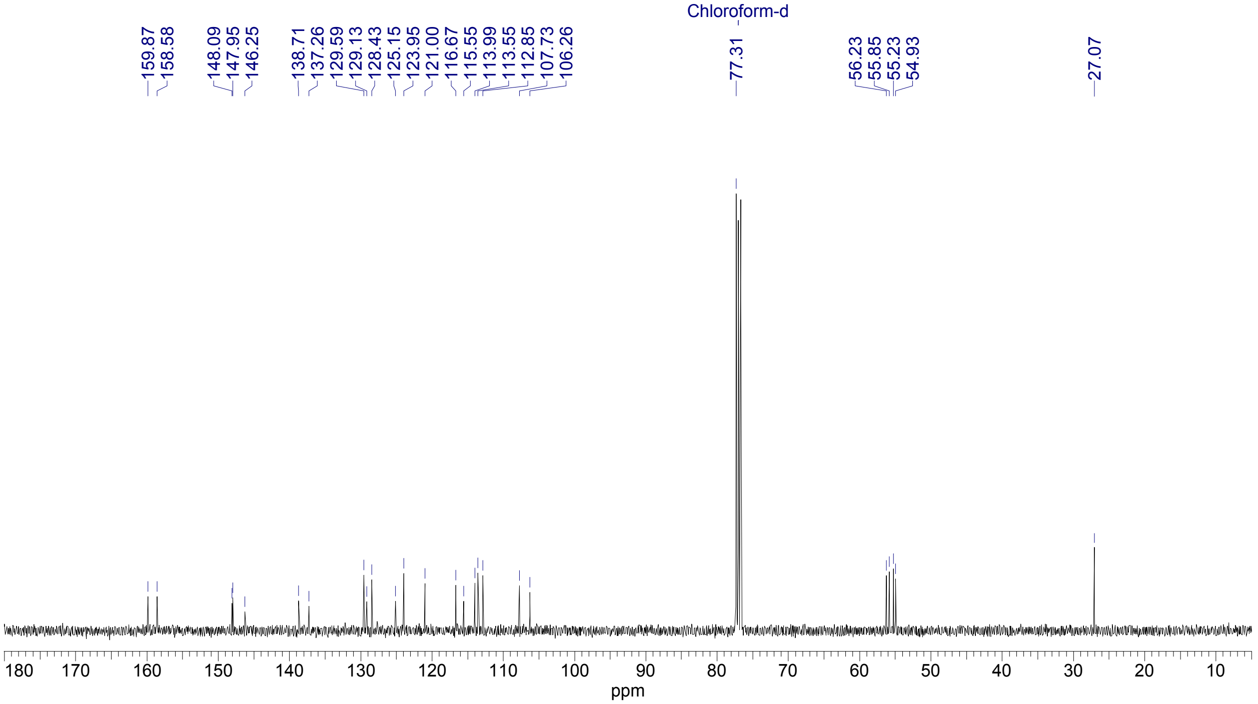

$^{13}C\{^{1}H\}$-NMR (100 MHz) spectrum of **3ee** in $CDCl_3$

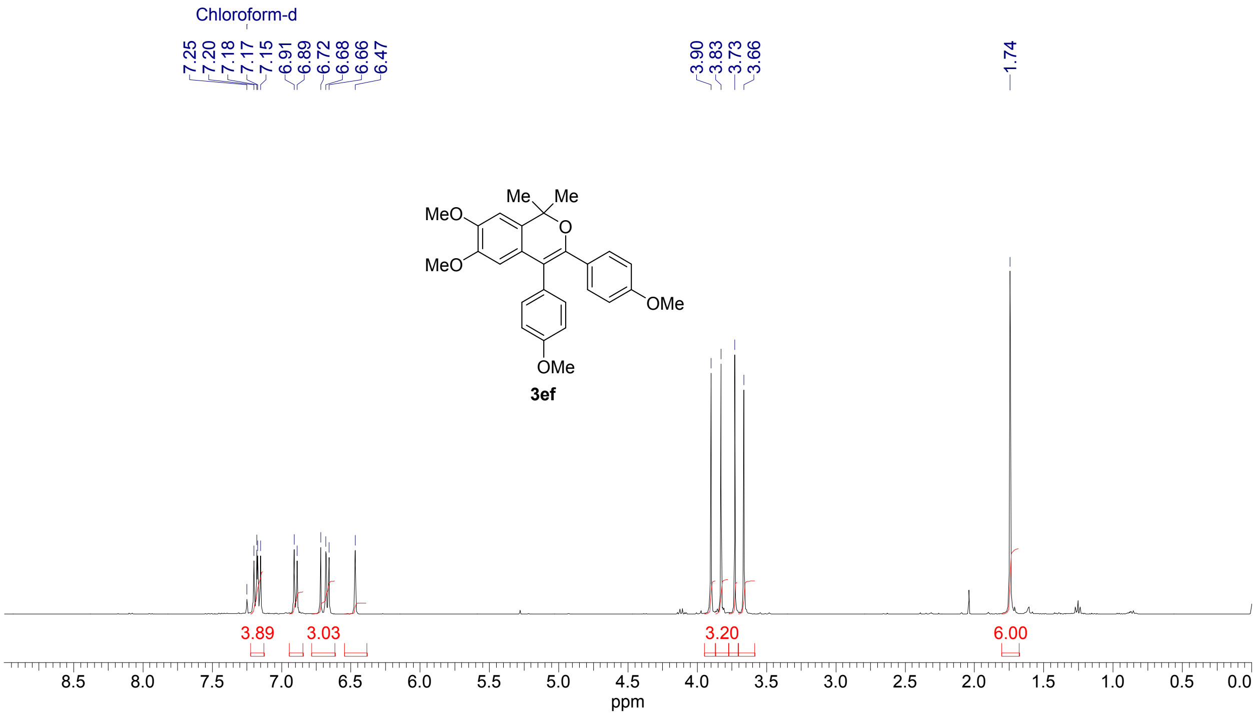

^{1}H-NMR (400 MHz) spectrum of **3ef** in $CDCl_3$

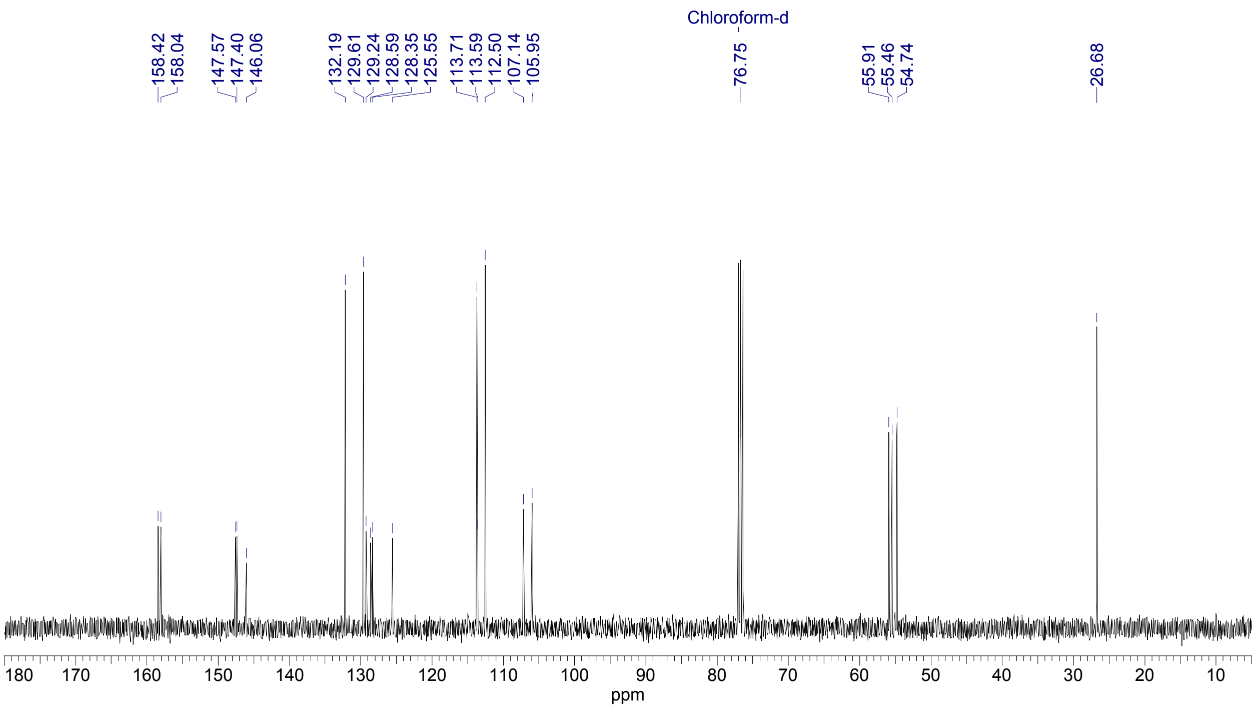

^{13}C{^{1}H}-NMR (100 MHz) spectrum of **3ef** in $CDCl_3$

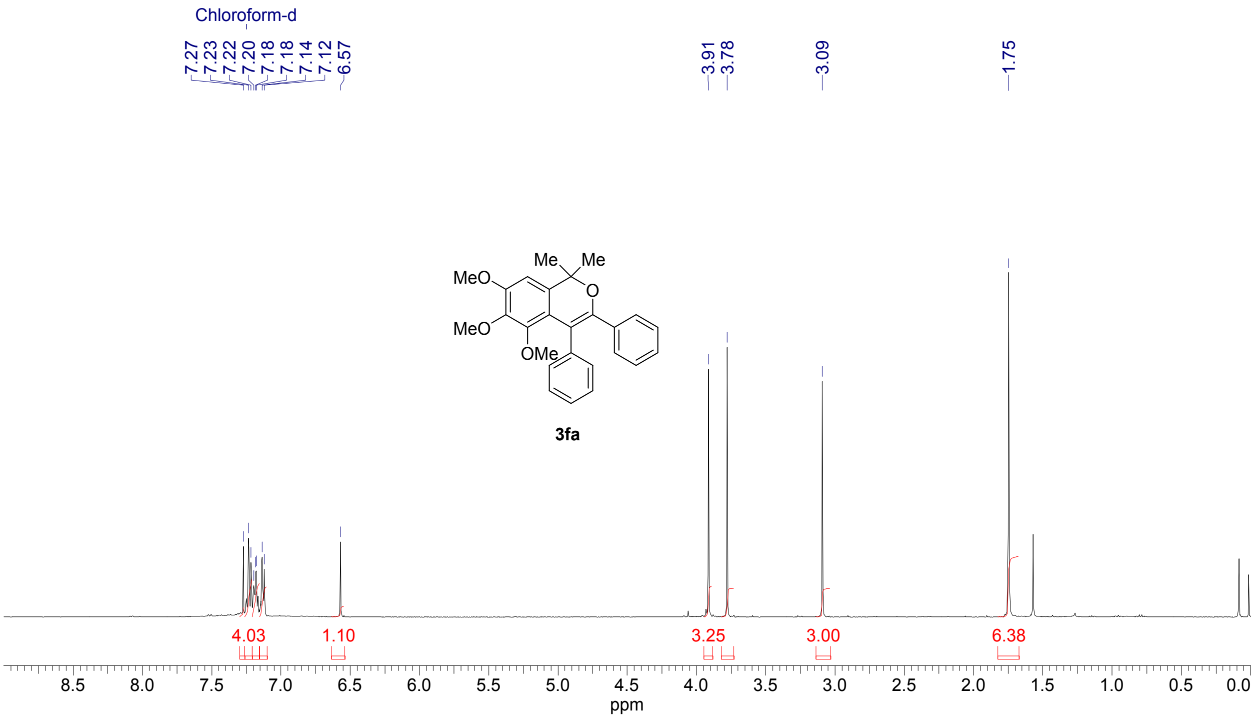

^{1}H-NMR (400 MHz) spectrum of **3fa** in $CDCl_3$

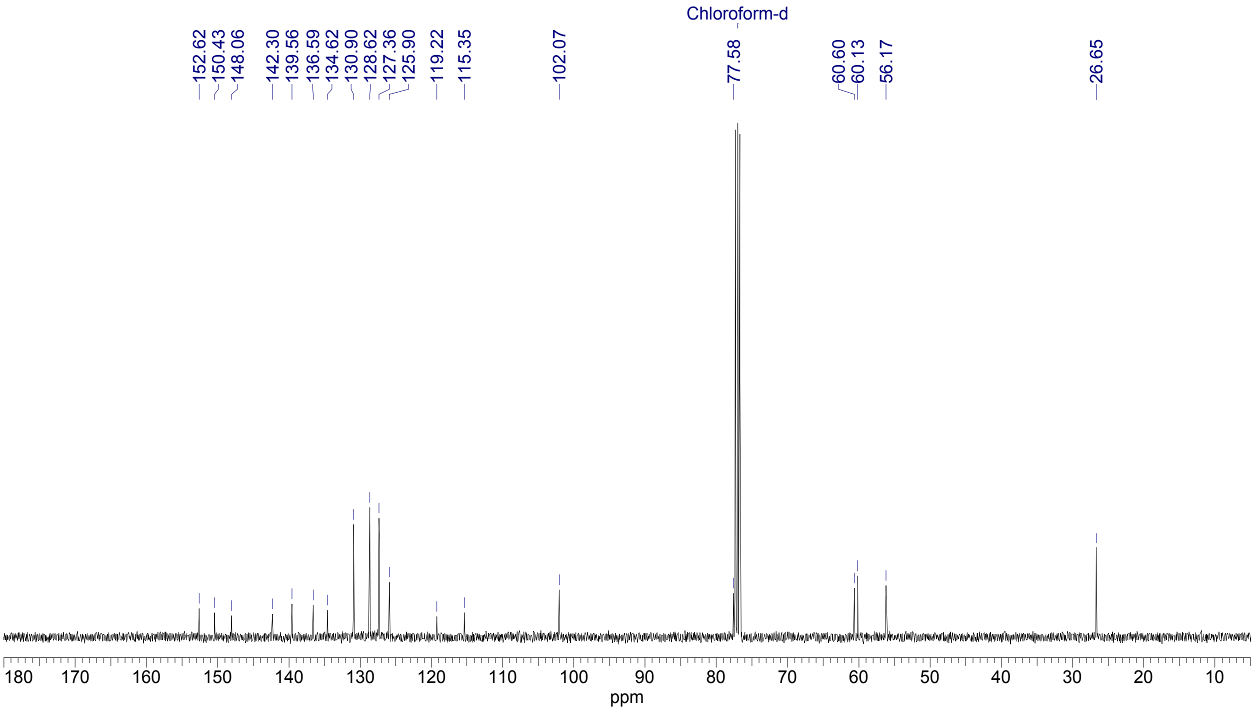

$^{13}C\{^{1}H\}$-NMR (100 MHz) spectrum of **3fa** in $CDCl_3$

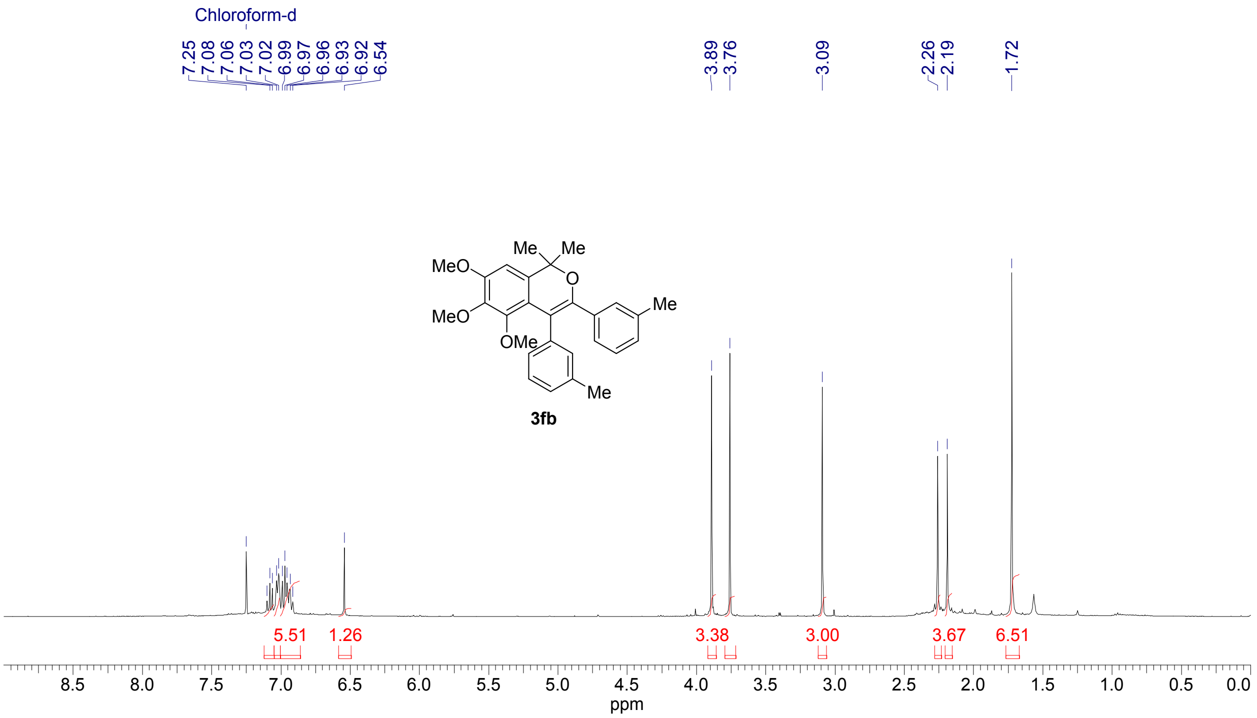

^{1}H-NMR (400 MHz) spectrum of **3fb** in $CDCl_3$

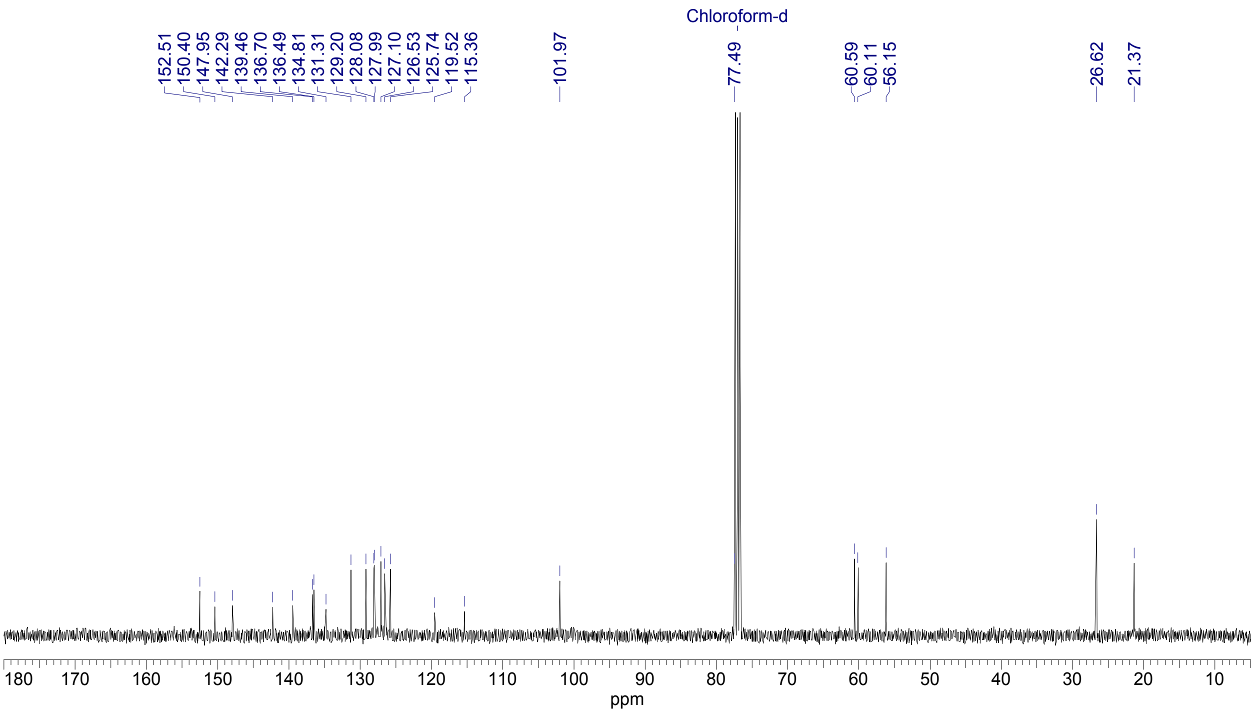

^{13}C{^{1}H}-NMR (100 MHz) spectrum of **3fb** in $CDCl_3$

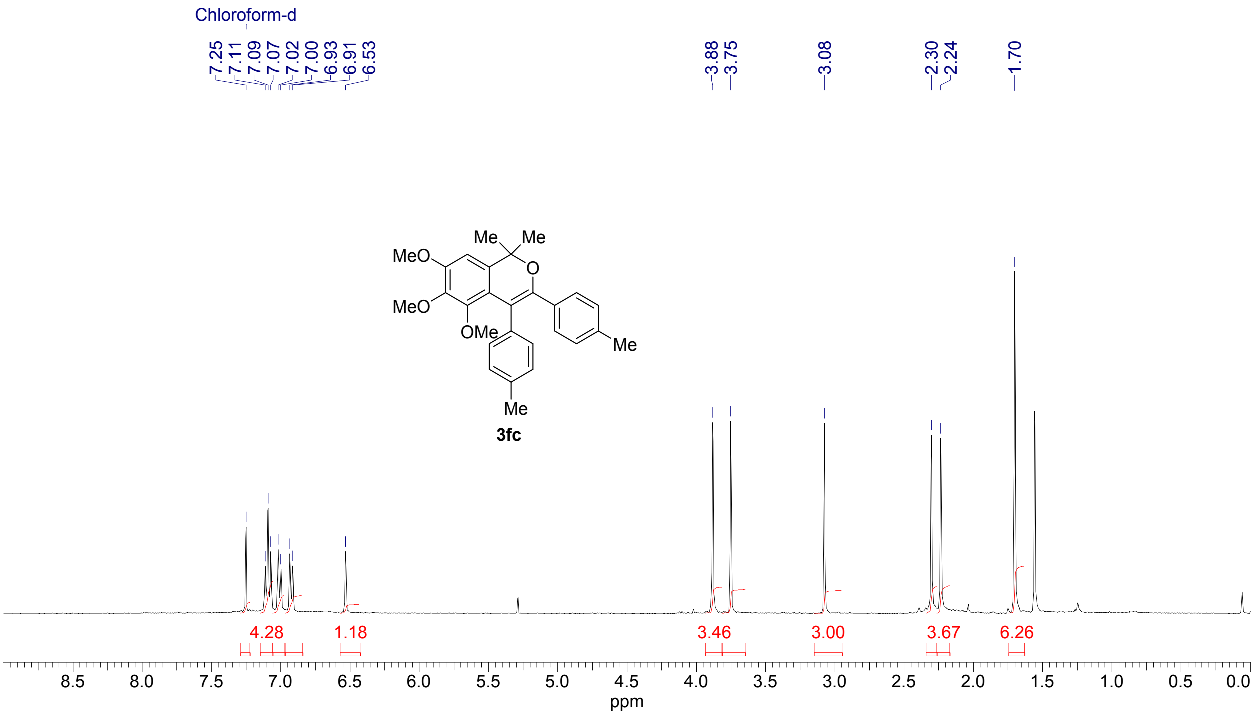

^{1}H-NMR (400 MHz) spectrum of **3fc** in $CDCl_3$

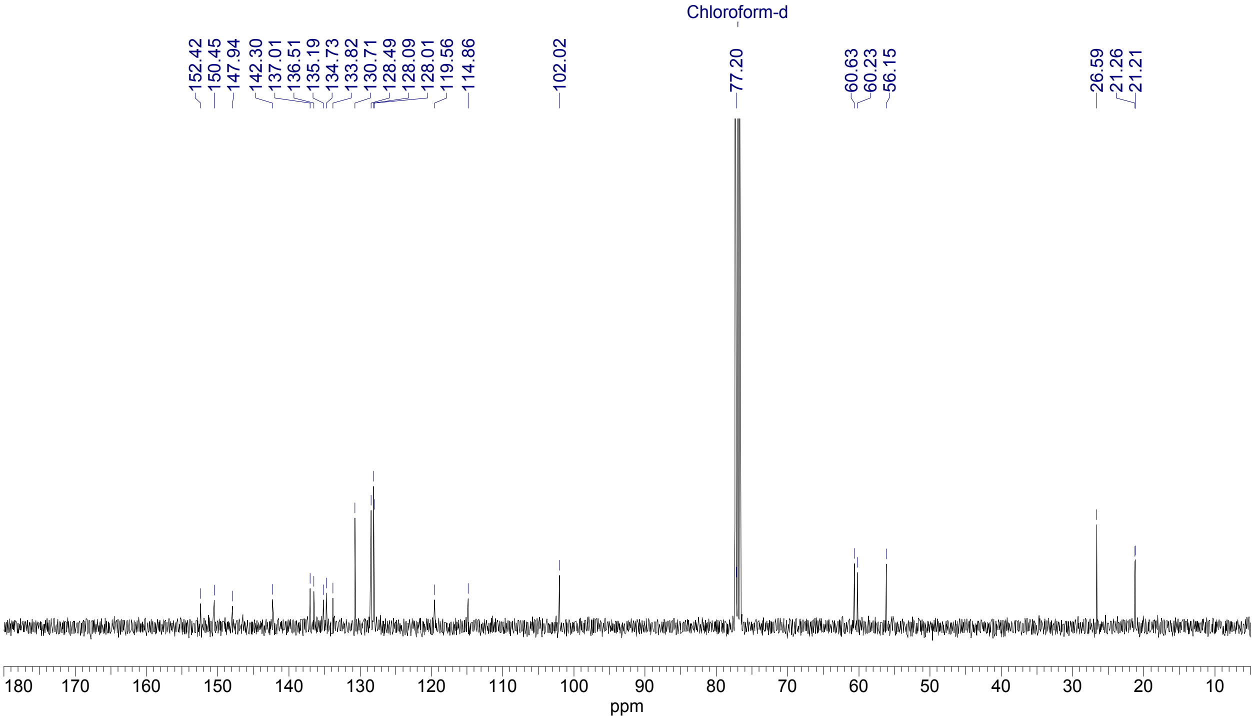

$^{13}C\{^{1}H\}$-NMR (100 MHz) spectrum of **3fc** in $CDCl_3$

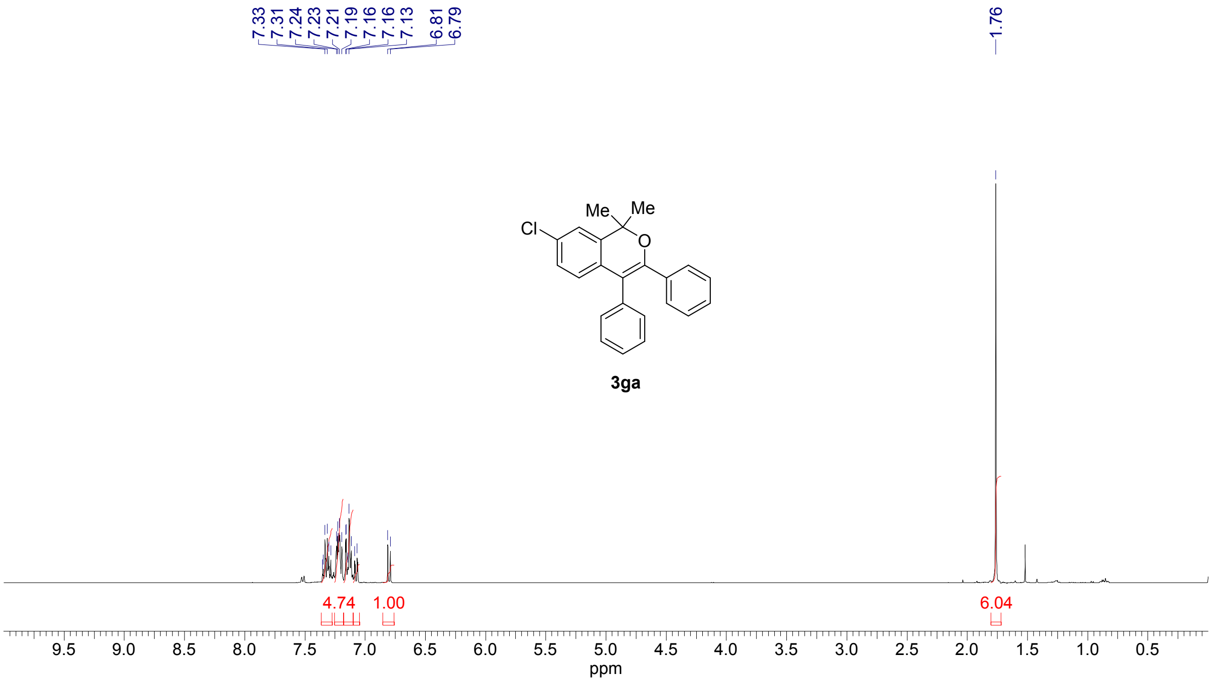

^{1}H-NMR (400 MHz) spectrum of **3ga** in $CDCl_3$

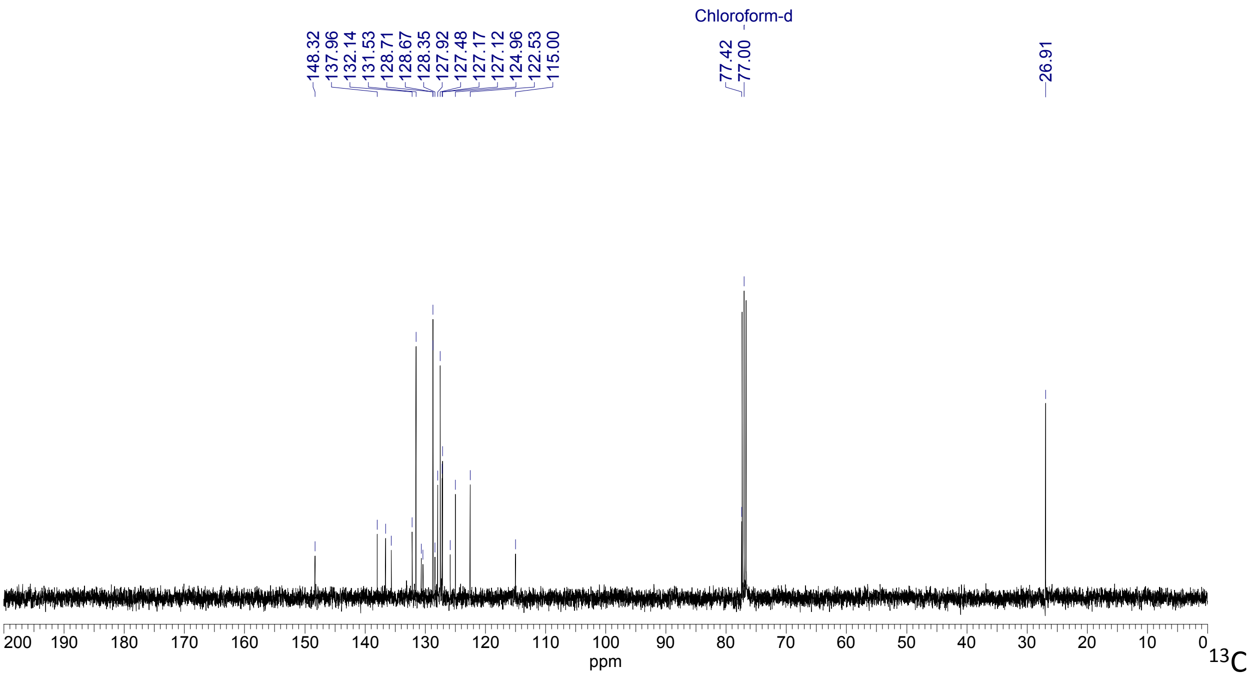

{^{1}H}-NMR (100 MHz) spectrum of **3ga** in $CDCl_3$

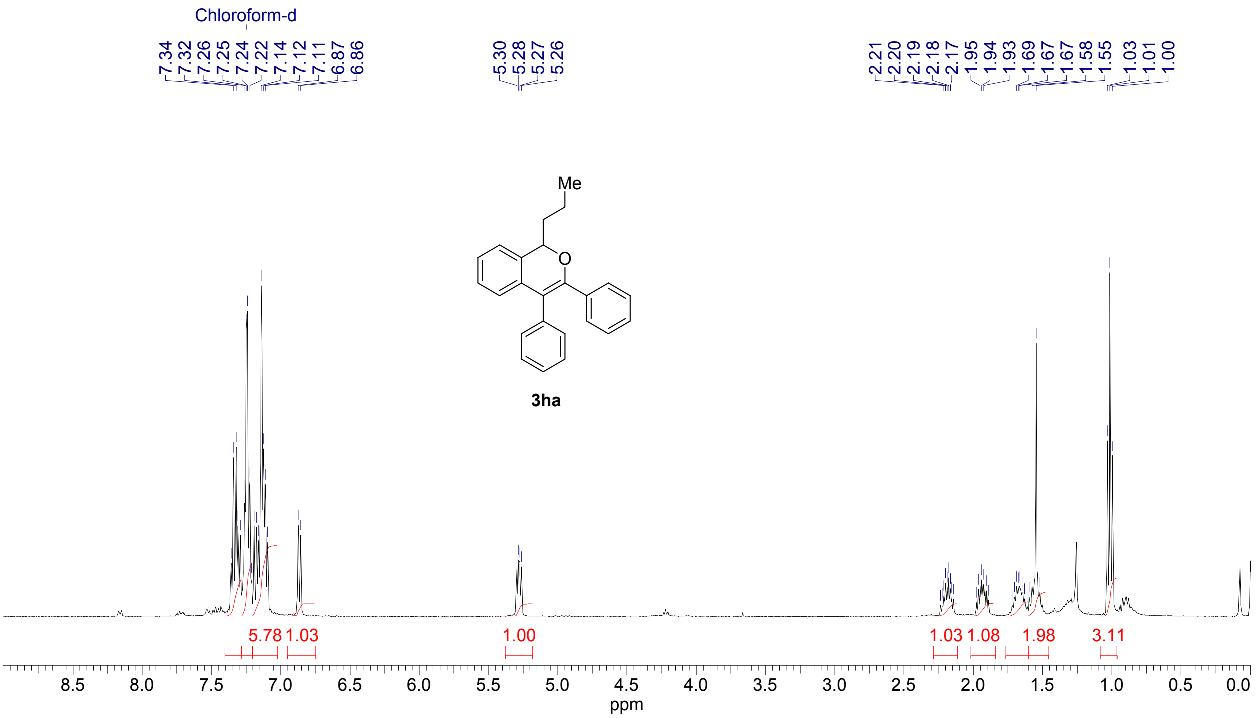

^{1}H-NMR (400 MHz) spectrum of **3ha** in $CDCl_3$

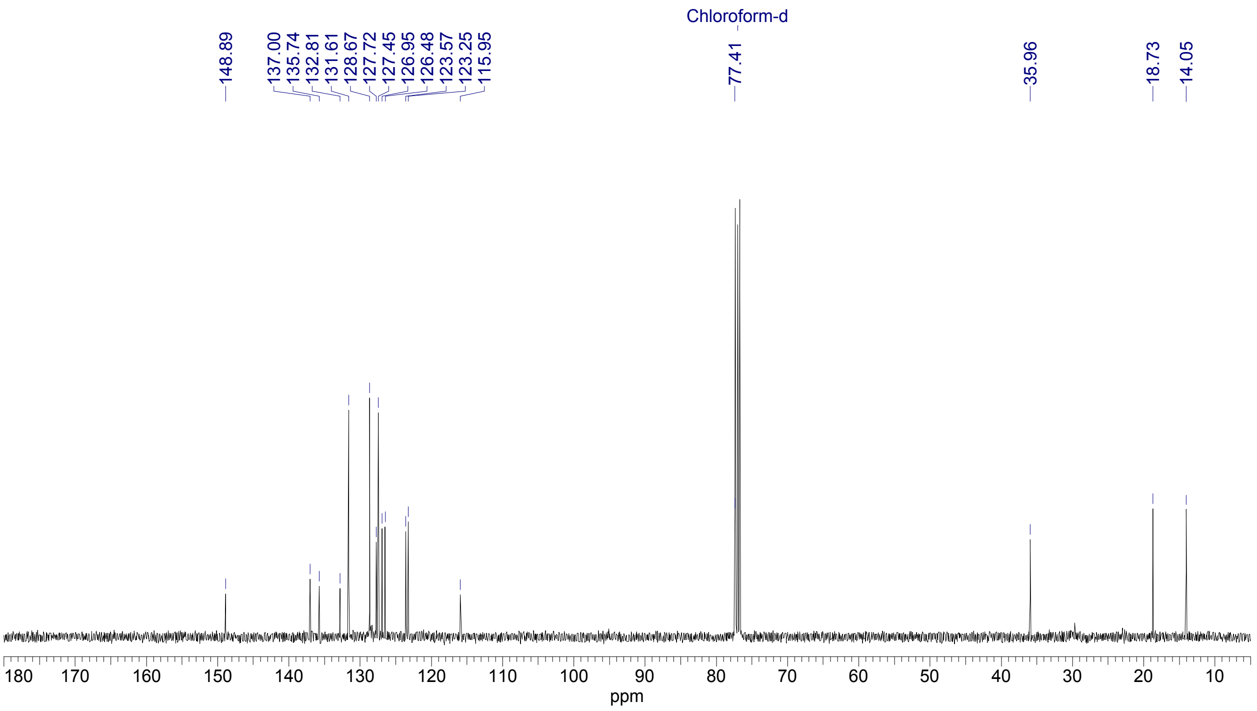

$^{13}C\{^{1}H\}$-NMR (100 MHz) spectrum of **3ha** in $CDCl_3$

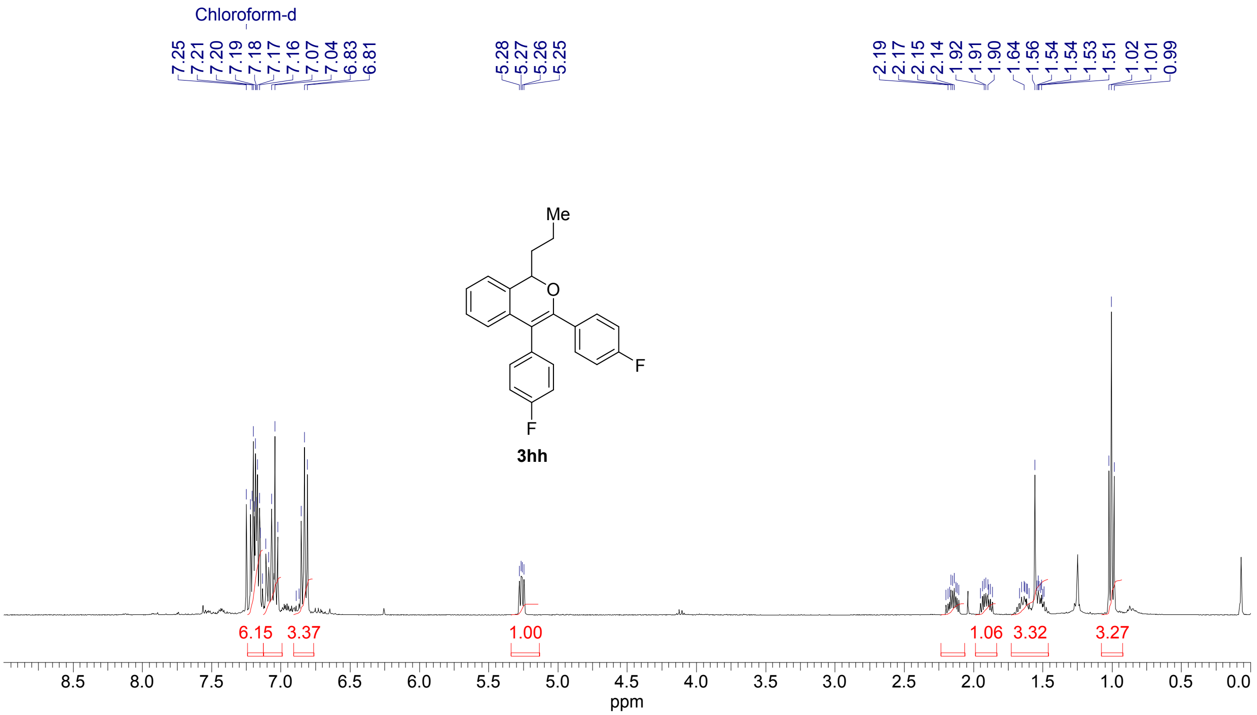

^{1}H-NMR (400 MHz) spectrum of **3hh** in $CDCl_3$

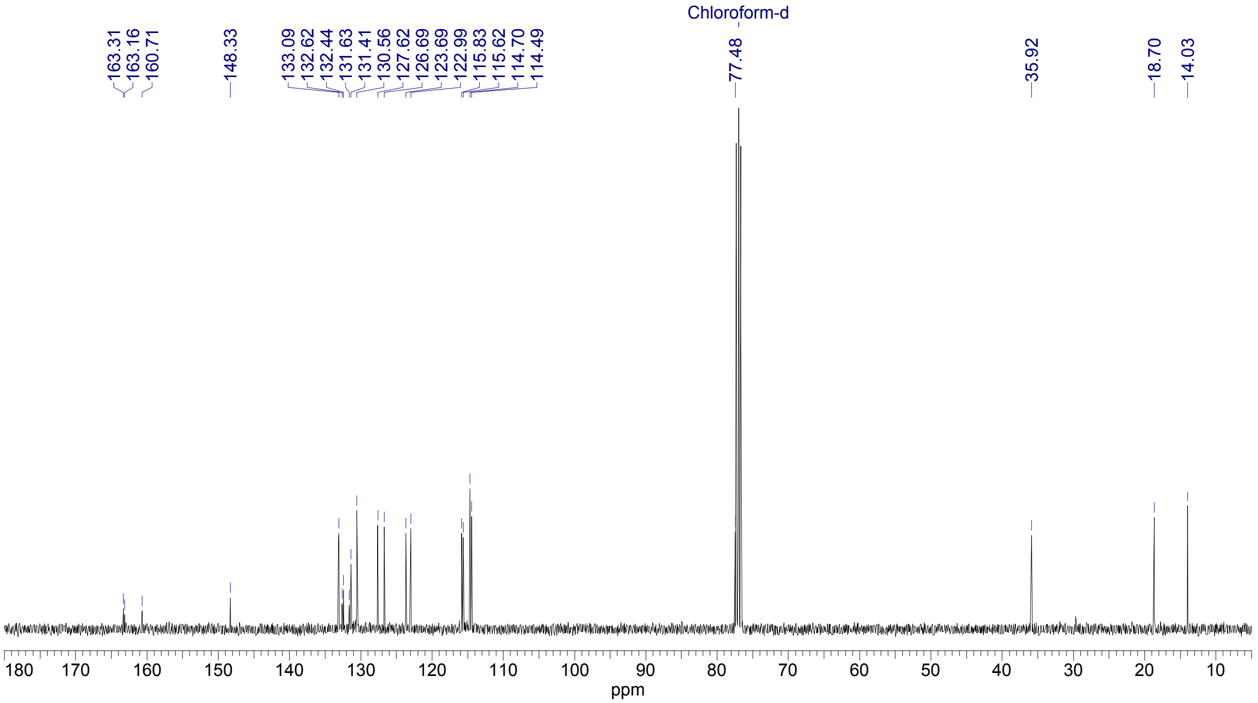

^{13}C{^{1}H}-NMR (100 MHz) spectrum of **3hh** in $CDCl_3$
